# Design, Synthesis, and Biological Evaluation of Indole-2-carboxamides as Potential Multi-Target Antiproliferative Agents

**DOI:** 10.3390/ph16071039

**Published:** 2023-07-22

**Authors:** Lamya H. Al-Wahaibi, Anber F. Mohammed, Mostafa H. Abdelrahman, Laurent Trembleau, Bahaa G. M. Youssif

**Affiliations:** 1 Department of Chemistry, College of Sciences, Princess Nourah bint Abdulrahman University, Riyadh 11564, Saudi Arabia; 2 Pharmaceutical Organic Chemistry Department, Faculty of Pharmacy, Assiut University, Assiut 71526, Egypt; anber_pharm_2006@yahoo.com; 3 Pharmaceutical Organic Chemistry Department, Faculty of Pharmacy, Al-Azhar University, Assiut 71234, Egypt; mhmyblatif@gmail.com; 4 School of Natural and Computing Sciences, University of Aberdeen, Meston Building, Aberdeen AB24 3UE, UK

**Keywords:** indole, antiproliferative, apoptosis, kinases, docking

## Abstract

A small set of indole-based derivatives, **IV** and **Va**–**I**, was designed and synthesized. Compounds **Va**–**i** demonstrated promising antiproliferative activity, with GI_50_ values ranging from 26 nM to 86 nM compared to erlotinib’s 33 nM. The most potent antiproliferative derivatives—**Va**, **Ve**, **Vf**, **Vg**, and **Vh**—were tested for EGFR inhibitory activity. Compound **Va** demonstrated the highest inhibitory activity against EGFR with an IC_50_ value of 71 ± 06 nM, which is higher than the reference erlotinib (IC_50_ = 80 ± 05 nM). Compounds **Va**, **Ve**, **Vf**, **Vg**, and **Vh** were further tested for BRAF^V600E^ inhibitory activity. The tested compounds inhibited BRAF^V600E^ with IC_50_ values ranging from 77 nM to 107 nM compared to erlotinib’s IC_50_ value of 60 nM. The inhibitory activity of compounds **Va**, **Ve**, **Vf**, **Vg**, and **Vh** against VEGFR-2 was also determined. Finally, in silico docking experiments attempted to investigate the binding mode of compounds within the active sites of EGFR, BRAF^V600E^, and VEGFR-2.

## 1. Introduction

Inhibiting oncogenic protein kinases [1,2,3] has been shown to be an effective anticancer strategy [4,5]. As of March 2019, The United States Food and Drug Administration (FDA) has approved a total of 43 small-molecule kinase inhibitors for the treatment of various cancers [6,7,8,9,10]. The majority of approved kinase inhibitors are for receptor tyrosine kinases, the best-validated of which are EGFR (epidermal growth factor receptor) [11] and VEGFR (vascular endothelial growth factor receptor) [12,13]. EGFR amplification or mutation is seen in a variety of cancers, with non-small-cell lung cancer (NSCLC) being the most common [14,15]. VEGFR activation is responsible for tumor angiogenesis/metastasis [16,17] and is associated with a poor prognosis in cancer patients [18,19]. NSCLC is commonly treated with EGFR inhibitors [20,21], as are kidney and thyroid cancers with VEGFR inhibitors [22,23,24].

**Figure 1:**
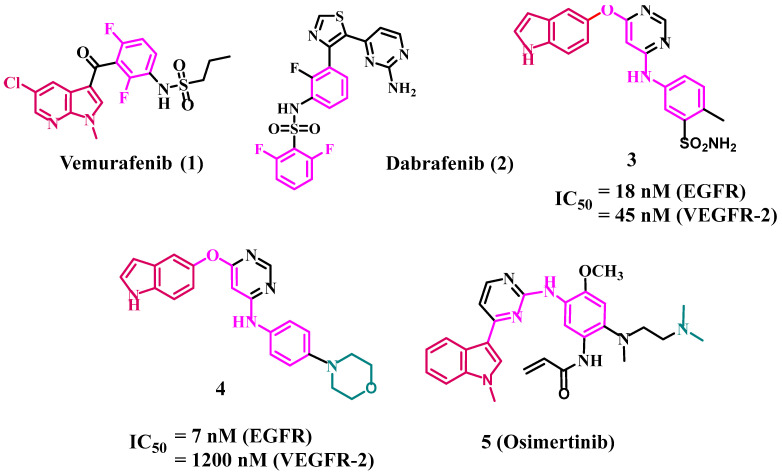
Structure of compounds **1**–**5**.

Protein kinase for serine/threonine Raf (rapid accelerated fibrosarcoma) [25,26], which includes A-Raf, B-Raf, and C-Raf, is a key player in the Ras/Raf/MEK/ERK (MAPK, mitogen-activated protein kinase) signaling pathway [27,28]. Via this pathway, growth signals from cell surface receptors (e.g., EGFR and VEGFR) to the nucleus promote cell proliferation, differentiation, and survival. B-Raf is the most frequently mutated Raf isoform in cancers [29]. Constitutively activated B-Raf^V600E^ is found in a variety of cancers, including leukemia, melanoma, thyroid cancer, and colorectal cancer [30,31,32]. Vemurafenib (1, Figure 1) [33] and dabrafenib (**2**, Figure 1) [34] are selective B-Raf^V600E^ inhibitors that have been approved for the treatment of advanced melanoma.

Signals from receptor tyrosine kinases (e.g., EGFR and VEGFR) can also be blocked by downstream Raf inhibition. Resistance to current B-Raf^V600E^ therapy, on the other hand, has been linked to EGFR signaling pathways [35,36] or VEGF-A upregulation [37,38]. The combination of the EGFR antibody cetuximab and drug **1** shows clinical benefits in patients with refractory B-Raf^V600E^ metastatic colorectal cancer [39,40,41]. In vivo, the combination of the B-Raf^V600E^ inhibitor PLX4720 and the VEGF antibody bevacizumab has synergistic effects [42,43]. These findings suggested that a small-molecule Raf inhibitor with EGFR/VEGFR inhibitory activity could be beneficial for cancers that are refractory to other treatments. Ding and colleagues [44] reported a new class of dual B-Raf/EGFR inhibitors in what became a leading study. The improved compound is effective against melanoma and/or colorectal cancers that are resistant to **1**.

The indole skeleton, which is found in many active substances and natural products, is one of the most well-known structures with impressive anticancer activity [45,46,47]. Many indole compounds have been found to be effective anticancer medicines, with some even being utilized in clinics [31,48,49,50]. A substantial number of indole-based compounds with TK inhibitory action were also discovered in the literature study [51,52,53].

Song et al. developed a set of indole derivatives that act as dual EGFR/VEGFR-2 inhibitors [54]. Compound **3** (Figure 1) inhibited EGFR and VEGFR-2 simultaneously, with IC_50_ values of 18 and 45 nM, respectively. Compound **4** (Figure 1) was also reported to be a dual EGFR/VEGFR-2 inhibitor, with a stronger effect against EGFR than **3**, indicating the importance of the morpholino moiety in EGFR inhibitory activity. Osimertinib (**5**, Figure 1) is an EGFR TKI that is approximately 200 times more selective for the mutant protein than for wild-type EGFR [53]. Osimertinib was approved by the FDA in 2015 to treat EGFR^T790M^-positive NSCLC due to its selectivity and activity [53,55,56].

**Figure 2:**
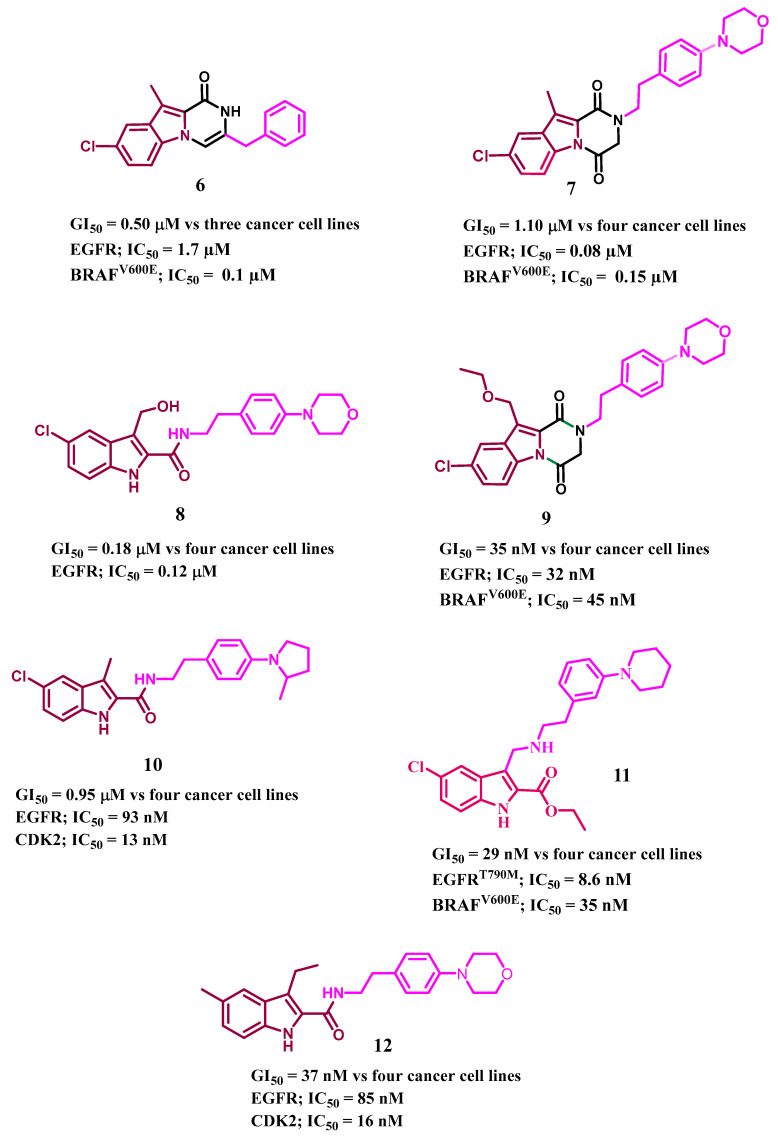
Structures of our previously reported indole-based kinase inhibitors **6**–**12**.

Recently, we reported on some indole derivatives that exhibited promising antiproliferative activity as dual or multikinase inhibitors [31,48,57,58,59,60,61]. Some of these compounds, targeted kinases, and their IC_50_ values are shown in Figure 2.

**Figure 3:**
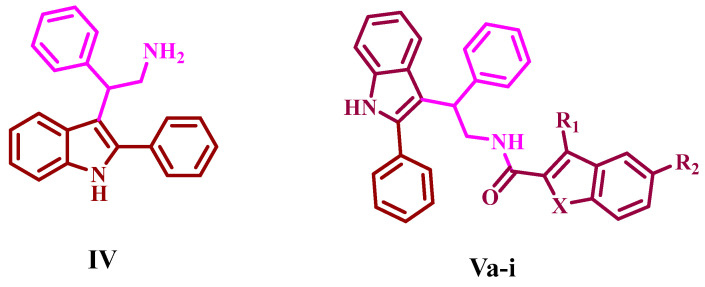
Structures of the newly synthesized indole-based kinase inhibitors **IV** and **Va**–**i**.

Motivated by the aforementioned data on the promising antiproliferative action of indole-based structures and as part of our ongoing effort to find dual or multi-targeted kinase agents, in this paper, we present the synthesis and antiproliferative activity of a small set of indole-based derivatives: **IV** and **Va**–**i** (Figure 3).

We tested the antiproliferative activity of the newly synthesized compounds against four different human cancer cell lines. The most promising derivatives were further investigated for multi-kinase inhibitory effects against EGFR, VEGFR-2, and BRAF^V600E^. In addition, caspase and apoptotic assay pathways were assessed. Finally, molecular docking studies of the most active compounds within the active sites of targeted kinases were performed to determine the binding modes of the evaluated compounds.

## 2. Results and Discussion

### 2.1. Chemistry

**Scheme 1:**
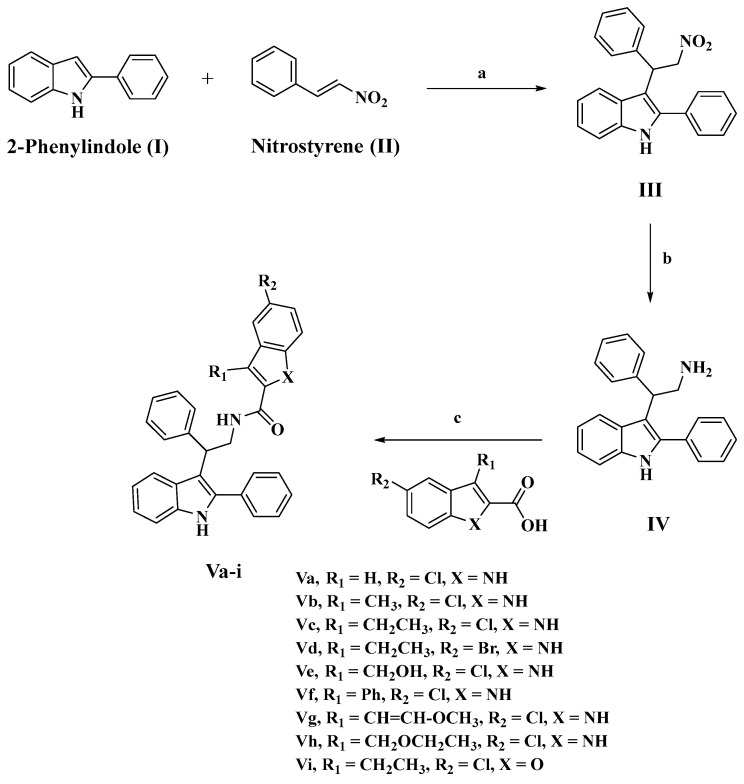
Synthesis of compounds **III** and **IV** and new targets **Va**–**i**. Reagent and reaction conditions: (**a**) sulfamic acid, methanol, reflux 12 h; (**b**) LAH, Et_2_O, 0 ^0^C to rt, overnight; (**c**) BOP, DIPEA, DCM, rt, overnight.

Scheme 1 depicts the synthesis of compounds **III**, **IV**, and **Va**–**i**. In the presence of catalytic amounts of sulfamic acid, 2-phenyl indole **(I)** and β-nitrostyrene **(II)** were reacted in refluxing methanol to produce 3-(2-nitro-1-phenylethyl)-2-phenyl-1*H*-indole **(III)**, which was then purified via flash chromatography on silica gel using EtOAc/hexanes (1:4) to give the desired product as an oil, which was solidified by being dissolved in DCM followed by addition of hexane. Then, compound **III** was reduced in diethyl ether (Et_2_O) under a nitrogen atmosphere using lithium aluminum hydride (LAH) to yield the desired product **IV** as a white solid. The structure of compound **IV** was confirmed using ^1^H NMR, ^13^C NMR, and HRESI-MS spectroscopy. The presence of characteristic signals of the ethanamine group (CHCH_2_NH_2_) was revealed in the ^1^H NMR spectrum of **IV** as doublet of doublet signal (1H) at *δ* 4.42 of CHCH_2_ (*J* = 9.7, 6.2 Hz), doublet of doublet signal (1H) at *δ* 3.58 of CHCH_2_a (*J* = 12.6, 9.8 Hz), doublet of doublet signal (1H) at *δ* 3.44 of CHCH_2_b (*J* = 12.7, 6.2 Hz), and singlet signal (2H) at *δ* 1.34 of the NH_2_ group, which was confirmed by the presence of two carbon signals at *δ* 46.58 and 46.55 in the ^13^C NMR spectrum of **IV**. Our HRESI-MS analysis of **IV** revealed the presence of a peak at *m/z* 313.1700, calculated for [M+H]^+^ C_22_H_21_N_2_: 313.1699.

The desired indole-2-carboxamides **Va**–**i** were obtained by coupling ethanamine **IV** with appropriate indole-2-carboxylic acids (**1**–**5**) in dichloromethane (DCM) using benzotriazol-1-yloxytris(dimethylamino)phosphonium hexafluorophosphate (BOP) and diisopropyl ethylamine (DIPEA). Various spectroscopic methods of analysis were used to confirm the structures of compounds **Va**–**i**. As an example, the ^1^H NMR spectrum of compound **Vg** (R_1_ = methoxyvinyl, R_2_ = Cl) revealed the presence of three signals corresponding to NH groups: a singlet signal at *δ* 9.79 ppm of indole NH, a singlet signal at *δ* 8.34 ppm of 2-phenylindole NH, and a triplet signal at *δ* 6.54 ppm of amidic NH. Moreover, the spectrum revealed methoxyvinyl group characteristic signals in the form of two doublets of 1H each *δ* 6.59 ppm and *δ* 5.15 ppm corresponding to CH=CHOCH_3_ and CH=CHOCH_3_, respectively, and singlet signal of 3H at δ 3.15 ppm of OCH_3_. Also, the **Vg** spectrum was characterized by the presence of signals corresponding to CHCH_2_ group, in the form of *δ* 4.78 (dd, *J* = 10.3, 6.3 Hz, 1H, CHCH_2_), *δ* 4.71–4.64 (m, 1H, CHCH_2_a), and *δ* 4.13–4.01 (m, 1H, CHCH_2_b). Our HRESI-MS analysis of **Vg** revealed the presence of a peak at *m/z* 546.1946, calculated for [M+H]^+^ C_34_H_29_ClN_3_O_2_.

### 2.2. Biology

#### 2.2.1. Assay for Cell Viability

To assess the viability of compounds **IV** and **Va**–**i**, the human mammary gland epithelial (MCF-10A) cell line was exploited [62,63,64]. Compounds **IV** and **Va**–**i** were cultured for four days on MCF-10A cells before being evaluated for vitality using the MTT assay. According to Table 1, none of the compounds examined displayed cytotoxic actions, and the cell viability for the compounds tested at 50 µM was greater than 89%.

#### 2.2.2. Antiproliferative Assay

Using the MTT assay [59,65] and erlotinib as the reference drug, the antiproliferative activity of **Va**–**i** was assessed against four human cancer cell lines: a pancreatic cancer (Panc-1) cell line, breast cancer (MCF-7) cell line, colon cancer (HT-29) cell line, and human epithelial (A-549) cancer cell line. The median inhibitory concentration (IC_50_) is shown in Table 1.

Compound **IV** (the amine derivatives) was the least potent of all the synthesized compounds, with a GI_50_ value of 104 nM against the four cancer cell lines tested, approximately three-fold less potent than the reference erlotinib, which has a GI_50_ value of 33 nM. In contrast to compound **IV**, compounds **Va**–**o** demonstrated promising antiproliferative activity, with GI_50_ values ranging from 26 nM to 86 nM against the cancer cell lines tested compared to erlotinib’s 33 nM. In all cases, compounds **Va**–**o** were more potent than compound **IV**, indicating the importance of the second indole or benzofuran moiety for activity.

Compounds **Va** and **Ve**–**h** were the most effective antiproliferative agents, with GI_50_ ranging from 26 nM to 48 nM. Compound **Va** (R_1_ = H, R_2_ = Cl, X = NH) was the most potent derivative, with a GI_50_ value of 26 nM, being 1.3-fold more potent than the reference erlotinib. Compound **Va** outperformed erlotinib against all cancer cell lines tested. 

Compound **Vg** (R_1_ = CH=CH-O-CH_3_, R_2_ = Cl, X = NH) ranked second in terms of antiproliferative action with a GI_50_ value of 31 nM, and it was comparable to the reference erlotinib but more potent against the breast cancer cell line (MCF-7) **(**Table 1). Compound **Vh** (R_1_ = CH_2_-O-CH_2_CH_3_, R_2_ = Cl, X = NH) revealed promising antiproliferative action with a GI_50_ value of 37 nM against the four cancer cell lines tested, which is 1.4-fold less potent than compound **Va**.

Compounds **Ve** (R1 = CH_2_OH, R_2_ = Cl, X = NH) and **Vf** (R_1_ = Ph, R_2_ = Cl, X = NH) demonstrated promising antiproliferative activities, with GI_50_ values of 44 nM and 48 nM, respectively, which were 1.3-fold and 1.45-fold less potent than the reference erlotinib. These findings showed that the type and nature of the substituent on the third position of the indole/benzofuran moiety is required for activity and that it increased in the order H > methoxyvinyl > ethoxymethyl > hydroxymethyl > phenyl.

Compound **Vc** (R_1_ = CH_2_CH_3_, R_2_ = Cl, X = NH) demonstrated good antiproliferative activity against the four cancer cell lines tested, with a GI_50_ value of 56 nM, which is 2.2-fold less potent than **Va** (R_1_ = H, R_2_ = Cl, X = NH). Furthermore, compound **Vb** (R_1_ = CH_3_, R_2_ = Cl, X = NH) showed an effect that was comparable to that of the 3-ethyl derivative, **Vc**, with a GI_50_ of 59 nM, which is also less potent than **Va**, confirming the importance of the 3-substitution on the second indole moiety.

The substitution of a bromine atom for the chlorine atom in compound **Vd** (R_1_ = CH_2_CH_3_, R_2_ = Br, X = NH) resulted in a significant decrease in the antiproliferative activity of compound **Vd**, which had a GI_50_ value of 66 nM (1.2-fold less potent than the chloro derivative **Vc**), indicating that the chlorine atom is more tolerated for the antiproliferative action than the bromine one.

Finally, the benzofuran derivative **Vi** (R_1_ = CH_2_CH_3_, R_2_ = Cl, X = O) exhibited the least potent activity among **Va**–**o** derivatives, with a GI_50_ value of 86 nM, which is 1.5-fold less potent than the indole derivative **Vd** (R_1_ = CH_2_CH_3_, R_2_ = Cl, X = NH), indicating the importance of the indole NH group in antiproliferative action.

#### 2.2.3. EGFR Inhibitory Assay

The antiproliferative compounds with the highest potency (Va, Ve, Vf, Vg, and Vh) were further examined for their suppressive action against EGFR as a probable target for their antiproliferative activity [59,66]. Table 2 displays the results as IC_50_ values. This test’s results are comparable with those of the antiproliferative assay, with compound Va (R_1_ = H, R_2_ = Cl, X = NH) displaying the strongest inhibitory activity against EGFR (IC_50_ = 71 ± 06 nM), which is greater than the reference erlotinib (IC_50_ = 80 ± 05 nM).

With an IC_50_ value of 79 ± 06 nM, compound Vg (R_1_ = CH=CH-O-CH_3_, R_2_ = Cl, X = NH) is the second most active compound, followed by compound Vh (R_1_ = CH_2_-O-CH_2_CH_3_, R_2_ = Cl, X = NH), which has an IC_50_ value of 85 ± 08 nM. Compounds Ve and Vf showed moderate EGFR inhibitory activity with IC_50_ values of 94 ± 07 nM and 103 ± 08 nM, respectively.

These results indicate that EGFR may be a potential target for compounds **Va**, **Vg**, and **Vh**, which may require additional structural modifications in the future to optimize their effects.

#### 2.2.4. BRAF^V600E^ Inhibitory Assay

Compounds **Va**, **Ve**, **Vf**, **Vg**, and **Vh** were further tested for BRAF^V600E^ inhibitory activity, and the results are shown in Table 2 as IC_50_ values [31,67]. The results showed that the tested compounds inhibited BRAF^V600E^ with IC_50_ values ranging from 77 nM to 107 nM; erlotinib’s IC_50_ value was 60 nM.

Compound **Va** (R_1_ = H, R_2_ = Cl, X = NH), which was the most potent derivative in both antiproliferative and EGFR inhibitory assays, was also the most potent derivative BRAF^V600E^ inhibitor, showing an IC_50_ value of 67 ± 5 nM, which was less potent than erlotinib (IC_50_ = 60 ± 5 nM). **Vg** (R_1_ = CH=CH-O-CH_3_, R_2_ = Cl, X = NH) and **Vh** (R_1_ = CH_2_-O-CH_2_CH_3_, R_2_ = Cl, X = NH) ranked second and third as anti-BRAF^V600E^ compounds, with IC_50_ values of 83 ± 6 nM and 89 ± 7 nM, respectively. These findings show that compounds **Va**, **Vg**, and **Vh** have potent antiproliferative activity and may act as dual EGFR and BRAF^V600E^ inhibitors.

#### 2.2.5. VEGFR-2 Inhibitory Assay

The upregulation of many kinases in endothelial cells showed a critical involvement in cancer angiogenesis and vasculogenesis [68,69]. VEGFR-2 is one of these protein kinases that are involved in endothelial cell survival/proliferation and cancer development [70,71]. The inhibitory activity of compounds **Va**, **Ve**, **Vf**, **Vg**, and **Vh** against VEGFR-2 was determined utilizing kinase-glo-luminescent kinase assays with sorafenib as the reference drug [63,72]. Table 2 presents the results.

In general, the investigated compounds showed good VEGFR-2 inhibitory activity, with IC_50_ values ranging from 1.10 nM to 3.25 nM, whereas the reference sorafenib had an IC_50_ value of 0.17 nM. Compounds **Ve** (R_1_ = CH_2_OH, R_2_ = Cl, X = NH) and **Vg** (R_1_ = CH=CH-O-CH_3_, R_2_ = Cl, X = NH) revealed the most potent VEGFR-2 inhibitory effects, with IC_50_ values of 1.10 nM and 1.60 nM, respectively, which were six-fold less potent than the reference sorafenib. The most potent derivative in the antiproliferative, EGFR, and BRAF^V600E^ inhibitory assays, compound **Va** (R_1_ = H, R_2_ = Cl, X = NH), had a promising inhibitory effect on VEGFR-2 with an IC_50_ value of 2.15 ± 0.20 nM. Finally, compound **Vg** (R_1_ = CH=CH-O-CH_3_, R_2_ = Cl, X = NH) showed excellent VEGFR-2 inhibitory efficacy with an IC_50_ value of 3.25 nM, although this was two-fold less potent than compound **Ve**.

The antiproliferative, EGFR, BRAF^V600E^, and VEGFR-2 results showed that compounds **Va**, **Ve**, **Vg**, and **Vh** can display substantial antiproliferative effects and that they may be able to operate as multi-kinase inhibitors, making them viable lead compounds for additional structural modifications.

### 2.3. Apoptotic Marker Assays

The development of innovative apoptosis-targeting drugs has become crucial for therapeutic application, as apoptotic abnormalities in cancer cells are the most significant barrier to anticancer therapy efficacy [73,74,75,76]. To reveal their proapoptotic potential, compounds **Va**, **Ve**, **Vg**, and **Vh** were investigated for their ability to trigger the apoptosis cascade.

#### 2.3.1. Caspase 3 Assay

Caspases play a crucial function in the induction and completion of apoptosis. Caspase-3 is an essential caspase that cleaves different proteins in cells, resulting in apoptosis [77,78,79]. The most effective derivatives, compounds **Va**, **Ve**, **Vg**, and **Vh**, were evaluated as caspase-3 activators against a human epithelial cancer cell line (A-549) [73], and the findings are reported in Table 3.

The results showed that the studied compounds **Va**, **Ve**, **Vg**, and **Vh** had good caspase-3 overexpression levels, ranging from 460 ± 4 up to 726 ± 6 pg/mL in comparison to untreated control cells, which had a caspase-3 level of 66 pg/mL. Compounds **Va** and **Vg** displayed outstanding caspase-3 protein overexpression levels of 726 ± 6 and 528 ± 5 pg/mL, respectively. They elevated the protein caspase-3 in the human epithelial cancer cell line (A-549) by roughly **11** and **8** times more than the untreated control cells, and they were even more active than the reference doxorubicin (505 ± 4.0 pg/mL). These findings suggest that the compounds tested operate as caspase-3 activators and can thus be classified as apoptotic inducers.

#### 2.3.2. Caspase-8, Bax, and Bcl-2 Level Assays

As indicated in Table 3, compounds **Va** and **Vg** were investigated further for their effect on caspase-8, Bax, and Bacl-2 levels against the human epithelial (A-549) cancer cell line using doxorubicin as a control. The results showed that, compared to doxorubicin, all of the tested compounds significantly elevated caspase-8 and Bax levels.

Caspase-8 over-expression was highest in compound **Va** (3.50 ng/mL), followed by compound **Vg** (2.20 ng/mL), and finally the reference doxorubicin (1.80 ng/mL). Furthermore, **Va** and **Vg** showed 45- and 35-fold higher Bax induction (410 pg/mL and 320 pg/mL) than untreated A-549 cancer cells, respectively, while doxorubicin (280 pg/mL) showed a 31-fold induction.

Finally, versus doxorubicin, **Va** and **Vg** elicited the equipotent downregulation of anti-apoptotic Bcl-2 protein levels in the A-549 cell line (Table 3).

### 2.4. Molecular Modeling

A computational docking study was performed for compounds **IV** and **Va**–**i** to investigate their binding interactions with the epidermal growth factor receptor tyrosine kinase EGFR. Molecular Operating Environment (MOE) software [80,81] was used along with the crystal structure of the EGFR in complex with erlotinib (PDB: 1M17) [59,82]. All minimizations were completed using the force field (OPLS-AA) as well as Born solvation. The protein structure was protonated and corrected prior to the docking experiment.

**Figure 4:**
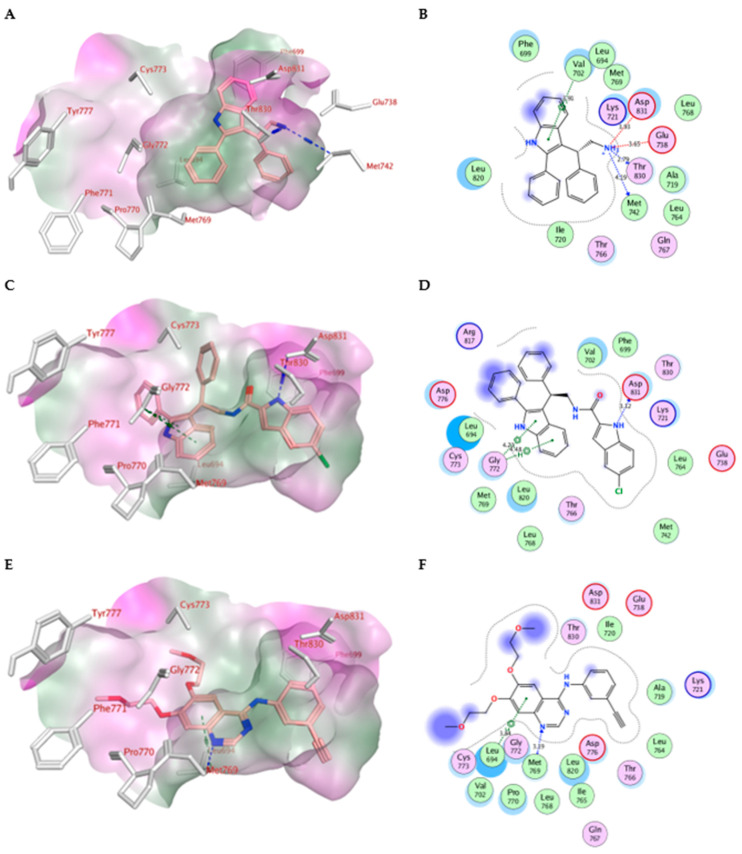
Docking representation models of compound **IV** and **Va** and erlotinib within the binding site of EGFR (ionic: red dashed lines, H-bond: blue dashed lines, Pi-H; green dashed lines); (**A**) 3D-docked model of compound **IV** (pink) showing the lipophilicity protein surface (hydrophilic: purple, neutral: white, lipophilic: green); (**B**) 2D-docked model of compound **IV**; (**C**) 3D-docked model of compound **Va** (cyan) showing the protein surface (gray); (**D**) 2D-docked model of compound **Va**; (**E**) 3D-docked model of compound **erlotinib** (pink) showing the protein surface (gray); (**F**) 2D-docked model of compound **erlotinib**.

The docking protocol was validated through redocking the co-crystallized erlotinib within the EGFR active site as the *S* score achieved by the docked ligand was -10.70 kcal/mol with *RMSD* value of 1.48 Å (Figure S1), Table 4. Based on the docking score analysis, the most effective antiproliferative compounds, **Va, Ve, Vf, Vg,** and **Vh**, exhibited the highest negative values, ranging from −9.89 to −10.52 kcal/mol compared with erlotinib’s score of −10.70 kcal/mol. However, the least potent amine derivative, **IV**, had the lowest docking score (−7.79 kcal/mol) amongst all compounds. After inspecting the ligand protein complexes, it was revealed that compound **IV** missed essential binding interactions within the large binding site (Figure 4A,B). The ligand 2-phenylindole scaffold bound deeply into the hydrophobic pocket forming stacking with Phe699 and pi-H interaction with Val702 (3.91 Å) in a way that was analogous to the erlotinib phenyl acetylene moiety. Also, the ligand amino group forms ionic interactions with Asp831 and Glu738 (3.93 and 3.65 Å, respectively) and donates H-bond interactions to Thr830 and Met742 with 2.79 and 4.19 Å, respectively. However, the ligand neither interacts with the key amino acid Met769 nor forms interactions at the gate of binding site. The introduction of a 5-haloindole moiety in compounds **Va**–**h** via amide linkage improved the ligand binding profile within the protein active site. The ligand 5-haloindole moiety, instead, inserted deeply into the hydrophobic pocket forming stacking with Phe699 and pi-H interaction with Leu820. In addition, the 5-haloindole NH donates H-bond interactions to Asp831 in compounds **Va**–**h**, with the exception of compound **Ve**, where the 3-hydroxymethylene group donates similar H-bond interactions to Asp831 with 3.09 Å. The latter interaction was missed in compound **Vi**, with the 5-halobenzofuran moiety indicating the significance of the indole NH moiety for optimally fitting the ligand within the active site. Moreover, the protein pocket accommodates both chlorine and bromine atoms at the fifth position of the indole moiety as the ligand forms additional halogen bond interactions with Leu764 and/or Thr766 at the pocket hinge. Interestingly, the substitution pattern at the third position of the 5-haloindole moiety has a significant impact on the VDW interaction surface of protein. The unsubstituted compound **Va** with R_1_= H and compound **Vg** with planar group R_1_= CH=CH-OCH_3_ showed the best fitting within the protein interaction surface. In contrast, with bulkier groups (R_1_= CH_3_, CH_2_CH_3_, CH_2_CH_3_, CH_2_OH, Ph, CH_2_OCH_2_CH_3_, respectively), compounds **Vb**, **Vc**, **Vd**, **Ve**, **Vf**, and **Vh** projected out of the protein interaction surface, destabilizing their overall binding complexes. Finally, the ligand 2-phenyl indole moiety stacked with Phe771 past the erlotinib ether linkages forming pi-H interactions with Leu694 and Gly772 in addition to other hydrophobic interactions with surrounding residues Gly772, Lys721, Leu694, Asp831, and Val702 at the gate of the binding site. (Figure 4C–F). Also, the 2-phenyl indole NH moiety in compounds **Vb** and **Vf** donates H-bond interactions to Asp776 with 2.99 and 2.90 Å, respectively.

**Figure 5:**
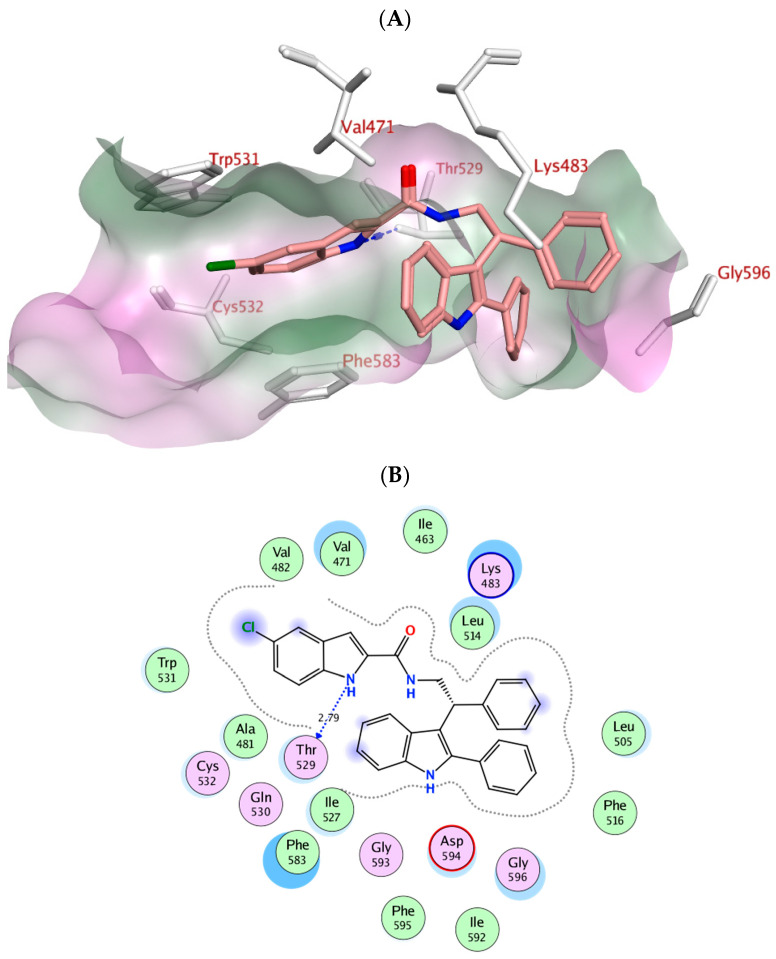
Docking representation model for compound Va; (**A**) 3D-docked model of compound Va (pink) within the active site of BRAF^V600E^ showing the lipophilicity protein surface (hydrophilic: purple, neutral: white, lipophilic: green); (ionic: red dashed lines, H-bond: blue dashed lines, Pi-H; green dashed lines); (**B**) 2D-docked model of compound Va within the active site of BRAF^V600E^ showing interatomic distances.

The foremost active antiproliferative compounds, **Va**, **Ve**, **Vf**, **Vg**, and **Vh**, were docked in silico to study their binding modes within the active site of BRAF^V600E^ using the crystal structure of the BRAF^V600E^ in complex with Vemurafenib (PDB: 3OG7) [83,84]. The docking results within the protein binding site were validated by redocking the co-crystallized vemurafenib showing an *S score* of −11.78 kcal/mol with a *RMSD* value of 0.96 Å (Figure S2) (Table 5). Our analysis of the docking scores of the examined compounds showed that compounds **Va**, **Vg**, and **Vh** exhibited better scores than compounds **Ve** and **Vf**. After investigating the binding modes, it was revealed that the most potent compound **Va** (R_1_ = H, R_2_ = Cl, X = NH) extended snugly within the protein active site (Figure 5A,B). The compound probes the space of the active site where the ligand 5-chloro-indole moiety stacks between Phe583 and Trp531 within the hydrophobic pocket forming hydrophobic interactions with Val471, Trp531, Phe583, Cys532, Ile463, and Thr592. Also, the 5-chloroindole moiety forms pi-H interactions with Val471 and/or Ile527 in compounds **Vf**, **Vg**, and **Vh**. In addition, the 5-chloro indole NH group donates H-bond interactions to the key amino acid residue Thr529 with (2.79 Å). Moreover, the chloro group was close to the key amino acid residue Cys532 at the site gate. Also, the ligand amide NH moiety forms weak H-bond interactions with the key amino acid Lys483. However, the unsubstituted derivative **Va** missed essential interactions with the amino acid residues Gln530, Cys532, Asp594, Gly596 compared with the co-crystallized ligand, Vemurafenib, at the binding site. The results of the docking simulations against EGFR explained the antiproliferative effects of compounds **IV** and **Va**–**i** relative to their binding affinity within the active site. Further simulations against BRAF^V600E^ suggest that compounds **Va**, **Vg**, and **Vh** might act as dual EGFR and BRAF^V600E^ kinase inhibitors.

**Figure 6:**
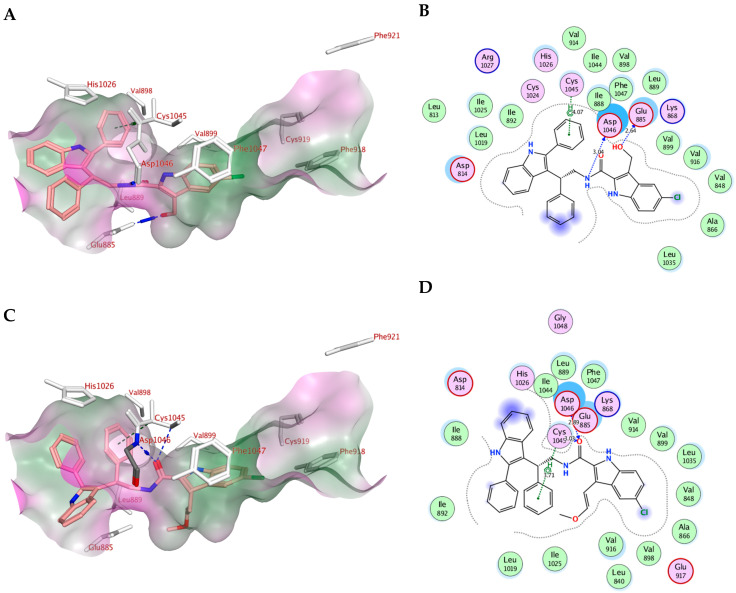
Docking representation models for compounds **Ve** and **Vg** within the active site of VEGFR-2 showing the lipophilicity protein surface (hydrophilic: purple, neutral: white, lipophilic: green); (ionic: red dashed lines, H-bond: blue dashed lines, Pi-H: green dashed lines); (**A**,**C**): 3D-docked models of compound **Ve** and **Vg** (pink); (**B**,**D**): 2D-docked models of compound **Ve** and **Vg** showing interatomic distances.

Moreover, the most potent antiproliferative compounds (**Va**, **Ve**, **Vf**, **Vg**, and **Vh**) were docked against vascular endothelial growth factor VEGFR-2 in order to investigate their potential multi-kinase inhibition, Figure 6. The crystal structure of VEGFR-2 in complex with sorafenib (PDB: 4ASD) [63] was used in the current study. The docking experiment was validated by redocking the co-crystallized ligand that showed an *S* score of -10.73 kcal/mol with a RMSD value of 0.46 Å, (Figure S3). All docked compounds showed good scores ranging from −8.18 to −9.77 kcal/mol compared with the co-crystallized ligand (Table 6). After examining the docked complexes, it was revealed that the compounds exhibited good fitting within the binding pocket. They showed comparable binding modes within the active site. At the hydrophobic gate of the binding site, the ligand 5-chloroindole scaffold stacked with Phe1047 and became surrounded by amino acid residues Phe918 and Phe921. On the opposite end of binding site, the 2-phenylindole moiety forms stacking with His1026 while making pi-H interactions with Cys1045 in the most potent compounds (**Ve** and **Vg**). However, the latter amino acid residue forms H-pi interactions with the 5-chloroindole moiety in compound **Vf**, Figure 6. Despite missing an interaction with key amino acid Cys919, the ligand amide linkage donates a H-bond to Glu885 while accepting a H-bond from Asp1046 in compounds **Va**, **Vf**, and **Vh**. However, in the case of the most potent compound, **Ve**, the ligand hydroxy methylene and the amide NH donate H-bond interactions to Glu885 and Asp1046 with 2.64 and 3.04 Å, respectively (Figure 3A,B). Compared with compound **Ve**, the amide carbonyl group of compound **Vg** accepts two H-bond interactions from Asp1046 and Cys1045 and misses an interaction with the key amino acid Glu885 due to its ether linkage (Figure 3C,D). The results of the docking simulations predicted the binding modes of the most active antiproliferative compounds and confirmed their potential multi-kinase inhibitory effects.

### 2.5. In Silico ADME/Pharmacokinetics Studies

In silico ADME studies were performed for compounds **IV** and **Va**–**i** using SwissADME [85,86] as well as ADMET lab tools [87,88]. The compounds’ SMILES (Simplified Molecule Input Line Entry Specification), obtained by using ChemDraw software, were entered as a list. The obtained pharmacokinetic data (Table 7) revealed that only compounds **IV** and **Va** are likely to be orally active as they obey Lipinski’s rules of five with zero or one violation, respectively. All tested compounds are expected to be a P-gp non-substrate. They are considered to be poorly absorbed by the intestine, except for compound **IV**. They are able to cross BBB with a probability ranging from 0.7 to 0.9. Most compounds exhibited limited permeability as indicated by logP values in the range of 4.25–7.59, except for compound IV. Concerning CYP inhibition, all compounds were predicted as inhibitors with a probability higher than 0.5, as shown in Table 7. The results predict that compounds **IV** and **Va**–**i** could exhibit acceptable pharmacokinetic and ADME properties (Table 7 and Table 8).

## 3. Materials and Methods

### 3.1. Chemistry

General Details: See Supplementary Materials.

#### 3.1.1. Synthesis of 3-(2-nitro-1-phenylethyl)-2-phenyl-1H-indole (III)

A mixture of 2-phenylindole (**I**) (0.53 g, 1.55 mmol, 1 eq), β-nitrostyrene (**II**) (0.25 g, 1.70 mmol, 1.1 eq), and sulfamic acid (0.02 g, 0.31 mmol, 0.2 eq) in methanol (30 mL) was heated at reflux for 12 h. After removing the solvent in vacuo, the residue was extracted with EtOAc, washed with saturated NaHCO_3_ solution and brine, dried over MgSO_4_, and evaporated under reduced pressure to give a crude product, which was subsequently purified via flash chromatography on silica gel using EtOAc/hexanes (1:4) to obtain the desired product as an oil. This oil was solidified by being dissolved in DCM, subjected to the addition of hexanes, and left overnight.

Yield % 79, mp 140-142 ^o^C. ^1^H NMR (400 MHz, Chloroform-*d*) *δ* 8.15 (s, 1H, indole NH), 7.62–7.06 (m, 14H, Ar-H), 5.34 (dd, *J* = 9.2, 7.1, 1H, CHCH_2_), 5.25–5.09 (m, 2H, CHCH_2_). ^13^C NMR (101 MHz, Chloroform-*d*) *δ* 139.89, 136.96, 136.06, 132.17, 128.95, 128.88, 128.79, 128.63, 127.47, 127.18, 127.02, 122.49, 120.31, 119.95, 111.42, 109.59, 79.08, 40.80. HRESI-MS *m/z* calcd. for [M-H]^-^ C_22_H_17_N_2_O_2_: 341.1296, found: 341.1290.

#### 3.1.2. Synthesis of 2-phenyl-2-(2-phenyl-1H-indol-3-yl)ethan-1-amine (IV)

For the suspension of lithium aluminum hydride (LAH) (2 g, 8.8 mmol, 10 equiv) in Et_2_O (30 mL) in a two-necked rounded bottom flask maintaining anhydrous conditions under nitrogen atmosphere at 0 °C, 3-(2-nitro-1-phenylethyl)-2-phenylindole (**III**) (0.3 g, 0.88 mmol, 1 eq) that had been dissolved in dry Et_2_O (10 mL) was added, and the reaction mixture was warmed to rt via stirring overnight. A saturated solution of Na_2_SO_4_ (5 mL) was slowly added at 0 °C, followed by stirring for an additional 30 min and the addition of H_2_O. The reaction mixture was filtered, and the residue was washed three times with Et_2_O. The combined filtrate was washed with brine, dried over MgSO_4,_ and evaporated under reduced pressure to yield the desired product **IV** (0.22 g, 80%) as a white solid.

Yield % 80, mp 167-169 ^o^C. ^1^H NMR (400 MHz, Chloroform-*d*) *δ* 8.28 (s, 1H, indole NH), 7.65 (d, *J* = 8.1 Hz, 1H, Ar-H), 7.53–7.33 (m, 8H, Ar-H), 7.35–7.16 (m, 4H, Ar-H), 7.09 (t, *J* = 8.1 Hz, 1H, Ar-H), 4.42 (dd, *J* = 9.7, 6.2 Hz, 1H, CHCH_2_), 3.58 (dd, *J* = 12.6, 9.8 Hz, 1H, CHCH_2_a), 3.44 (dd, *J* = 12.7, 6.2 Hz, 1H, CHCH_2_b), 1.34 (s, 2H, NH_2_). ^13^C NMR (101 MHz, Chloroform-*d*) *δ* 143.52, 137.23, 136.28, 132.92, 128.79, 128.77, 128.44, 128.10, 127.87, 127.68, 126.10, 122.18, 120.91, 119.86, 112.28, 111.11, 46.58, 46.55. HRESI-MS *m/z* calcd. for [M+H]^+^ C_22_H_21_N_2_: 313.1699, found: 313.1700.

#### 3.1.3. Synthesis of N-(2-phenyl-2-(2-phenyl-1H-indol-3-yl)ethyl)-1H-indole-2-carboxamides (Va-i)

A mixture of appropriate indole-2-carboxylic acid (0.40 mmol, 1 eq), BOP (0.27 g, 0.60 mmol, 1.5 eq), and DIPEA (0.11 mL, 0.80 mmol, 2 eq) in DCM (15 mL) was stirred for 10 min at rt before the addition of 2-phenyl-2-(2-phenyl-1*H*-indol-3-yl)ethanamine (0.15 g, 0.48 mmol, 1.2 eq), and the resulting reaction mixture was stirred overnight at rt. After removing the solvent in vacuo, the residue was extracted with EtOAc; washed with 5% HCl, saturated NaHCO_3_ solution, and brine; dried over MgSO_4_; and evaporated under reduced pressure to give a crude product, which was subsequently purified via flash chromatography on silica gel using EtOAc/hexanes (1:4) to yield the desired indole-2-carboxamides **Va**–**i**.

##### 5-Chloro-*N*-(2-phenyl-2-(2-phenyl-1H-indol-3-yl)ethyl)-1H-indole-2-carboxamide (**Va**)

Yield % 75, mp 112-114 °C. ^1^H NMR (400 MHz, Chloroform-*d*) *δ* 10.30 (s, 1H, indole NH), 8.39 (s, 1H, 2-phenylindole NH), 7.57–7.05 (m, 18H, Ar-H), 6.15 (t, *J* = 7.2 Hz, 1H, amide NH), 4.85 (dd, *J* = 9.8, 5.8 Hz, 1H, CHCH_2_), 4.63–4.51 (m, 1H, CHCH_2_a), 4.18–4.01 (m, 1H, CHCH_2_b). ^13^C NMR (101 MHz, Chloroform-*d*) *δ* 161.44, 141.84, 137.48, 136.42, 134.90, 132.26, 131.73, 128.79, 128.71, 128.56, 128.26, 128.23, 127.79, 127.26, 126.66, 125.91, 124.64,122.54, 120.82, 120.61, 120.15, 113.45, 111.50, 101.23, 43.35, 41.54. HRESI-MS *m/z* calcd. for [M+H]^+^ C_31_H_25_ClN_3_O: 490.1681, found: 490.1684.

##### 5-Chloro-3-methyl-*N*-(2-phenyl-2-(2-phenyl-1H-indol-3-yl)ethyl)-1H-indole-2-carboxamide (**Vb**)

Yield % 79, mp 135-137 °C. ^1^H NMR (400 MHz, DMSO-*d*_6_) *δ* 11.32–11.30 (m, 2H, indole NH, 2-phenylindole NH), 7.80 (t, *J* = 5.8 Hz, 1H, amide NH), 7.65–7.51 (m, 4H, Ar-H), 7.45–7.30 (m, 7H, Ar-H), 7.26 (t, *J* = 7.6 Hz, 2H, Ar-H), 7.19–7.05 (m, 3H, Ar-H), 6.97 (t, *J* = 8.1 Hz, 1H, Ar-H), 4.80 (t, *J* = 7.9 Hz, 1H, CHCH_2_), 4.46 (dd, *J* = 13.1, 8.1 Hz, 1H, CHCH_2_a), 3.94 (dd, *J* = 12.7, 7.8 Hz, 1H, CHCH_2_a), 2.19 (s, 3H, CH_3_). ^13^C NMR (101 MHz, DMSO-*d*_6_) *δ* 162.01, 143.53, 136.90, 136.60, 134.02, 133.23, 129.60, 129.49, 129.17, 128.98, 128.68, 128.20, 128.17, 127.39, 126.41, 124.06, 124.02, 121.72, 120.59, 119.34, 113.91, 113.17, 112.31, 112.02, 43.40, 41.74, 9.69. HRESI-MS *m/z* calcd. for [M+H]^+^ C_32_H_27_ClN_3_O: 504.1837, found: 504.1844.

##### 5-Chloro-3-ethyl-*N*-(2-phenyl-2-(2-phenyl-1H-indol-3-yl)ethyl)-1H-indole-2-carboxamide (**Vc**)

Yield % 78, mp 110-112 °C. ^1^H NMR (400 MHz, Chloroform-*d*) *δ* 9.15 (s, 1H, indole NH), 8.35 (s, 1H, 2-phenylindole NH), 7.72 (d, *J* = 8.0 Hz, 1H, Ar-H), 7.51–7.40 (m, 4H, Ar-H), 7.39–7.19 (m, 10H, Ar-H), 7.18–7.11 (m, 2H, Ar-H), 5.98 (s, 1H, amide NH), 4.79–4.75 (m, 1H, CHCH_2_), 4.72–4.60 (m, 1H, CHCH_2_a), 4.12–4.03 (m, 1H, CHCH_2_b), 2.30–2.15 (m, 2H, CH_2_CH_3_), 0.57 (t, *J* = 7.6 Hz, 3H, CH_2_CH_3_). ^13^C NMR (101 MHz, Chloroform-*d*) *δ* 161.56, 142.16, 137.59, 136.40, 133.23, 132.13, 128.92, 128.78, 128.73, 128.41, 128.39, 127.82, 127.66, 127.45, 126.69, 125.34, 124.78, 122.79, 120.59, 120.37, 119.17, 117.82, 112.71, 111.43, 110.69, 43.24, 42.28, 17.73, 14.42. HRESI-MS *m/z* calcd. for [M+H]^+^ C_33_H_29_ClN_3_O: 518.1994, found: 518.2000.

##### 5-Bromo-3-ethyl-*N*-(2-phenyl-2-(2-phenyl-1H-indol-3-yl)ethyl)-1H-indole-2-carboxamide (**Vd**)

Yield % 82, mp 130-132 °C. ^1^H NMR (400 MHz, Chloroform-*d*) *δ* 9.62 (s, 1H, indole NH), 8.54 (s, 1H, 2-phenylindole NH), 7.58 (d, *J* = 8.2 Hz, 1H, Ar-H), 7.45 (d, *J* = 1.8 Hz, 1H, Ar-H), 7.36–7.26 (m, 3H, Ar-H), 7.24–6.96 (m, 12H, Ar-H), 5.90 (t, *J* = 8.1 Hz, 1H, amide NH), 4.64 (dd, *J* = 11.0, 6.4 Hz, 1H, CHCH_2_), 4.57–4.50 (m, 1H, CHCH_2_a), 3.99–3.90 (m, 1H, CHCH_2_b), 2.17–1.98 (m, 2H, CH_2_CH_3_), 0.43 (t, *J* = 7.6 Hz, 3H, CH_2_CH_3_). ^13^C NMR (101 MHz, Chloroform-*d*) *δ* 161.98, 142.28, 137.73, 136.55, 133.91, 132.16, 129.33, 128.83, 128.43, 128.29, 127.86, 127.46, 127.41, 127.19, 126.78, 122.74, 122.28, 120.54, 120.34, 117.99, 113.50, 112.73, 111.63, 110.46, 43.47, 42.38, 17.78, 14.51. HRESI-MS *m/z* calcd. for [M+H]^+^ C_33_H_29_BrN_3_O: 562.1489, found: 562.1494.

##### 5-Chloro-3-(hydroxymethyl)-*N*-(2-phenyl-2-(2-phenyl-1H-indol-3-yl)ethyl)-1H-indole-2-carboxamide (**Ve**)

Yield % 74, mp 132–134 °C. ^1^H NMR (400 MHz, DMSO-*d*_6_) *δ* 11.67 (s, 1H, indole NH), 11.28 (s, 1H, 2-phenylindole NH), 8.90 (t, *J* = 6.3 Hz, 1H, amide NH), 7.69 (d, *J* = 1.9 Hz, 1H, Ar-H), 7.62 d, *J* = 7.8 Hz, 1H, Ar-H), 7.59–7.52 (m, 2H, Ar-H), 7.48–7.31 (m, 7H, Ar-H), 7.25 (t, *J* = 8.4 Hz, 2H, Ar-H), 7.15 (dd, *J* = 8.7, 2.1 Hz, 2H, Ar-H), 7.07 (t, *J* = 8.1 Hz, 1H, Ar-H), 6.95 (t, *J* = 8.1 Hz, 1H, Ar-H), 5.53 (t, *J* = 5.4 Hz, 1H, OH), 4.74 (t, *J* = 7.9 Hz, 1H, CHCH_2_), 4.50 (d, *J* = 5.5 Hz, 2H, CH_2_OH), 4.46–4.39 (m, 1H, CHCH_2_a), 4.08–4.00 (m, 1H, CHCH_2_b). ^13^C NMR (101 MHz, DMSO-*d*_6_) *δ* 161.57, 143.47, 136.84, 136.52, 133.70, 133.27, 131.50, 129.21, 128.98, 128.73, 128.17, 128.10, 128.04, 127.38, 126.46, 124.48, 123.92, 121.65, 120.53, 119.45, 119.30, 116.37, 114.17, 112.17, 111.96, 53.65, 43.63, 42.09. HRESI-MS *m/z* calcd. for [M+H]^+^ C_32_H_27_ClN_3_O_2_: 520.1786, found: 520.1793.

##### 5-Chloro-3-phenyl-*N*-(2-phenyl-2-(2-phenyl-1H-indol-3-yl)ethyl)-1H-indole-2-carboxamide (**Vf**)

Yield % 76, mp 131–133 °C. ^1^H NMR (400 MHz, Chloroform-*d*) *δ* 10.50 (s, 1H, indole NH), 8.25 (s, 1H, 2-phenylindole NH), 7.54–6.95 (m, 22H, Ar-H), 6.19 (s, 1H, amide NH), 4.69 (t, *J* = 9.9 Hz, 1H, CHCH_2_), 4.62–4.54 (m, 1H, CHCH_2_a), 3.95–3.86 (m, 1H, CHCH_2_b). ^13^C NMR (101 MHz, Chloroform-*d*) *δ* 161.52, 142.19, 137.03, 136.33, 133.61, 132.46, 132.39, 129.64, 129.15, 128.90, 128.61, 128.59, 128.52, 128.02, 127.97, 127.73, 127.38, 127.36, 126.48, 126.05, 125.04, 122.28, 120.45, 120.05, 119.87, 117.62, 113.38, 111.44, 111.27, 43.55, 41.72. HRESI-MS *m/z* calcd. for [M+H]^+^ C_37_H_29_ClN_3_O: 566.1994, found: 566.2000.

##### (*E*)-5-Chloro-3-(2-methoxyvinyl)-*N*-(2-phenyl-2-(2-phenyl-1H-indol-3-yl)ethyl)-1H-indole-2-carboxamide (**Vg**)

Yield % 78, mp 140–142 °C. ^1^H NMR (400 MHz, Chloroform-*d*) *δ* 9.79 (s, 1H, indole NH), 8.34 (s, 1H, 2-phenylindole NH), 7.62 (d, *J* = 8.1 Hz, 1H, Ar-H), 7.51 (d, *J* = 2.0 Hz, 1H, Ar-H), 7.48–7.39 (m, 3H, Ar-H), 7.37–7.05 (m, 12H, Ar-H), 6.59 (d, *J* = 13.0 Hz, 1H, CH=CHOCH_3_), 6.54 (t, *J* = 7.8 Hz, 1H, amide NH), 5.15 (d, *J* = 13.0 Hz, 1H, CH=CHOCH_3_), 4.78 (dd, *J* = 10.3, 6.3 Hz, 1H, CHCH_2_), 4.71–4.64 (m, 1H, CHCH_2_a), 4.13–4.01 (m, 1H, CHCH_2_b), 3.15 (s, 3H, OCH_3_). ^13^C NMR (101 MHz, Chloroform-*d*) *δ* 161.80, 153.12, 141.99, 137.54, 136.42, 133.67, 132.28, 128.69, 128.65, 128.63, 128.45, 128.13, 127.98, 127.83, 127.23, 126.55, 125.81, 124.91, 122.40, 120.57, 120.10, 119.89, 113.11, 111.40, 111.25, 111.22, 92.74, 56.27, 43.26, 42.00. HRESI-MS *m/z* calcd. for [M+H]^+^ C_34_H_29_ClN_3_O_2_: 546.1943, found: 546.1946.

##### 5-Chloro-3-(ethoxymethyl)-*N*-(2-phenyl-2-(2-phenyl-1H-indol-3-yl)ethyl)-1H-indole-2-carboxamide (**Vh**)

Yield % 80, mp 210–212 °C. ^1^H NMR (400 MHz, Chloroform-*d*) *δ* 10.36 (s, 1H, indole NH), 8.39 (t, *J* = 7.1 Hz, 1H, amide NH), 8.25 (s, 1H, 2-phenylindole NH), 7.64 (d, *J* = 8.0 Hz, 1H, Ar-H), 7.51–7.47 (m, 3H, Ar-H), 7.44–7.03 (m, 13H, Ar-H), 4.90 (dd, *J* = 9.7, 6.3 Hz, 1H, CHCH_2_), 4.74–4.63 (m, 1H, CHCH_2_a), 4.41 (d, *J* = 12.2 Hz, 1H, CH_2_aO), 4.25 (d, *J* = 12.2 Hz, 1H, CH_2_bO), 4.13–4.05 (m, 1H, CHCH_2_b), 3.08–2.94 (m, 1H, CH_2_aCH_3_), 2.85–2.79 (m, 1H, CH_2_bCH_3_), 0.66 (t, *J* = 7.0 Hz, 3H, CH_2_CH_3_). ^13^C NMR (101 MHz, Chloroform-*d*) *δ* 161.70, 142.32, 137.17, 136.42, 133.23, 132.51, 131.65, 128.64, 128.55, 128.50, 128.45, 127.99, 127.95, 127.55, 126.43, 125.87, 124.47, 122.26, 120.70, 119.97, 118.37, 113.49, 112.07, 111.47, 111.23, 64.96, 61.81, 43.92, 41.91, 14.40. HRESI-MS *m/z* calcd. for [M+H]^+^ C_34_H_31_ClN_3_O_2_: 548.2099, found: 548.2105.

##### 5-Chloro-3-ethyl-*N*-(2-phenyl-2-(2-phenyl-1H-indol-3-yl)ethyl)benzofuran-2-carboxamide (**Vi**)

Yield % 83, mp 225–227 °C. ^1^H NMR (400 MHz, DMSO-*d*_6_) *δ* 11.26 (s, 1H, 2-phenylindole NH), 8.54 (t, *J* = 7.0 Hz, 1H, amide NH), 7.84 (d, *J* = 2.1 Hz, 1H, Ar-H), 7.61–7.50 (m, 4H, Ar-H), 7.47–7.31 (m, 7H, Ar-H), 7.22 (t, *J* = 7.6 Hz, 2H, Ar-H), 7.13 -7.04 (m, 2H, Ar-H), 6.94 (t, *J* = 7.5 Hz, 1H, Ar-H), 4.85 (t, *J* = 7.8 Hz, 1H, CHCH_2_), 4.50–4.43 (m, 1H, CHCH_2_a), 3.85–3.79 (m, 1H, CHCH_2_b), 3.03–2.92 (m, CH_2_CH_3_), 1.12 (t, *J* = 7.5 Hz, 3H, CH_2_CH_3_). ^13^C NMR (101 MHz, DMSO-*d*_6_) *δ* 159.27, 151.54, 144.41, 143.43, 136.83, 136.38, 133.27, 130.28, 129.24, 128.92, 128.56, 128.17, 128.15, 128.13, 127.39, 127.32, 126.77, 126.28, 121.63, 121.03, 120.59, 119.21, 113.68, 112.60, 111.95, 42.78, 41.35, 16.72, 14.73. HRESI-MS *m/z* calcd. for [M+H]^+^ C_33_H_28_ClN_2_O_2_: 519.1834, found: 519.1830.

### 3.2. Biology

#### 3.2.1. Cell Viability Assay

To test the viability of the new compounds, the human mammary gland epithelial (MCF-10A) cell line was used [62,63]. Compounds **IV** and **Va**–**i** were incubated on MCF-10A cells for four days before being tested for viability using the MTT assay. See Supplementary Materials.

#### 3.2.2. Antiproliferative Assay

Using the MTT assay [59,65] and erlotinib as the reference drug, the antiproliferative activity of **Va**–**i** was assessed against four human cancer cell lines. See Supplementary Materials.

#### 3.2.3. EGFR Inhibitory Assay

The most potent antiproliferative derivatives (**Va**, **Ve**, **Vf**, **Vg**, and **Vh**) were also tested for EGFR inhibitory activity as a potential target for their antiproliferative activity [59,66]. See Supplementary Materials.

#### 3.2.4. BRAF^V600E^ Inhibitory Assay

Compounds **Va**, **Ve**, **Vf**, **Vg**, and **Vh** were further tested for BRAF^V600E^ inhibitory activity, and the results are shown in Table 2 as IC_50_ values [31,67]. See Supplementary Materials.

#### 3.2.5. VEGFR-2 Inhibitory Assay

The inhibitory activity of compounds **Va**, **Ve**, **Vf**, **Vg**, and **Vh** against VEGFR-2 was determined utilizing kinase-glo-luminescent kinase assays with sorafenib as the reference drug [63,72]. See Supplementary Materials.

### 3.3. Apoptotic Markers Assays

#### 3.3.1. Caspase-3 Assay

The most effective derivatives, compounds **Va**, **Ve**, **Vg**, and **Vh**, were evaluated as caspase-3 activators against a human epithelial cancer cell line (A-549) [73]. See Supplementary Materials.

#### 3.3.2. Caspase-8, Bax, and Bcl-2 Level Assays

Compounds **Va** and **Vg** were explored further for their impact on caspase-8, Bax, and Bacl-2 levels against a human epithelial cancer cell line (A-549) using doxorubicin as a reference [47]. See Supplementary Materials.

## 4. Conclusions

In this study, we presented the design, synthesis, and antiproliferative and apoptotic activities of a few novel indole-based derivatives (**IV** and **Va**–**i**). Various spectroscopic methods of analysis were used to confirm the structures of compounds **IV** and **Va**–**i**. Compounds **IV** and **Va**–**i** exhibited no cytotoxic effects in the cell viability test, and the cell viability for the compounds tested at 50 µM was greater than 89%. Compounds **IV** and **Va-i** demonstrated promising antiproliferative activity, with GI_50_ values ranging from 26 nM to 104 nM against the cancer cell lines tested compared to erlotinib’s 33 nM. The most potent antiproliferative derivatives **Va**, **Ve**, **Vf**, **Vg**, and **Vh** were tested for EGFR, BRAF^V600E^, and VEGFR-2 inhibitory activities as potential targets for their antiproliferative activity. Computational simulations confirmed the significance of the 5-chloroindole moiety in improving the fitting of the compound within the active sites of EGFR, Braf^V600E^, and VEGFR-2, highlighting the effect of the substituent at the third position of the indole scaffold on the binding of compound. Moreover, the amide linkage at the second position of the indole is significantly involved in H-bond interactions within the VEGFR-2 active site. In addition, the 2-phenyl indole scaffold bound considerably within the hydrophobic pocket of the binding sites. Our results showed good binding modes for compounds **Va**, **Vg**, and **Vh** within EGFR and Braf^V600E^. Also, docking results showed comparable fitting for compounds **Ve** and **Vg** within the active site of VEGFR-2. In silico ADME and pharmacokinetic prediction validated that the compounds have acceptable bioavailability and pharmacokinetic profiles. These findings revealed that compounds **Va**, **Ve**, **Vg**, and **Vh** displayed substantial antiproliferative effects and that they may operate as multi-kinase inhibitors, making them viable lead compounds for additional structural modifications.

## Figures and Tables

**Table 1:** IC_50_ of compounds **IV**, **Va**–**I**, and erlotinib.

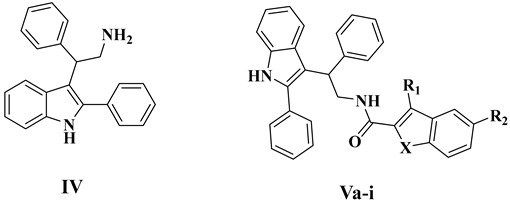									
Comp.	R_1_	R_2_	X	Cell Viability %	Antiproliferative Activity IC_50_ ± SEM (nM)				
					A-549	MCF-7	Panc-1	HT-29	Average (GI_50_)
**IV**	--	--	--	92	102 ± 10	106 ± 10	104 ± 10	104 ± 10	104
**Va**	H	Cl	NH	91	25 ± 2	28 ± 2	26 ± 2	26 ± 2	26
**Vb**	CH_3_	Cl	NH	88	58 ± 5	61 ± 6	58± 5	59 ± 5	59
**Vc**	CH_2_CH_3_	Cl	NH	91	54 ± 5	57 ± 5	56± 5	55 ± 5	56
**Vd**	CH_2_CH_3_	Br	NH	89	64 ± 6	68 ± 6	66 ± 6	66 ± 6	66
**Ve**	CH_2_OH	Cl	NH	90	42 ± 4	46 ± 4	44 ± 4	45 ± 4	44
**Vf**	Ph	Cl	NH	91	46 ± 4	49 ± 4	48 ± 4	48 ± 4	48
**Vg**	CH=CH-OCH_3_	Cl	NH	89	30 ± 2	33 ± 3	30 ± 2	30 ± 2	31
**Vh**	CH_2_OCH_2_CH_3_	Cl	NH	90	34 ± 3	38 ± 3	36 ± 3	38 ± 3	37
**Vi**	CH_2_CH_3_	Cl	O	89	86 ± 8	89 ± 8	85 ± 8	85 ± 8	86
**Erlotinib**	--	--	--	ND	30 ± 3	40 ± 3	30 ± 3	30 ± 3	33

--: Not Determined.

**Table 2:** IC_50_ of compounds **Va**, **Ve**, **Vf**, **Vg**, and **Vh** against EGFR and BRAF^V600E^.

Compd.	EGFR Inhibition IC_50_ ± SEM (nM)	BRAF^V600E^ Inhibition IC_50_ ± SEM (nM)	VEGFR-2 Inhibition IC_50_ (nM)
**Va**	71 ± 6	77 ± 6	2.15 ± 0.20
**Ve**	94 ± 7	97 ± 8	1.10 ± 0.08
**Vf**	103 ± 8	107 ± 9	2.50 ± 0.20
**Vg**	79 ± 6	83 ± 6	1.60 ± 0.10
**Vh**	85 ± 7	89 ± 7	3.25 ± 0.25
**Erlotinib**	80 ± 5	60 ± 5	--
**Sorafenib**	--	--	0.17 ± 0.01

--: Not Determined.

**Table 3:** Caspase-3, caspase-8, Bax, and Bcl-2 levels of compounds **Va**, **Ve**, **Vg**, and **Vh**.

Compd. No.	Caspase-3		Caspase-8		Bax		Bcl-2	
	Conc (pg/mL)	Fold Change	Conc (ng/mL)	Fold Change	Conc (pg/mL)	Fold Change	Conc (ng/mL)	Fold Reduction
**Va**	726± 6	11	3.50	35	410	45	0.75	7
**Ve**	462 ± 4	7	--	--	--	--	--	--
**Vg**	528 ± 5	8	2.20	22	320	35	0.85	6
**Vh**	460 ± 4	7	--	--	--	--	--	--
**Doxorubicin**	505 ± 4	7.5	1.80	18	280	31	0.90	6
**Control**	66	1	0.10	1	9	1	5	1

--: Not Determined.

**Table 4:** Ligand–protein complex interactions of the tested compounds **IV** and **Va**–**i** within the active site of EGFR.

Compd.	MOE Score kcal/mol	Hydrogen Bond Interactions	Hydrophobic Interactions	Other Interactions
**Erlotinib**	−10.70	Met769	Leu694, Leu820, Val702, Gly722, Thr766, Thr830	Leu694
**IV**	−7.79	Met769 Thr830	Leu820, Val702, Phe699, Asp831	Glu738 (ionic) Asp831 (ionic) Val702 (pi-H)
**Va**	−10.52	Asp831	Gly722, Thr766, Pro770, Glu780Leu694, Leu820, Val702	Gly772 (pi-H)
**Vb**	−8.89	Asp831 Asp776	Thr766, Pro770, Glu780, Leu694, Leu820, Val702, Gly722	Cys773 (pi-H)
**Vc**	−9.38	Asp831 Arg817	Leu694, Leu820, Val702, Gly722, Thr766, Pro770, Glu780, His781	--------------------
**Vd**	−9.35	Leu764 Asp831	Leu820, Val702, Gly722, Thr766, Leu694, Asp776, Glu780	Leu820 (pi-H)
**Ve**	−9.89	Asp831	Glu780, Leu694, Leu820, Val702, Gly722, Thr766, Asp776,	Leu694 (pi-H)
**Vf**	−9.90	Asp831 Asp776	Leu694, Leu820, Val702, Gly722, Thr766, Asp776, Glu780	Val702 (pi-H)
**Vg**	−10.05	Asp831	Leu694, Leu820, Val702, Gly722, Thr766, Asp776, Glu780	------------------
**Vh**	−10.13	Asp831 Arg817	Thr766, Asp776, Glu780, Leu694, Leu820, Val702, Gly722	Gly695, Val702 (pi-H)
**Vi**	−9.58	----------	Leu694, Leu820, Val702, Gly722, Thr766, Asp776, Glu780	Gly772 (pi-H)

**Table 5:** Ligand–protein complex interactions of the tested compounds **Va**, **Ve**, **Vf**, **Vg**, and **Vh** within the active site of BRAF^V600E^.

Compd.	MOE Score kcal/mol	Hydrogen Bond Interactions	Hydrophobic Interactions	Other Interactions
**Vemurafenib**	−11.78	Thr529 Gln530 Cys532 Asp594 Gly596	Trp531, Phe583, Cys532, Ile463, Thr592, val471, Lys483, Leu514	Lys483 (ionic)
**Va**	−7.97	Thr529	Phe583, Cys532, Thr592, val471, Lys483, Leu514	--------------------
**Ve**	−4.21	Leu505 Thr508 Lys483	Trp531, Phe583, Cys532, Ile463, Thr592, val471, Lys483, Leu514	--------------------
**Vf**	−4.30	----------	Trp531, Phe583, Cys532, Ile463, Thr592, val471, Lys483, Leu514	Val471 (pi-H) Leu514 (pi-H) Phe583 (pi-pi)
**Vg**	−7.32	----------	Trp531, Phe583, Cys532, Ile463, Thr592, val471, Lys483, Leu514	Val471 (pi-H)
**Vh**	−7.44	Asp594	Trp531, Phe583, Cys532, Ile463, Thr592, val471, Lys483, Leu514, Gly596	Val471 (pi-H) Ile527 (pi-H)

**Table 6:** Ligand–protein complex interactions of the tested compounds **Va**, **Ve**, **Vf**, **Vg**, and **Vh** within the active site of VEGFR-2.

Compd.	MOE Score kcal/mol	Hydrogen Bond Interactions	Hydrophobic Interactions	Pi-H Interactions
**Sorafenib**	−10.73	Cys919, Glu885	Val916, Leu889, Leu840, Asp1046, Cys1045 and Phe1047	Phe1047
**Va**	−9.61	Glu885, Asp1046	Leu889, Asp814, Asp1046, Glu885 and Leu886	-----------
**Ve**	−9.77	Glu885, Asp1046	Leu889, Asp814, Asp1046, Glu885 and Leu886, Cys1045 and His1026	Cys1045
**Vf**	−8.18	Glu885, Asp1046	Leu889, Asp814, Asp1046, Glu885 and Leu886, Cys1045 and Phe1047	Cys1045
**Vg**	−9.07	Cys1045, Asp1046	Leu889, Asp814, Asp1046, Glu885 and Leu886, Cys1045 and Phe1047	Cys1045
**Vh**	−9.09	Glu885, Asp1046, Cys1045	Leu889, Asp814, Asp1046, Glu885 and Leu886, Cys1045 and Phe1047	-------------

**Table 7:** Physicochemical and pharmacokinetic properties (Lipinski and Veber parameters) of compounds **IV** and **Va**–**i**.

Compd.	MW	^n^ROTB	HBA	HBD	Violations	MR	TPSA	Log P
**IV**	312	4	1	2	0	100	41.81	4.25
**Va**	490	7	1	3	1	147	60.68	6.22
**Vb**	504	7	1	3	2	152	60.68	6.61
**Vc**	518	8	1	3	2	157	60.68	6.79
**Vd**	563	8	1	3	2	160	60.68	6.91
**Ve**	520	8	2	4	2	154	80.91	5.72
**Vf**	566	8	1	3	2	173	60.68	7.59
**Vg**	546	9	2	3	2	164	69.91	6.54
**Vh**	548	10	2	3	2	163	69.91	6.5
**Vi**	519	8	2	2	2	155	58.03	7.12

**Table 8:** ADME properties of compounds **IV** and **Va**–**i**.

Compd.	GI Abs.	BBB	P-gp Substrate	CYP1A2 Inhibitor	CYP2C19 Inhibitor	CYP2C9 Inhibitor	CYP2D6 Inhibitor	CYP3A4 Inhibitor
**IV**	High	++	-	+	---	---	+	-
**Va**	Low	++	---	+	+	+	+	++
**Vb**	Low	++	---	+	+	+	+	+
**Vc**	Low	++	---	+	-	+	+	+
**Vd**	Low	++	---	+	+	+	-	+
**Ve**	Low	++	---	-	-	+	+	-
**Vf**	Low	++	---	+	-	+	+	-
**Vg**	Low	++	---	-	-	+	+	++
**Vh**	Low	+	---	-	---	+	+	+
**Vi**	Low	++	---	+	+	+	+	+

**Probability**: 0–0.1 (---); 0.1–0.3 (--); 0.3–0.5 (-); 0.5–0.7 (+); 0.7–0.9 (++); 0.9–1.0 (+++).

## Data Availability

Data are contained within the article and the supplementary materials.

## Supplementary Materials

The following supporting information can be downloaded at: https://www.mdpi.com/article/10.3390/ph16071039/s1, Figure S1. 3D binding mode of the redocked ligand (erlotinib) (white) into the active site of EGFRWT (PDB: 1M17) overlaid with the co-crystallized ligand (yellow), RMSD = 1.47 Å; Figure S2. 3D binding mode of the redocked ligand (Vemurafenib) (cyan) into the active site of BRAFV600E (PDB: 3OG7) overlaid with the co-crystallized ligand (purple), RMSD = 0.96 Å; Figure S3. 3D binding mode of the redocked ligand (sorafenib) (cyan) into the active site of VEGFR (PDB: 4ASD) overlaid with the co-crystallized ligand (purple), RMSD = 0.46 Å.

## References

[1] Blume-Jensen P., Hunter T. (2001). Oncogenic kinase signalling. Nature.

[2] Schwartz P.A., Murray B.W. (2011). Protein kinase biochemistry and drug discovery. Bioorg. Chem..

[3] Kannaiyan R., Mahadevan D. (2018). A comprehensive review of protein kinase inhibitors for cancer therapy. Expert Rev. Anticancer Ther..

[4] Bhullar K.S., Lagarón N.O., McGowan E.M., Parmar I., Jha A., Hubbard B.P., Rupasinghe H.V. (2018). Kinase-targeted cancer therapies: Progress, challenges and future directions. Mol. Cancer.

[5] Asati V., Mahapatra D.K., Bharti S.K. (2016). PI3K/Akt/mTOR and Ras/Raf/MEK/ERK signaling pathways inhibitors as anticancer agents: Structural and pharmacological perspectives. Eur. J. Med. Chem..

[6] Roskoski R. (2019). Properties of FDA-approved small molecule protein kinase inhibitors. Pharmacol. Res..

[7] Roskoski R. (2021). Properties of FDA-approved small molecule protein kinase inhibitors: A 2021 update. Pharmacol. Res..

[8] Xie Z., Yang X., Duan Y., Han J., Liao C. (2021). Small-molecule kinase inhibitors for the treatment of nononcologic diseases. J. Med. Chem..

[9] Kim G., Ko Y.T. (2020). Small molecule tyrosine kinase inhibitors in glioblastoma. Arch. Pharm. Res..

[10] Du J., Yan H., Xu Z., Yang B., He Q., Wang X., Luo P. (2021). Cutaneous toxicity of FDA-approved small-molecule kinase inhibitors. Expert Opin. Drug Metab. Toxicol..

[11] Ciardiello F., Tortora G. (2008). EGFR antagonists in cancer treatment. N. Engl. J. Med..

[12] Ivy S.P., Wick J.Y., Kaufman B.M. (2009). An overview of small-molecule inhibitors of VEGFR signaling. Nat. Rev. Clin. Oncol..

[13] Kaufman N.E., Dhingra S., Jois S.D., Vicente M.d.G.H. (2021). Molecular targeting of epidermal growth factor receptor (EGFR) and vascular endothelial growth factor receptor (VEGFR). Molecules.

[14] Rosell R., Moran T., Queralt C., Porta R., Cardenal F., Camps C., Majem M., Lopez-Vivanco G., Isla D., Provencio M. (2009). Screening for epidermal growth factor receptor mutations in lung cancer. N. Engl. J. Med..

[15] Hu W., Liu Y., Chen J. (2017). Concurrent gene alterations with EGFR mutation and treatment efficacy of EGFR-TKIs in Chinese patients with non-small cell lung cancer. Oncotarget.

[16] Nagy J.A., Dvorak A.M., Dvorak H.F. (2003). VEGF-A164/165 and PlGF: Roles in angiogenesis and arteriogenesis. Trends Cardiovasc. Med..

[17] Takahashi Y., Kitadai Y., Bucana C.D., Cleary K.R., Ellis L.M. (1995). Expression of vascular endothelial growth factor and its receptor, KDR, correlates with vascularity, metastasis, and proliferation of human colon cancer. Cancer Res..

[18] Poon R.T.-P., Fan S.-T., Wong J. (2001). Clinical implications of circulating angiogenic factors in cancer patients. J. Clin. Oncol..

[19] Shah A.A., Kamal M.A., Akhtar S. (2021). Tumor angiogenesis and VEGFR-2: Mechanism, pathways and current biological therapeutic interventions. Curr. Drug Metab..

[20] Azzoli C.G., Baker S., Temin S., Pao W., Aliff T., Brahmer J., Johnson D.H., Laskin J.L., Masters G., Milton D. (2009). American Society of Clinical Oncology clinical practice guideline update on chemotherapy for stage IV non–small-cell lung cancer. J. Clin. Oncol..

[21] Qin Y., Jian H., Tong X., Wu X., Wang F., Shao Y.W., Zhao X. (2020). Variability of EGFR exon 20 insertions in 24 468 Chinese lung cancer patients and their divergent responses to EGFR inhibitors. Mol. Oncol..

[22] Huang L., Huang Z., Bai Z., Xie R., Sun L., Lin K. (2012). Development and strategies of VEGFR-2/KDR inhibitors. Future Med. Chem..

[23] Enokida T., Tahara M. (2021). Management of VEGFR-Targeted TKI for thyroid Cancer. Cancers.

[24] Pitoia F., Jerkovich F. (2016). Selective use of sorafenib in the treatment of thyroid cancer. Drug Des. Dev. Ther..

[25] Wellbrock C., Karasarides M., Marais R. (2004). The RAF proteins take centre stage. Nat. Rev. Mol. Cell Biol..

[26] Ammar U.M., Abdel-Maksoud M.S., Oh C.-H. (2018). Recent advances of RAF (rapidly accelerated fibrosarcoma) inhibitors as anti-cancer agents. Eur. J. Med. Chem..

[27] Peyssonnaux C., Eychène A. (2001). The Raf/MEK/ERK pathway: New concepts of activation. Biol. Cell.

[28] Roskoski R. (2010). RAF protein-serine/threonine kinases: Structure and regulation. Biochem. Biophys. Res. Commun..

[29] Davies H., Bignell G.R., Cox C., Stephens P., Edkins S., Clegg S., Teague J., Woffendin H., Garnett M.J., Bottomley W. (2002). Mutations of the BRAF gene in human cancer. Nature.

[30] Samatar A.A., Poulikakos P.I. (2014). Targeting RAS–ERK signalling in cancer: Promises and challenges. Nat. Rev. Drug Discov..

[31] Al-Wahaibi L.H., Gouda A.M., Abou-Ghadir O.F., Salem O.I., Ali A.T., Farghaly H.S., Abdelrahman M.H., Trembleau L., Abdu-Allah H.H., Youssif B.G. (2020). Design and synthesis of novel 2, 3-dihydropyrazino [1, 2-a] indole-1, 4-dione derivatives as antiproliferative EGFR and BRAFV600E dual inhibitors. Bioorg. Chem..

[32] Mohassab A.M., Hassan H.A., Abdelhamid D., Gouda A.M., Youssif B.G., Tateishi H., Fujita M., Otsuka M., Abdel-Aziz M. (2021). Design and synthesis of novel quinoline/chalcone/1, 2, 4-triazole hybrids as potent antiproliferative agent targeting EGFR and BRAFV600E kinases. Bioorg. Chem..

[33] Bollag G., Tsai J., Zhang J., Zhang C., Ibrahim P., Nolop K., Hirth P. (2012). Vemurafenib: The first drug approved for BRAF-mutant cancer. Nat. Rev. Drug Discov..

[34] Khoja L., Hogg D. (2015). Dabrafenib in the treatment of metastatic or unresectable melanoma. Expert Rev. Anticancer Ther..

[35] Prahallad A., Sun C., Huang S., Di Nicolantonio F., Salazar R., Zecchin D., Beijersbergen R.L., Bardelli A., Bernards R. (2012). Unresponsiveness of colon cancer to BRAF (V600E) inhibition through feedback activation of EGFR. Nature.

[36] Wang Q., Hu W.-g., Song Q.-b., Wei J. (2014). BRAF V600E mutation as a predictive factor of anti-EGFR monoclonal antibodies therapeutic effects in metastatic colorectal cancer: A meta-analysis. Chin. Med. Sci. J..

[37] Caporali S., Amaro A., Levati L., Alvino E., Lacal P.M., Mastroeni S., Ruffini F., Bonmassar L., Antonini Cappellini G.C., Felli N. (2019). miR-126-3p down-regulation contributes to dabrafenib acquired resistance in melanoma by up-regulating ADAM9 and VEGF-A. J. Exp. Clin. Cancer Res..

[38] Lee C.-I., Liao C.-B., Chen C.-S., Cheng F.-Y., Chung Y.-H., Wang Y.-C., Ciou S.-Y., Hsueh W.-Y., Lo T.-H., Huang G.-R. (2021). Design and synthesis of 4-anilinoquinazolines as Raf kinase inhibitors. Part 1. Selective B-Raf/B-RafV600E and potent EGFR/VEGFR2 inhibitory 4-(3-hydroxyanilino)-6-(1H-1, 2, 3-triazol-4-yl) quinazolines. Bioorg. Chem..

[39] Connolly K., Brungs D., Szeto E., Epstein R. (2014). Anticancer activity of combination targeted therapy using cetuximab plus vemurafenib for refractory BRAFV600E-mutant metastatic colorectal carcinoma. Curr. Oncol..

[40] Grothey A., Fakih M., Tabernero J. (2021). Management of BRAF-mutant metastatic colorectal cancer: A review of treatment options and evidence-based guidelines. Ann. Oncol..

[41] Fondevila F., Méndez-Blanco C., Fernández-Palanca P., González-Gallego J., Mauriz J.L. (2019). Anti-tumoral activity of single and combined regorafenib treatments in preclinical models of liver and gastrointestinal cancers. Exp. Mol. Med..

[42] Comunanza V., Corà D., Orso F., Consonni F.M., Middonti E., Di Nicolantonio F., Buzdin A., Sica A., Medico E., Sangiolo D. (2017). VEGF blockade enhances the antitumor effect of BRAFV 600E inhibition. EMBO Mol. Med..

[43] Torres-Collado A.X., Knott J., Jazirehi A.R. (2018). Reversal of resistance in targeted therapy of metastatic melanoma: Lessons learned from Vemurafenib (BRAFV600E-specific inhibitor). Cancers.

[44] Cheng H., Chang Y., Zhang L., Luo J., Tu Z., Lu X., Zhang Q., Lu J., Ren X., Ding K. (2014). Identification and optimization of new dual inhibitors of B-Raf and epidermal growth factor receptor kinases for overcoming resistance against vemurafenib. J. Med. Chem..

[45] Prakash O., Kumar A., Kumar P. (2013). Anticancer potential of plants and natural products. Am J Pharmacol Sci.

[46] Sravanthi T., Manju S. (2016). Indoles—A promising scaffold for drug development. Eur. J. Pharm. Sci..

[47] Dhiman A., Sharma R., Singh R.K. (2022). Target-based anticancer indole derivatives and insight into structure‒activity relationship: A mechanistic review update (2018–2021). Acta Pharm. Sin. B.

[48] Youssif B.G., Abdelrahman M.H., Abdelazeem A.H., Ibrahim H.M., Salem O.I., Mohamed M.F., Treambleau L., Bukhari S.N.A. (2018). Design, synthesis, mechanistic and histopathological studies of small-molecules of novel indole-2-carboxamides and pyrazino [1, 2-a] indol-1 (2H)-ones as potential anticancer agents effecting the reactive oxygen species production. Eur. J. Med. Chem..

[49] Han Y., Dong W., Guo Q., Li X., Huang L. (2020). The importance of indole and azaindole scaffold in the development of antitumor agents. Eur. J. Med. Chem..

[50] Cragg G.M., Grothaus P.G., Newman D.J. (2009). Impact of natural products on developing new anti-cancer agents. Chem. Rev..

[51] Li W., Qi Y.Y., Wang Y.Y., Gan Y.Y., Shao L.H., Zhang L.Q., Tang Z.H., Zhu M., Tang S.Y., Wang Z.C. (2020). Design, synthesis, and biological evaluation of sorafenib derivatives containing indole (ketone) semicarbazide analogs as antitumor agents. J. Heterocycl. Chem..

[52] Singh P.K., Silakari O. (2018). Molecular dynamics guided development of indole based dual inhibitors of EGFR (T790M) and c-MET. Bioorg. Chem..

[53] Zhang H. (2016). Three generations of epidermal growth factor receptor tyrosine kinase inhibitors developed to revolutionize the therapy of lung cancer. Drug Des. Dev. Ther..

[54] Song J., Yoo J., Kwon A., Kim D., Nguyen H.K., Lee B.-Y., Suh W., Min K.H. (2015). Structure-activity relationship of indole-tethered pyrimidine derivatives that concurrently inhibit epidermal growth factor receptor and other angiokinases. PLoS ONE.

[55] Wu P., Choudhary A. (2018). Kinase Inhibitor Drugs. Success. Drug Discov..

[56] Ward R.A., Goldberg F.W. (2018). Kinase Drug Discovery: Modern Approaches.

[57] Al-Wahaibi L.H., Mohammed A.F., Abdelrahman M.H., Trembleau L., Youssif B.G. (2023). Design, Synthesis, and Antiproliferative Activity of New 5-Chloro-indole-2-carboxylate and Pyrrolo [3, 4-b] indol-3-one Derivatives as Potent Inhibitors of EGFRT790M/BRAFV600E Pathways. Molecules.

[58] Al-Wahaibi L.H., Mostafa Y.A., Abdelrahman M.H., El-Bahrawy A.H., Trembleau L., Youssif B.G. (2022). Synthesis and Biological Evaluation of Indole-2-Carboxamides with Potent Apoptotic Antiproliferative Activity as EGFR/CDK2 Dual Inhibitors. Pharmaceuticals.

[59] Gomaa H.A., Shaker M.E., Alzarea S.I., Hendawy O., Mohamed F.A., Gouda A.M., Ali A.T., Morcoss M.M., Abdelrahman M.H., Trembleau L. (2022). Optimization and SAR investigation of novel 2, 3-dihydropyrazino [1, 2-a] indole-1, 4-dione derivatives as EGFR and BRAFV600E dual inhibitors with potent antiproliferative and antioxidant activities. Bioorg. Chem..

[60] Mohamed F.A., Gomaa H.A., Hendawy O., Ali A.T., Farghaly H.S., Gouda A.M., Abdelazeem A.H., Abdelrahman M.H., Trembleau L., Youssif B.G. (2021). Design, synthesis, and biological evaluation of novel EGFR inhibitors containing 5-chloro-3-hydroxymethyl-indole-2-carboxamide scaffold with apoptotic antiproliferative activity. Bioorg. Chem..

[61] Mohamed F.A., Alakilli S.Y., El Azab E.F., Baawad F.A., Shaaban E.I.A., Alrub H.A., Hendawy O., Gomaa H.A., Bakr A.G., Abdelrahman M.H. (2023). Discovery of new 5-substituted-indole-2-carboxamides as dual epidermal growth factor receptor (EGFR)/cyclin dependent kinase-2 (CDK2) inhibitors with potent antiproliferative action. RSC Med. Chem..

[62] Gomaa H.A., El-Sherief H.A., Hussein S., Gouda A.M., Salem O.I., Alharbi K.S., Hayallah A.M., Youssif B.G. (2020). Novel 1, 2, 4-triazole derivatives as apoptotic inducers targeting p53: Synthesis and antiproliferative activity. Bioorg. Chem..

[63] Marzouk A.A., Abdel-Aziz S.A., Abdelrahman K.S., Wanas A.S., Gouda A.M., Youssif B.G., Abdel-Aziz M. (2020). Design and synthesis of new 1, 6-dihydropyrimidin-2-thio derivatives targeting VEGFR-2: Molecular docking and antiproliferative evaluation. Bioorg. Chem..

[64] Riss T.L., Moravec R.A., Niles A.L., Duellman S., Benink H.A., Worzella T.J., Minor L. (2016). Cell viability assays. Assay Guidance Manual.

[65] Mahmoud M.A., Mohammed A.F., Salem O.I., Gomaa H.A., Youssif B.G. (2022). New 1, 3, 4-oxadiazoles linked with the 1, 2, 3-triazole moiety as antiproliferative agents targeting the EGFR tyrosine kinase. Arch. Pharm..

[66] Abdel-Aziz S.A., Taher E.S., Lan P., Asaad G.F., Gomaa H.A., El-Koussi N.A., Youssif B.G. (2021). Design, synthesis, and biological evaluation of new pyrimidine-5-carbonitrile derivatives bearing 1, 3-thiazole moiety as novel anti-inflammatory EGFR inhibitors with cardiac safety profile. Bioorg. Chem..

[67] Abou-Zied H.A., Beshr E.A., Gomaa H.A., Mostafa Y.A., Youssif B.G., Hayallah A.M., Abdel-Aziz M. (2022). Discovery of new cyanopyridine/chalcone hybrids as dual inhibitors of EGFR/BRAFV600E with promising antiproliferative properties. Arch. Pharm..

[68] La D.S., Belzile J., Bready J.V., Coxon A., DeMelfi T., Doerr N., Estrada J., Flynn J.C., Flynn S.R., Graceffa R.F. (2008). Novel 2, 3-dihydro-1, 4-benzoxazines as potent and orally bioavailable inhibitors of tumor-driven angiogenesis. J. Med. Chem..

[69] Qiao L., Liang N., Zhang J., Xie J., Liu F., Xu D., Yu X., Tian Y. (2015). Advanced research on vasculogenic mimicry in cancer. J. Cell. Mol. Med..

[70] Okamoto K., Ikemori-Kawada M., Jestel A., von König K., Funahashi Y., Matsushima T., Tsuruoka A., Inoue A., Matsui J. (2015). Distinct binding mode of multikinase inhibitor lenvatinib revealed by biochemical characterization. ACS Med. Chem. Lett..

[71] Guo S., Colbert L.S., Fuller M., Zhang Y., Gonzalez-Perez R.R. (2010). Vascular endothelial growth factor receptor-2 in breast cancer. Biochim. Biophys. Acta Rev. Cancer.

[72] Mahmoud M.A., Mohammed A.F., Salem O.I., Rabea S.M., Youssif B.G. (2023). Design, synthesis, and antiproliferative properties of new 1, 2, 3-triazole-carboximidamide derivatives as dual EGFR/VEGFR-2 inhibitors. J. Mol. Struct..

[73] Abou-Zied H.A., Youssif B.G., Mohamed M.F., Hayallah A.M., Abdel-Aziz M. (2019). EGFR inhibitors and apoptotic inducers: Design, synthesis, anticancer activity and docking studies of novel xanthine derivatives carrying chalcone moiety as hybrid molecules. Bioorg. Chem..

[74] Qian S., Wei Z., Yang W., Huang J., Yang Y., Wang J. (2022). The role of BCL-2 family proteins in regulating apoptosis and cancer therapy. Front. Oncol..

[75] Singh P., Lim B. (2022). Targeting apoptosis in cancer. Curr. Oncol. Rep..

[76] Nouri Z., Fakhri S., Nouri K., Wallace C.E., Farzaei M.H., Bishayee A. (2020). Targeting multiple signaling pathways in cancer: The rutin therapeutic approach. Cancers.

[77] Martin S. (2014). Caspases: Executioners of apoptosis. Pathobiol. Hum. Dis..

[78] Choudhary G.S., Al-Harbi S., Almasan A. (2015). Caspase-3 activation is a critical determinant of genotoxic stress-induced apoptosis. Apoptosis Cancer: Methods and Protocol.

[79] Mazumder S., Plesca D., Almasan A. (2008). Caspase-3 activation is a critical determinant of genotoxic stress-induced apoptosis. Apoptosis Cancer: Methods and Protocol.

[80] Ibrahim T.S., Bokhtia R.M., Al-Mahmoudy A.M., Taher E.S., AlAwadh M.A., Elagawany M., Abdel-Aal E.H., Panda S., Gouda A.M., Asfour H.Z. (2020). Design, synthesis and biological evaluation of novel 5-((substituted quinolin-3-yl/1-naphthyl) methylene)-3-substituted imidazolidin-2, 4-dione as HIV-1 fusion inhibitors. Bioorg. Chem..

[81] Maier J.K., Labute P. (2014). Assessment of fully automated antibody homology modeling protocols in molecular operating environment. Proteins: Struct. Funct. Bioinform..

[82] Park J.H., Liu Y., Lemmon M.A., Radhakrishnan R. (2012). Erlotinib binds both inactive and active conformations of the EGFR tyrosine kinase domain. Biochem. J..

[83] Al-Wahaibi L.H., Mahmoud M.A., Mostafa Y.A., Raslan A.E., Youssif B.G. (2023). Novel piperine-carboximidamide hybrids: Design, synthesis, and antiproliferative activity via a multi-targeted inhibitory pathway. J. Enzym. Inhib. Med. Chem..

[84] Miller D.S., Voell S.A., Sosič I., Proj M., Rossanese O.W., Schnakenburg G., Gütschow M., Collins I., Steinebach C. (2022). Encoding BRAF inhibitor functions in protein degraders. RSC Med. Chem..

[85] Bakchi B., Krishna A.D., Sreecharan E., Ganesh V.B.J., Niharika M., Maharshi S., Puttagunta S.B., Sigalapalli D.K., Bhandare R.R., Shaik A.B. (2022). An overview on applications of SwissADME web tool in the design and development of anticancer, antitubercular and antimicrobial agents: A medicinal chemist’s perspective. J. Mol. Struct..

[86] Daina A., Michielin O., Zoete V. (2017). SwissADME: A free web tool to evaluate pharmacokinetics, drug-likeness and medicinal chemistry friendliness of small molecules. Sci. Rep..

[87] Dulsat J., López-Nieto B., Estrada-Tejedor R., Borrell J.I. (2023). Evaluation of Free Online ADMET Tools for Academic or Small Biotech Environments. Molecules.

[88] Daoud N.E.-H., Borah P., Deb P.K., Venugopala K.N., Hourani W., Alzweiri M., Bardaweel S.K., Tiwari V. (2021). ADMET profiling in drug discovery and development: Perspectives of in silico, in vitro and integrated approaches. Curr. Drug Metab..

