# Supplementary material for: Design, Synthesis, and Biological Evaluation of Indole-2-carboxamides as Potential Multi-Target Antiproliferative Agents
Source: Pharmaceuticals (Basel). 2023 Jul 22;16(7):1039. doi: 10.3390/ph16071039 (PMC10385579; doi:10.3390/ph16071039)
Supplement: Supplementary file 1 [file pharmaceuticals-16-01039-s001.zip › pharmaceuticals-2496320-supplementary.pdf]

Design, synthesis, and biological evaluation of indole-2-carboxamides as potential multi-target antiproliferative agents

Lamya H. Al-Wahaibi[1*], Anber F. Mohammed[2], Mostafa H. Abdelrahman[3], Laurent Trembleau[4*], Bahaa G. M. Youssif[2*]

[1]Department of Chemistry, College of Sciences, Princess Nourah bint Abdulrahman University, Riyadh, Saudi Arabia; [2]Pharmaceutical Organic Chemistry Department, Faculty of Pharmacy, Assiut University, Assiut, Egypt; [3]Pharmaceutical Organic Chemistry Department, Faculty of Pharmacy, Al-Azhar University, Assiut, Egypt; [4]School of Natural and Computing Sciences, University of Aberdeen, Aberdeen, United Kingdom.

**To whom correspondence should be addressed:*

Bahaa G. M. Youssif, Ph.D. Pharmaceutical Organic Chemistry Department, Faculty of Pharmacy, Assiut University, Assiut 71526, Egypt.

Tel.: (002)-01098294419

E-mail address: bgyoussif2@gmail.com, bahaa.youssif@pharm.aun.edu.eg

Lamya H. Al-Wahaibi: Department of Chemistry, College of Sciences, Princess Nourah bint Abdulrahman University lhalwahaibi@pnu.edu.sa

Laurent Trembleau, Ph.D. University of Aberdeen, Chemistry Department, The SyMBioSIS Group, Meston Building, Meston Walk, Aberdeen, AB24 3UE, United Kingdom.

Tel.: +44-(0)12242922

E-mail address: l.trembleau@abdn.ac.uk

III

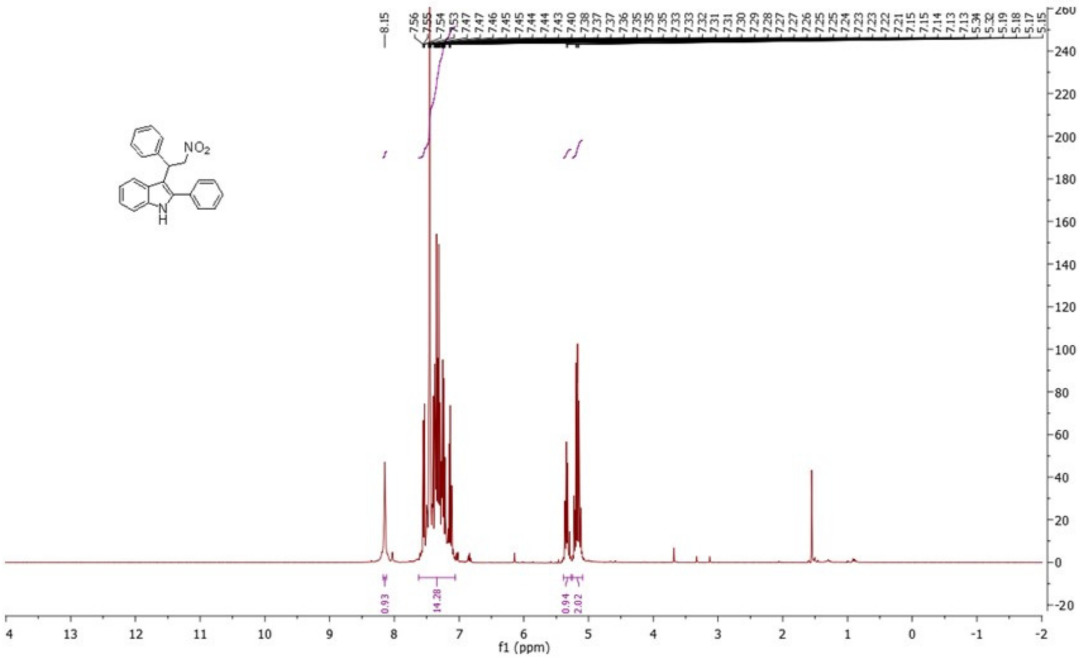

^{1}H NMR (400 MHz, Chloroform-*d*) δ 8.15 (s, 1H), 7.62 – 7.06 (m, 14H), 5.34 (dd, J = 9.2, 7.1, 1H), 5.25 – 5.09 (m, 2H).

Expanded Spectrum of III

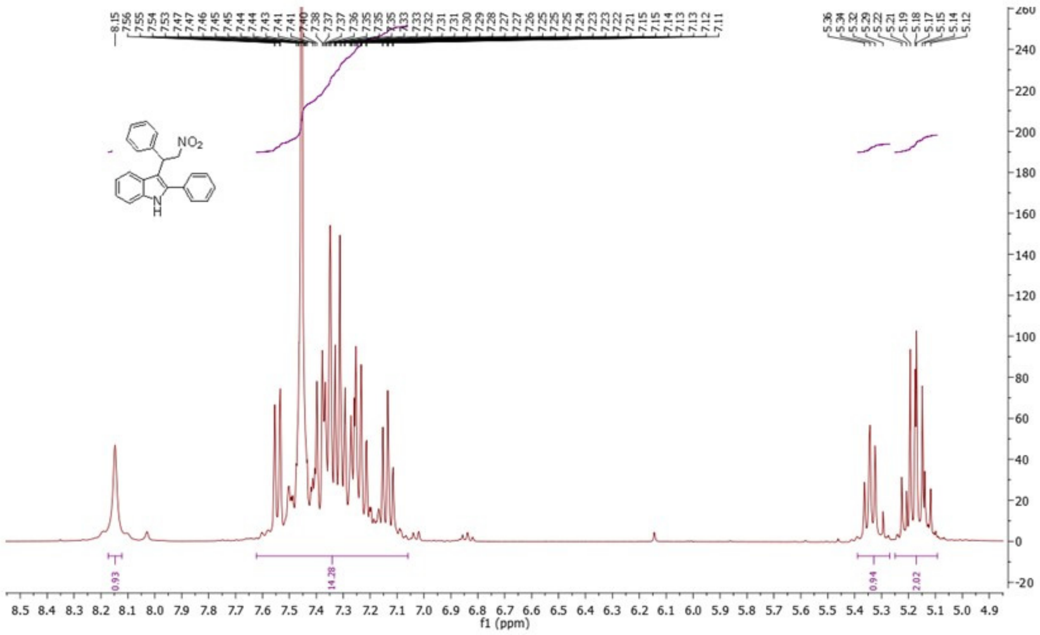

^{1}H NMR (400 MHz, Chloroform-*d*) δ 8.15 (s, 1H), 7.62 – 7.06 (m, 14H), 5.34 (dd, *J* = 9.2, 7.1, 1H), 5.25 – 5.09 (m, 2H).

III

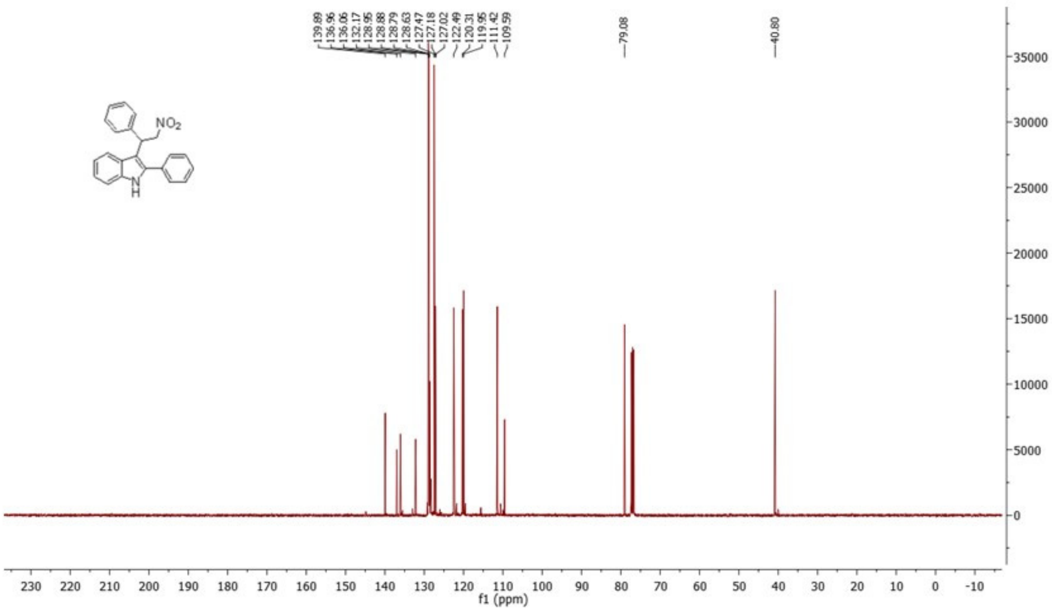

^{13}C NMR (101 MHz, cdcl$_3$) δ 139.89, 136.96, 136.06, 132.17, 128.95, 128.88, 128.79, 128.63, 127.47, 127.18, 127.02, 122.49, 120.31, 119.95, 111.42, 109.59, 79.08, 40.80.

Expanded Spectrum of III

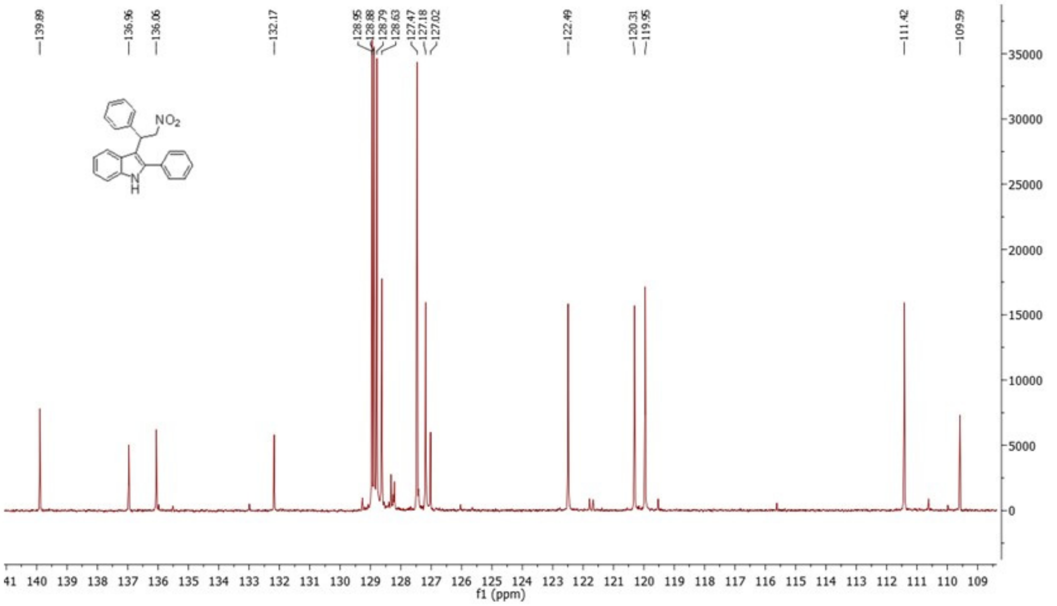

^{13}C NMR (101 MHz, cdcl$_3$) δ 139.89, 136.96, 136.06, 132.17, 128.95, 128.88, 128.79, 128.63, 127.47, 127.18, 127.02, 122.49, 120.31, 119.95, 111.42, 109.59, 79.08, 40.80.

Expanded Spectrum of Vi

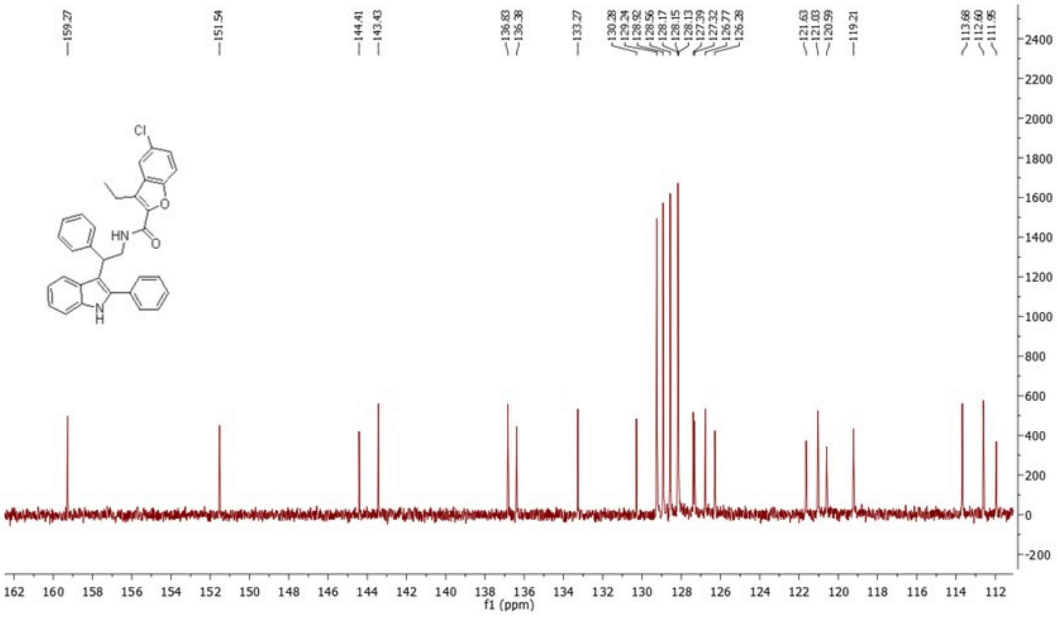

^{13}C NMR (101 MHz, dmso) δ 159.27, 151.54, 144.41, 143.43, 136.83, 136.38, 133.27, 130.28, 129.24, 128.92, 128.56, 128.17, 128.15, 128.13, 127.39, 127.32, 126.77, 126.28, 121.63, 121.03, 120.59, 119.21, 113.68, 112.60, 111.95, 42.78, 41.35, 16.72, 14.73.

Vi

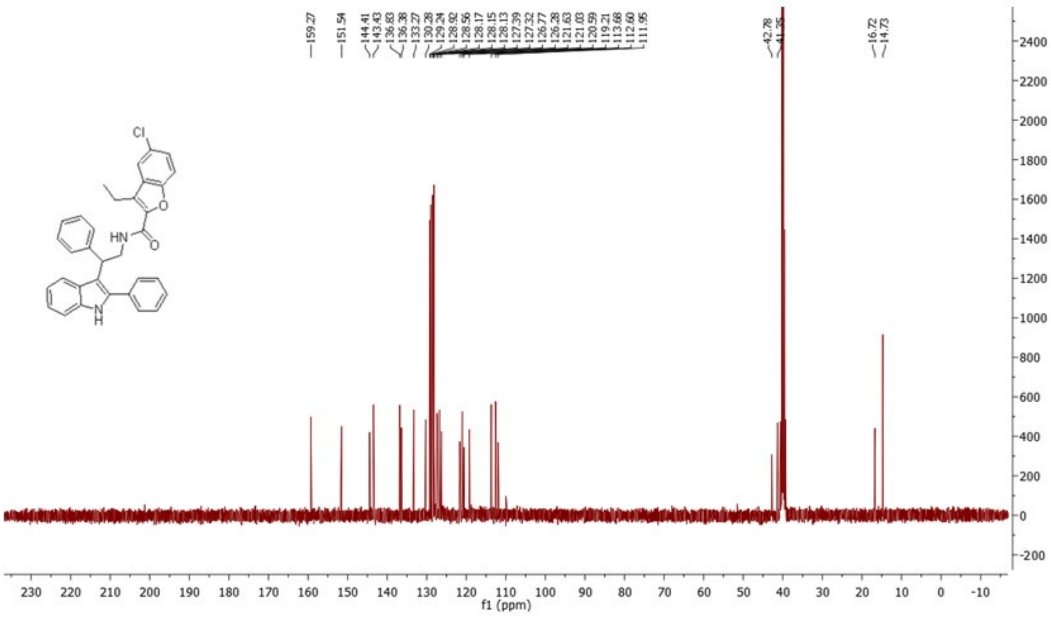

^{13}C NMR (101 MHz, dmso) δ 159.27, 151.54, 144.41, 143.43, 136.83, 136.38, 133.27, 130.28, 129.24, 128.92, 128.56, 128.17, 128.15, 128.13, 127.39, 127.32, 126.77, 126.28, 121.63, 121.03, 120.59, 119.21, 113.68, 112.60, 111.95, 42.78, 41.35, 16.72, 14.73.

Expanded Spectrum of Vi

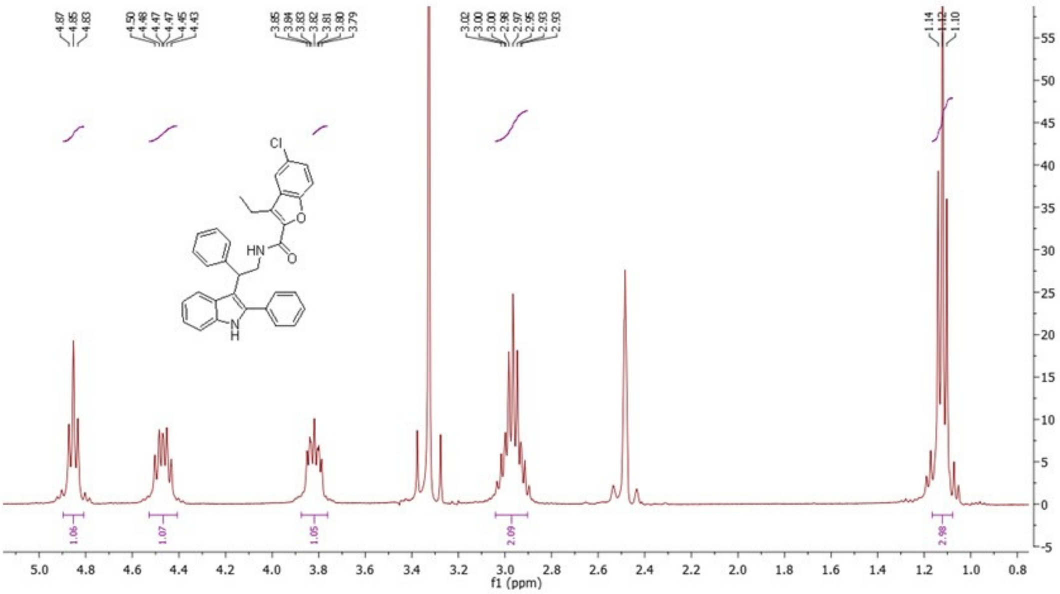

^{1}H NMR (400 MHz, DMSO-d_6) δ 11.26 (s, 1H), 8.54 (t, J = 7.0 Hz, 1H), 7.84 (d, J = 2.1 Hz, 1H), 7.61 – 7.50 (m, 4H), 7.47 – 7.31 (m, 7H), 7.22 (t, J = 7.6 Hz, 2H), 7.13 -7.04 (m, 2H), 6.94 (t, J = 7.5 Hz, 1H), 4.85 (t, J = 7.8 Hz, 1H), 4.50 - 4.43 (m, 1H), 3.85 - 3.79 (m, 1H), 3.03 - 2.92 (m, 2H), 1.12 (t, J = 7.5 Hz, 3H).

Expanded Spectrum of Vi

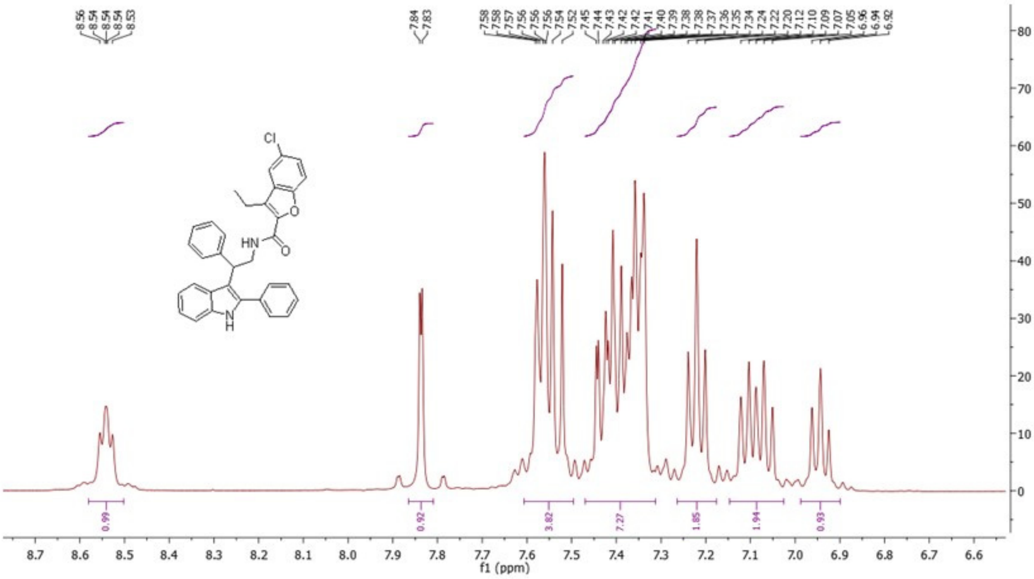

^{1}H NMR (400 MHz, DMSO-d_6) δ 11.26 (s, 1H), 8.54 (t, J = 7.0 Hz, 1H), 7.84 (d, J = 2.1 Hz, 1H), 7.61 – 7.50 (m, 4H), 7.47 – 7.31 (m, 7H), 7.22 (t, J = 7.6 Hz, 2H), 7.13 -7.04 (m, 2H), 6.94 (t, J = 7.5 Hz, 1H), 4.85 (t, J = 7.8 Hz, 1H), 4.50 - 4.43 (m, 1H), 3.85 - 3.79 (m, 1H), 3.03 - 2.92 (m, 2H), 1.12 (t, J = 7.5 Hz, 3H).

Vi

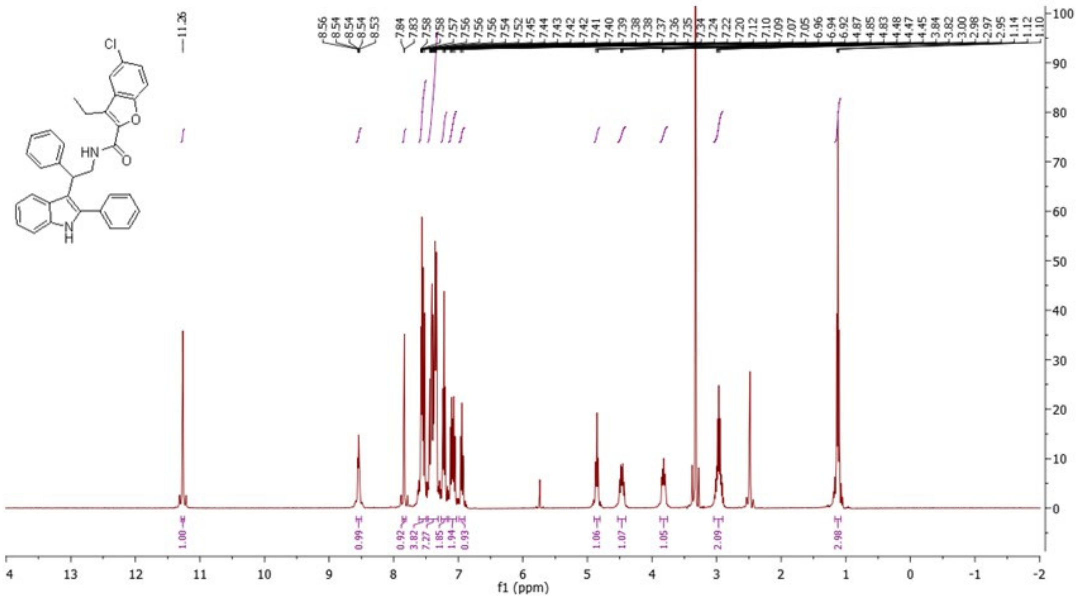

^{1}H NMR (400 MHz, DMSO-d_6) δ 11.26 (s, 1H), 8.54 (t, J = 7.0 Hz, 1H), 7.84 (d, J = 2.1 Hz, 1H), 7.61 – 7.50 (m, 4H), 7.47 – 7.31 (m, 7H), 7.22 (t, J = 7.6 Hz, 2H), 7.13 -7.04 (m, 2H), 6.94 (t, J = 7.5 Hz, 1H), 4.85 (t, J = 7.8 Hz, 1H), 4.50 - 4.43 (m, 1H), 3.85 - 3.79 (m, 1H), 3.03 - 2.92 (m, 2H), 1.12 (t, J = 7.5 Hz, 3H).

Expanded Spectrum of Vh

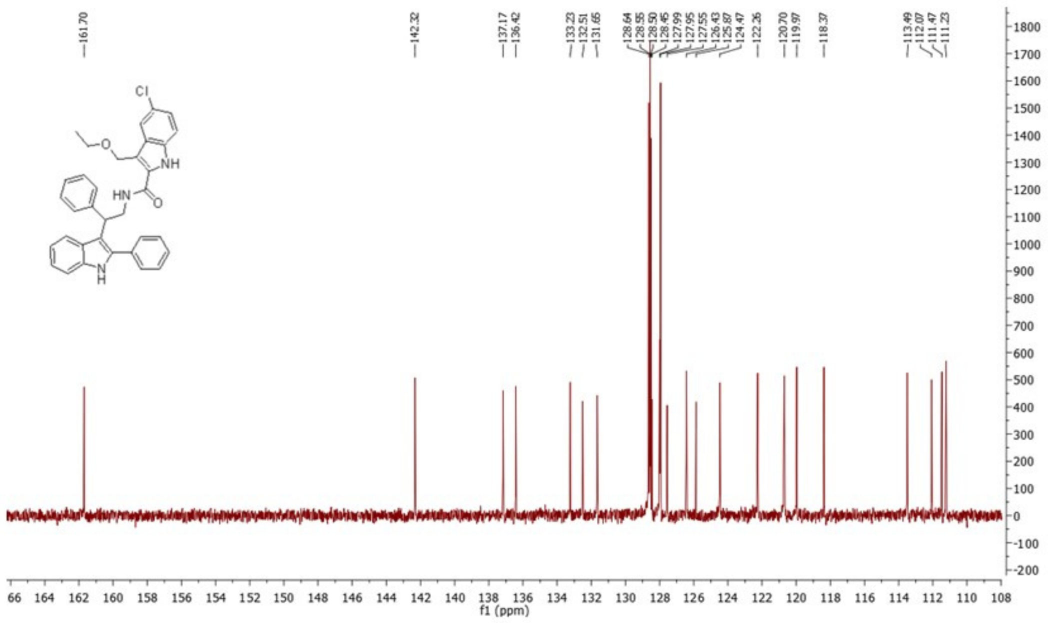

^{13}C NMR (101 MHz, cdcl$_3$) δ 161.70, 142.32, 137.17, 136.42, 133.23, 132.51, 131.65, 128.64, 128.55, 128.50, 128.45, 127.99, 127.95, 127.55, 126.43, 125.87, 124.47, 122.26, 120.70, 119.97, 118.37, 113.49, 112.07, 111.47, 111.23, 64.96, 61.81, 43.92, 41.91, 14.40.

Vh

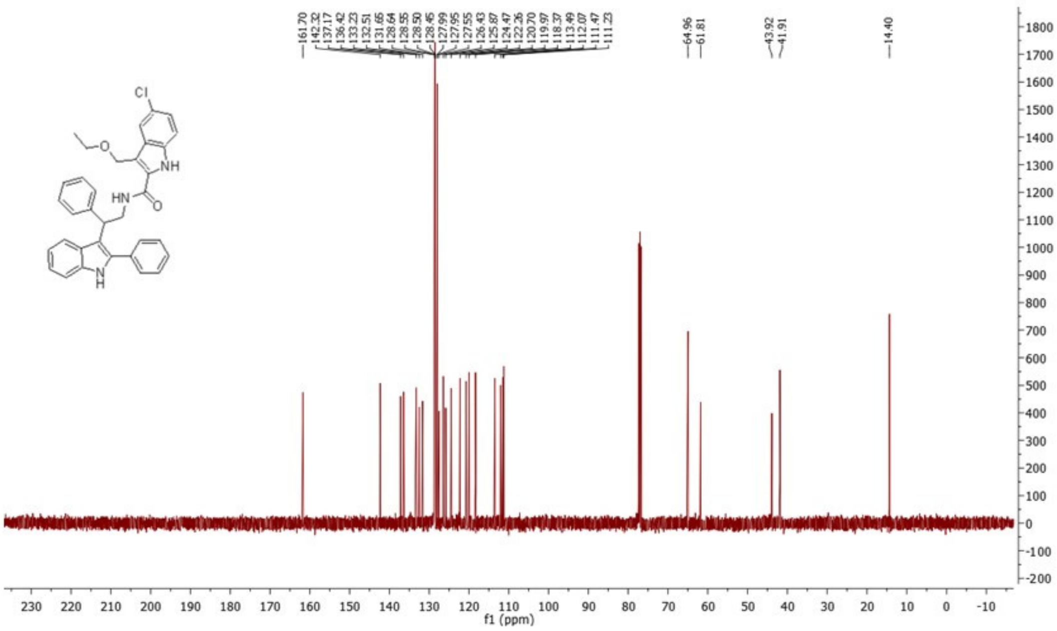

^{13}C NMR (101 MHz, cdcl$_3$) δ 161.70, 142.32, 137.17, 136.42, 133.23, 132.51, 131.65, 128.64, 128.55, 128.50, 128.45, 127.99, 127.95, 127.55, 126.43, 125.87, 124.47, 122.26, 120.70, 119.97, 118.37, 113.49, 112.07, 111.47, 111.23, 64.96, 61.81, 43.92, 41.91, 14.40.

Expanded Spectrum of Vh

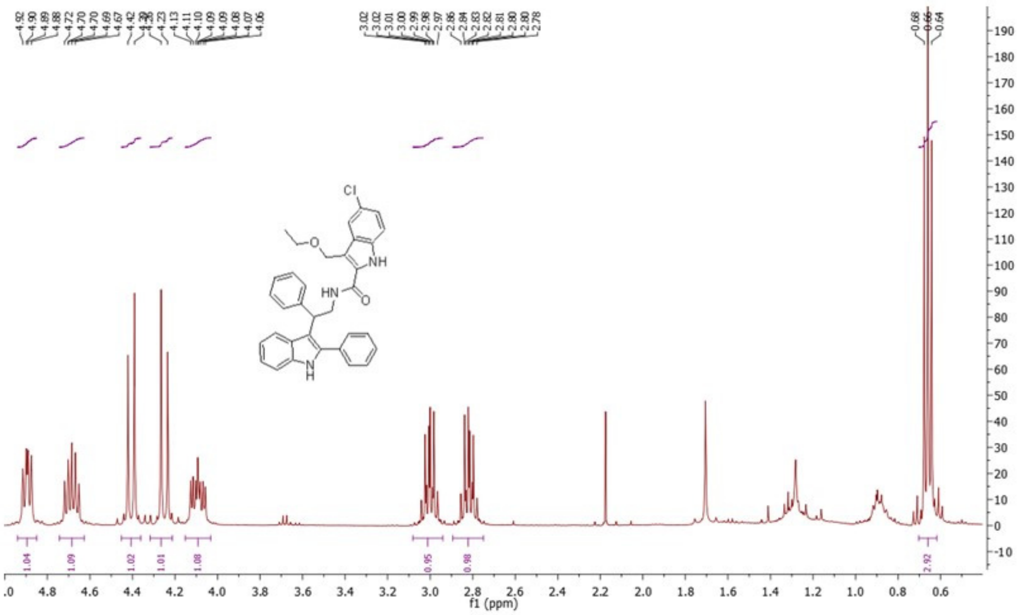

^{1}H NMR (400 MHz, Chloroform-*d*) δ 10.36 (s, 1H), 8.39 (t, *J* = 7.1 Hz, 1H), 8.25 (s, 1H), 7.64 (d, *J* = 8.0 Hz, 1H), 7.51 - 7.47 (m, 3H), 7.44 – 7.03 (m, 13H), 4.90 (dd, *J* = 9.7, 6.3 Hz, 1H), 4.74 – 4.63 (m, 1H), 4.41 (d, *J* = 12.2 Hz, 1H), 4.25 (d, *J* = 12.2 Hz, 1H), 4.13 - 4.05 (m, 1H), 3.08 – 2.94 (m, 1H), 2.85 - 2.79 (m, 1H), 0.66 (t, *J* = 7.0 Hz, 3H).

Expanded Spectrum of Vh

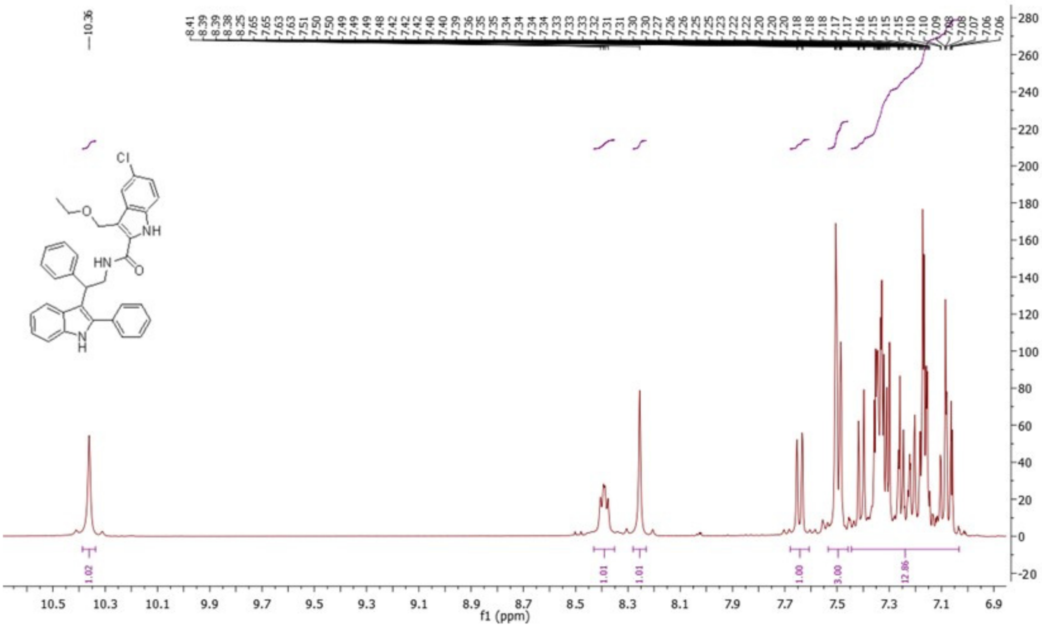

^{1}H NMR (400 MHz, Chloroform-*d*) δ 10.36 (s, 1H), 8.39 (t, *J* = 7.1 Hz, 1H), 8.25 (s, 1H), 7.64 (d, *J* = 8.0 Hz, 1H), 7.51 - 7.47 (m, 3H), 7.44 – 7.03 (m, 13H), 4.90 (dd, *J* = 9.7, 6.3 Hz, 1H), 4.74 – 4.63 (m, 1H), 4.41 (d, *J* = 12.2 Hz, 1H), 4.25 (d, *J* = 12.2 Hz, 1H), 4.13 - 4.05 (m, 1H), 3.08 – 2.94 (m, 1H), 2.85 - 2.79 (m, 1H), 0.66 (t, *J* = 7.0 Hz, 3H).

Vh

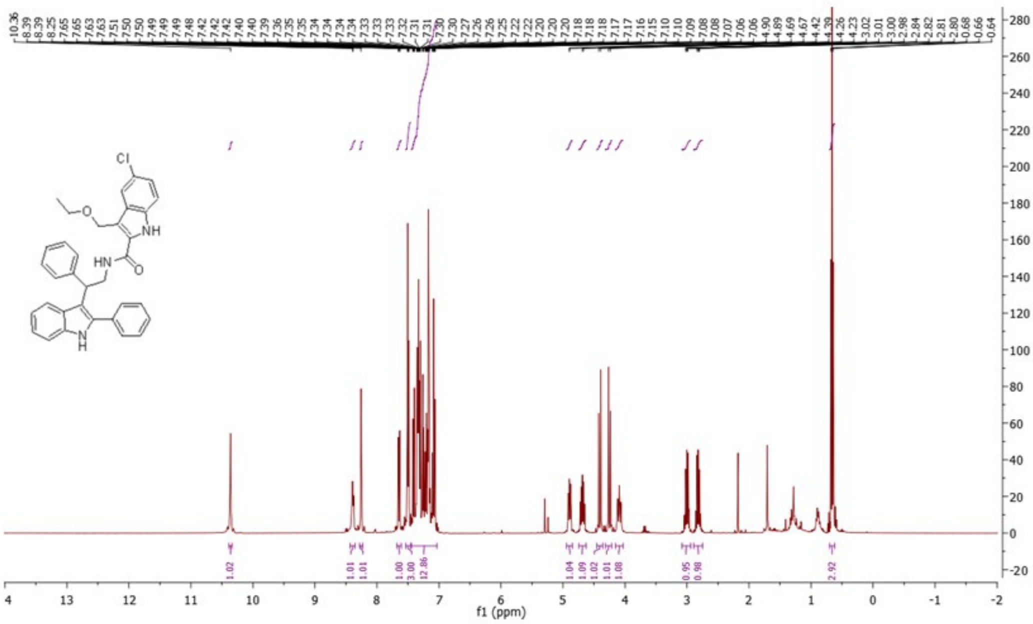

^{1}H NMR (400 MHz, Chloroform-*d*) δ 10.36 (s, 1H), 8.39 (t, *J* = 7.1 Hz, 1H), 8.25 (s, 1H), 7.64 (d, *J* = 8.0 Hz, 1H), 7.51 - 7.47 (m, 3H), 7.44 – 7.03 (m, 13H), 4.90 (dd, *J* = 9.7, 6.3 Hz, 1H), 4.74 – 4.63 (m, 1H), 4.41 (d, *J* = 12.2 Hz, 1H), 4.25 (d, *J* = 12.2 Hz, 1H), 4.13 - 4.05 (m, 1H), 3.08 – 2.94 (m, 1H), 2.85 - 2.79 (m, 1H), 0.66 (t, *J* = 7.0 Hz, 3H).

Expanded Spectrum of Vg

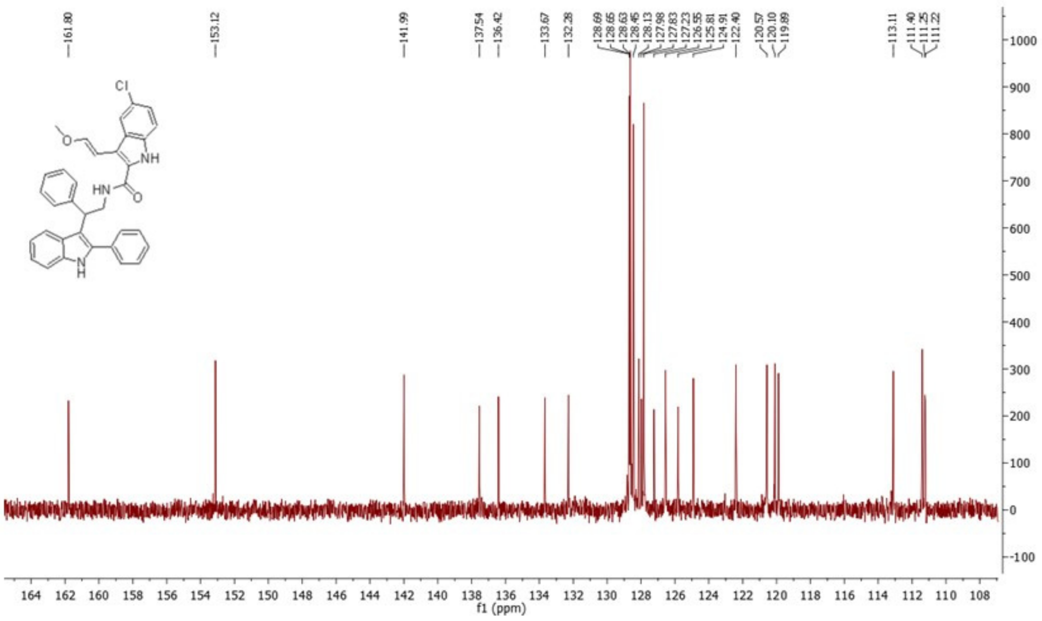

^{13}C NMR (101 MHz, cdcl$_3$) δ 161.80, 153.12, 141.99, 137.54, 136.42, 133.67, 132.28, 128.69, 128.65, 128.63, 128.45, 128.13, 127.98, 127.83, 127.23, 126.55, 125.81, 124.91, 122.40, 120.57, 120.10, 119.89, 113.11, 111.40, 111.25, 111.22, 92.74, 56.27, 43.26, 42.00.

Vg

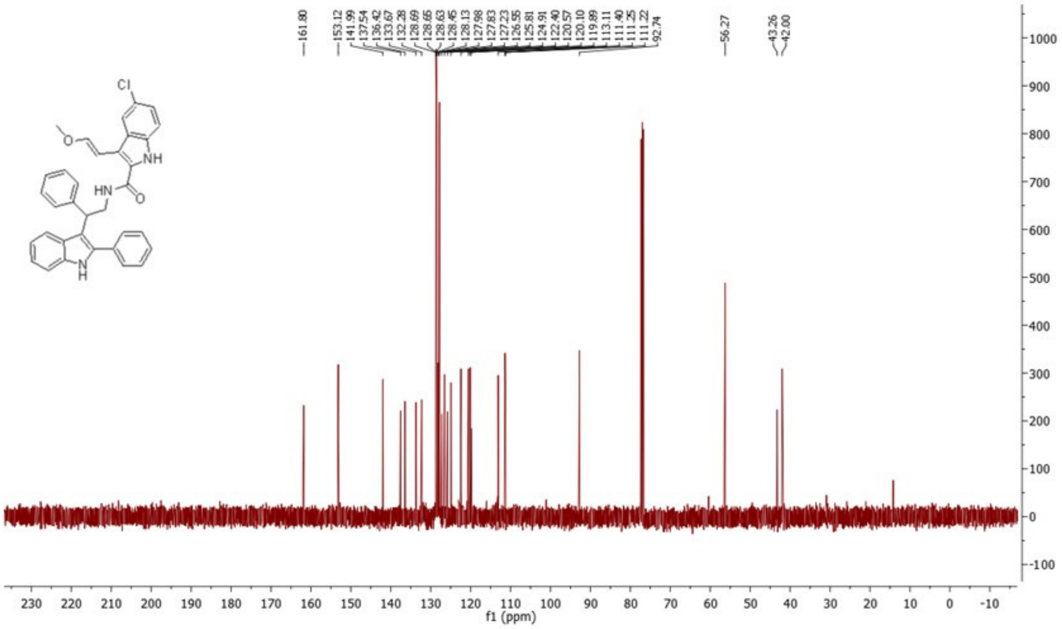

^{13}C NMR (101 MHz, cdcl$_3$) δ 161.80, 153.12, 141.99, 137.54, 136.42, 133.67, 132.28, 128.69, 128.65, 128.63, 128.45, 128.13, 127.98, 127.83, 127.23, 126.55, 125.81, 124.91, 122.40, 120.57, 120.10, 119.89, 113.11, 111.40, 111.25, 111.22, 92.74, 56.27, 43.26, 42.00.

Expanded Spectrum of Vg

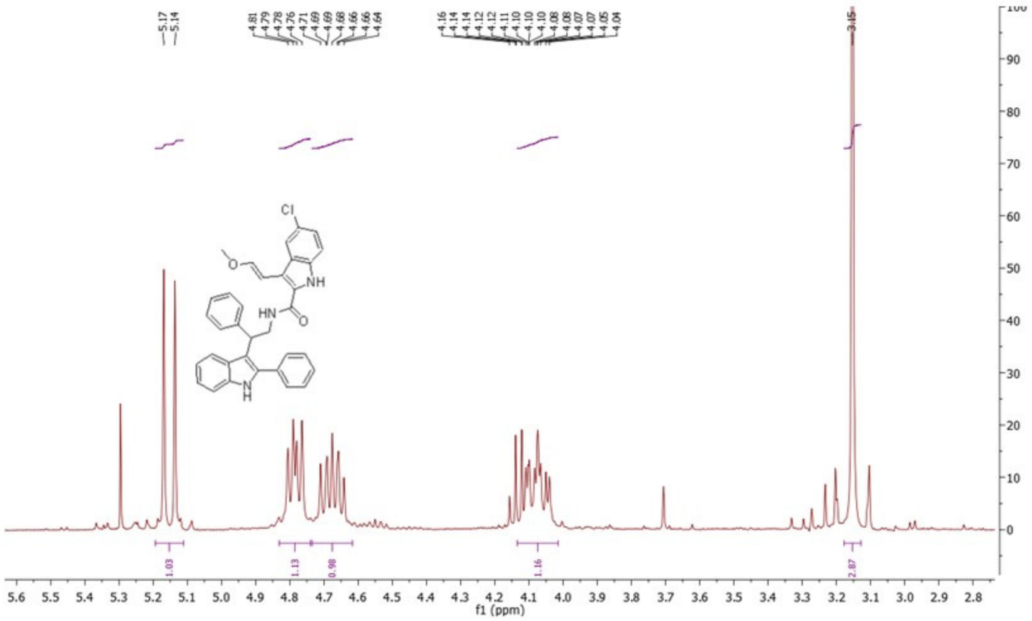

^{1}H NMR (400 MHz, Chloroform-*d*) δ 9.79 (s, 1H), 8.34 (s, 1H), 7.62 (d, *J* = 8.1 Hz, 1H), 7.51 (d, *J* = 2.0 Hz, 1H), 7.48 – 7.39 (m, 3H), 7.37 – 7.05 (m, 12H), 6.59 (d, *J* = 13.0 Hz, 1H), 6.54 (t, *J* = 7.8 Hz, 1H), 5.15 (d, *J* = 13.0 Hz, 1H), 4.78 (dd, *J* = 10.3, 6.3 Hz, 1H), 4.71 - 4.64 (m, 1H), 4.13 – 4.01 (m, 1H), 3.15 (s, 3H).

Expanded Spectrum of Vg

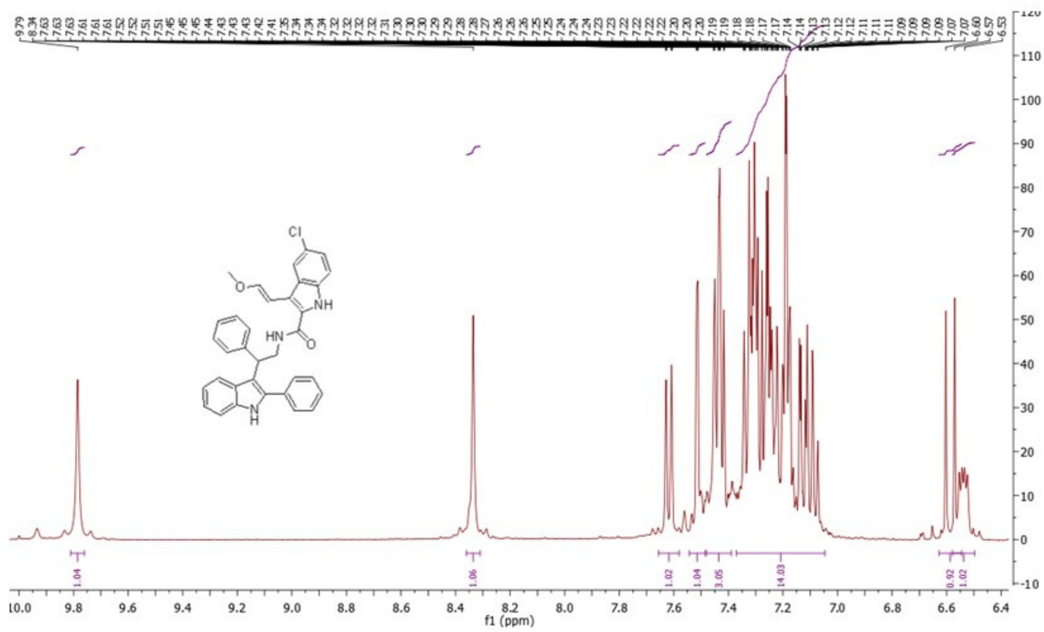

^{1}H NMR (400 MHz, Chloroform-*d*) δ 9.79 (s, 1H), 8.34 (s, 1H), 7.62 (d, *J* = 8.1 Hz, 1H), 7.51 (d, *J* = 2.0 Hz, 1H), 7.48 – 7.39 (m, 3H), 7.37 – 7.05 (m, 12H), 6.59 (d, *J* = 13.0 Hz, 1H), 6.54 (t, *J* = 7.8 Hz, 1H), 5.15 (d, *J* = 13.0 Hz, 1H), 4.78 (dd, *J* = 10.3, 6.3 Hz, 1H), 4.71 - 4.64 (m, 1H), 4.13 – 4.01 (m, 1H), 3.15 (s, 3H).

Expanded Spectrum of Vg

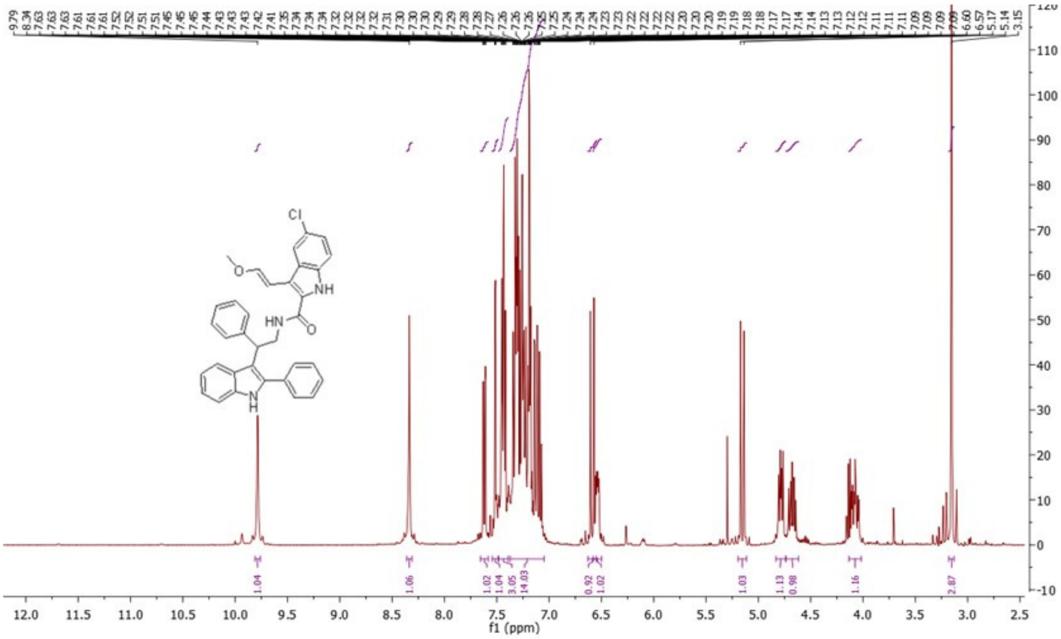

^{1}H NMR (400 MHz, Chloroform-*d*) δ 9.79 (s, 1H), 8.34 (s, 1H), 7.62 (d, *J* = 8.1 Hz, 1H), 7.51 (d, *J* = 2.0 Hz, 1H), 7.48 – 7.39 (m, 3H), 7.37 – 7.05 (m, 12H), 6.59 (d, *J* = 13.0 Hz, 1H), 6.54 (t, *J* = 7.8 Hz, 1H), 5.15 (d, *J* = 13.0 Hz, 1H), 4.78 (dd, *J* = 10.3, 6.3 Hz, 1H), 4.71 - 4.64 (m, 1H), 4.13 – 4.01 (m, 1H), 3.15 (s, 3H).

Vg

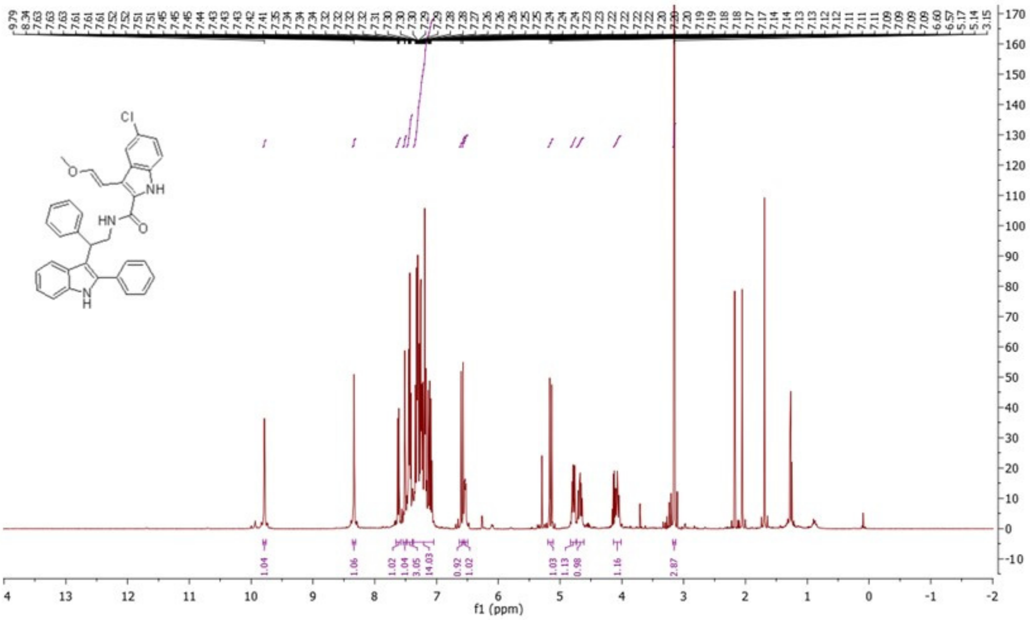

^{1}H NMR (400 MHz, Chloroform-*d*) δ 9.79 (s, 1H), 8.34 (s, 1H), 7.62 (d, *J* = 8.1 Hz, 1H), 7.51 (d, *J* = 2.0 Hz, 1H), 7.48 – 7.39 (m, 3H), 7.37 – 7.05 (m, 12H), 6.59 (d, *J* = 13.0 Hz, 1H), 6.54 (t, *J* = 7.8 Hz, 1H), 5.15 (d, *J* = 13.0 Hz, 1H), 4.78 (dd, *J* = 10.3, 6.3 Hz, 1H), 4.71 - 4.64 (m, 1H), 4.13 – 4.01 (m, 1H), 3.15 (s, 3H).

Expanded Spectrum of Vf

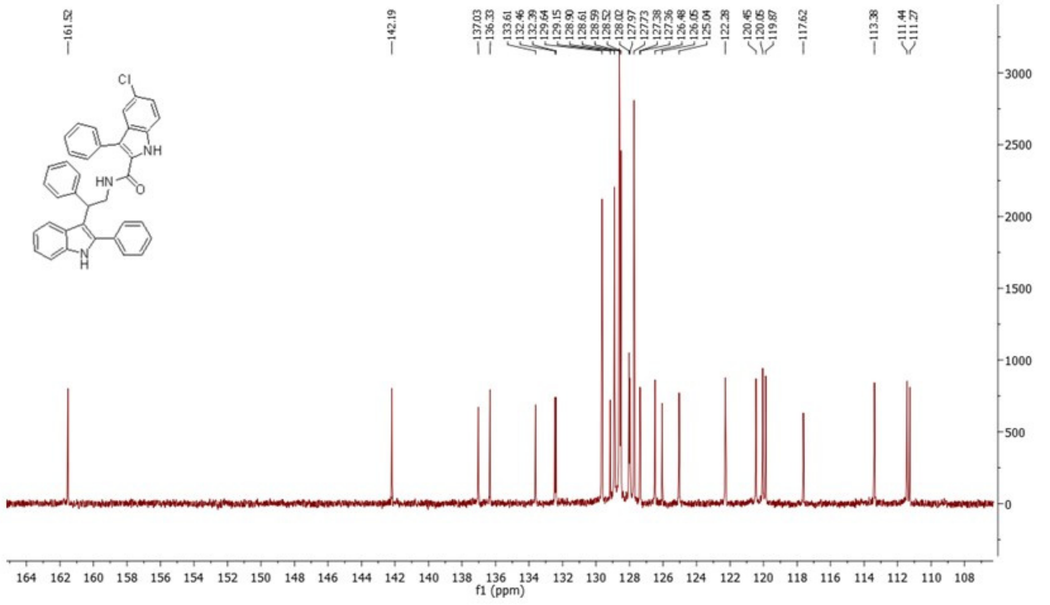

^{13}C NMR (101 MHz, cdcl$_3$) δ 161.52, 142.19, 137.03, 136.33, 133.61, 132.46, 132.39, 129.64, 129.15, 128.90, 128.61, 128.59, 128.52, 128.02, 127.97, 127.73, 127.38, 127.36, 126.48, 126.05, 125.04, 122.28, 120.45, 120.05, 119.87, 117.62, 113.38, 111.44, 111.27, 43.55, 41.72.

Vf

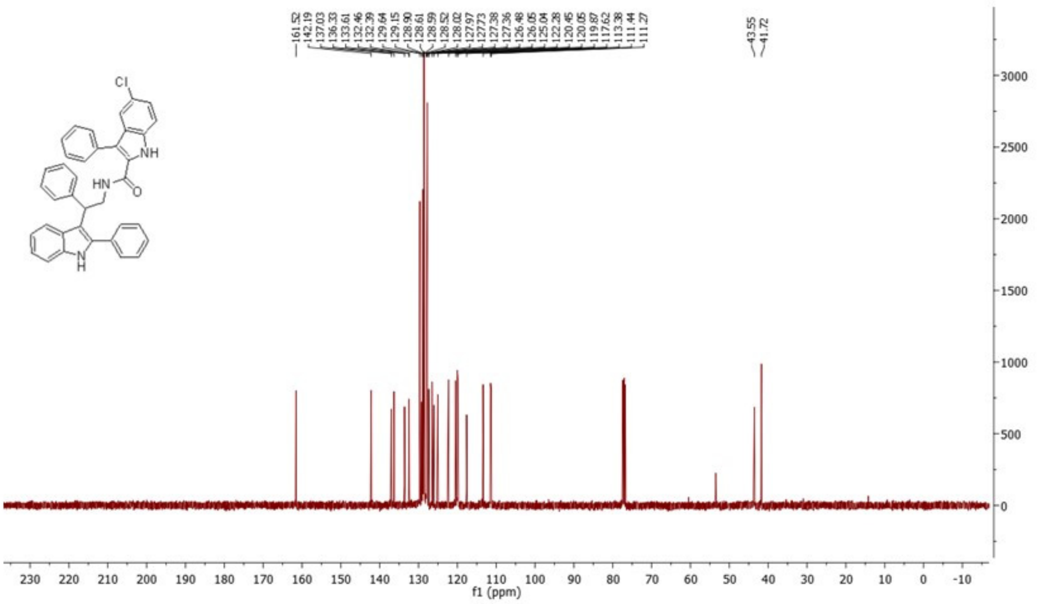

^{13}C NMR (101 MHz, cdcl$_3$) δ 161.52, 142.19, 137.03, 136.33, 133.61, 132.46, 132.39, 129.64, 129.15, 128.90, 128.61, 128.59, 128.52, 128.02, 127.97, 127.73, 127.38, 127.36, 126.48, 126.05, 125.04, 122.28, 120.45, 120.05, 119.87, 117.62, 113.38, 111.44, 111.27, 43.55, 41.72.

Expanded Spectrum of Vf

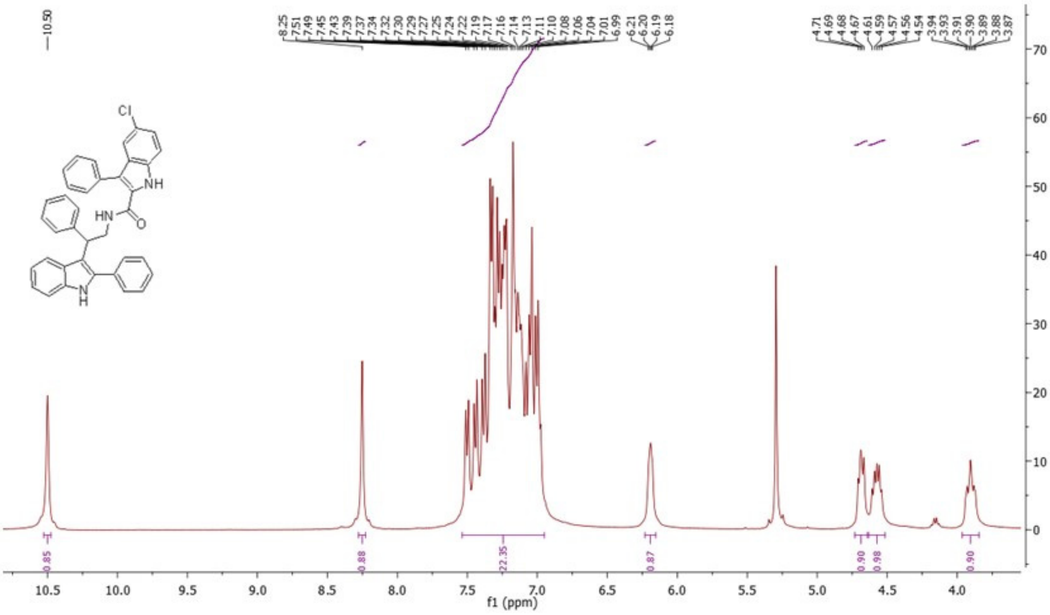

^{1}H NMR (400 MHz, Chloroform-*d*) δ 10.50 (s, 1H), 8.25 (s, 1H), 7.54 – 6.95 (m, 22H), 6.19 (s, 1H), 4.69 (t, *J* = 9.9 Hz, 1H), 4.62 - 4.54 (m, 1H), 3.95 - 3.86 (m, 1H).

Vf

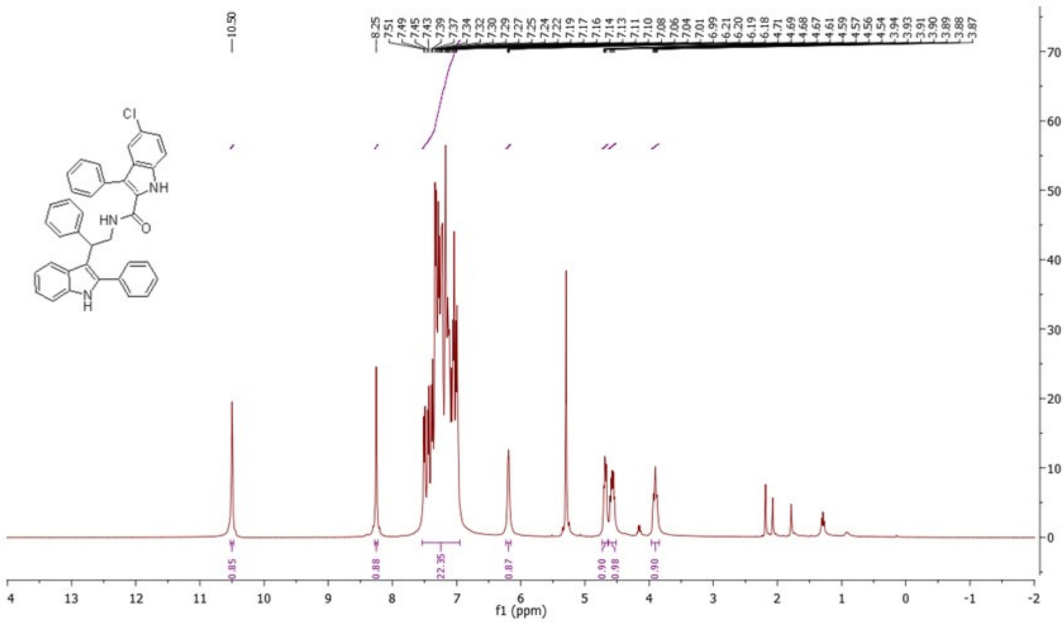

^{1}H NMR (400 MHz, Chloroform-*d*) δ 10.50 (s, 1H), 8.25 (s, 1H), 7.54 – 6.95 (m, 22H), 6.19 (s, 1H), 4.69 (t, *J* = 9.9 Hz, 1H), 4.62 - 4.54 (m, 1H), 3.95 - 3.86 (m, 1H).

Expanded Spectrum of Ve

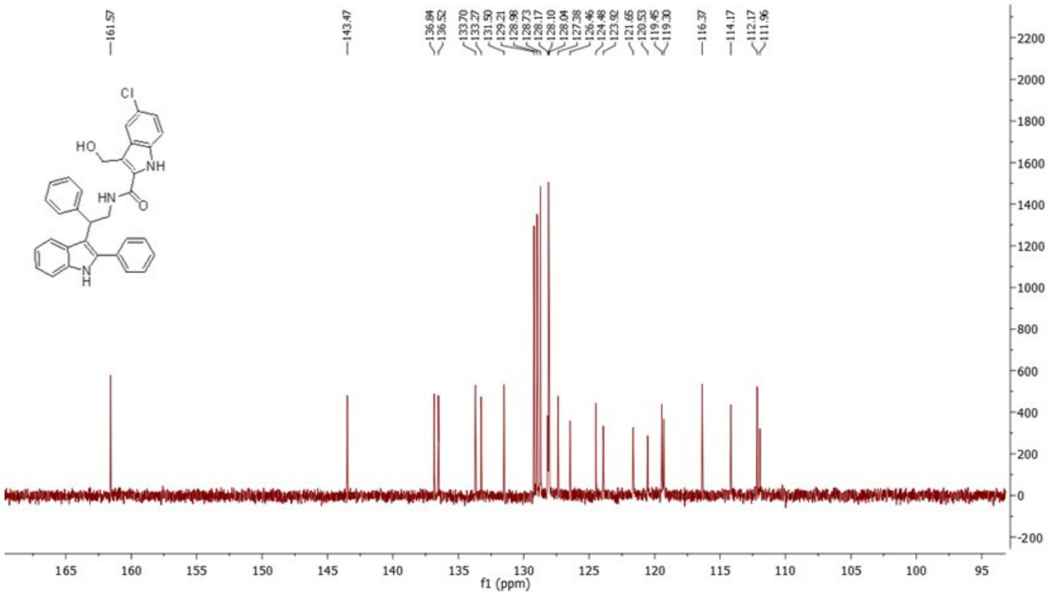

^{13}C NMR (101 MHz, dmso) δ 161.57, 143.47, 136.84, 136.52, 133.70, 133.27, 131.50, 129.21, 128.98, 128.73, 128.17, 128.10, 128.04, 127.38, 126.46, 124.48, 123.92, 121.65, 120.53, 119.45, 119.30, 116.37, 114.17, 112.17, 111.96, 53.65, 43.63, 42.09.

Ve

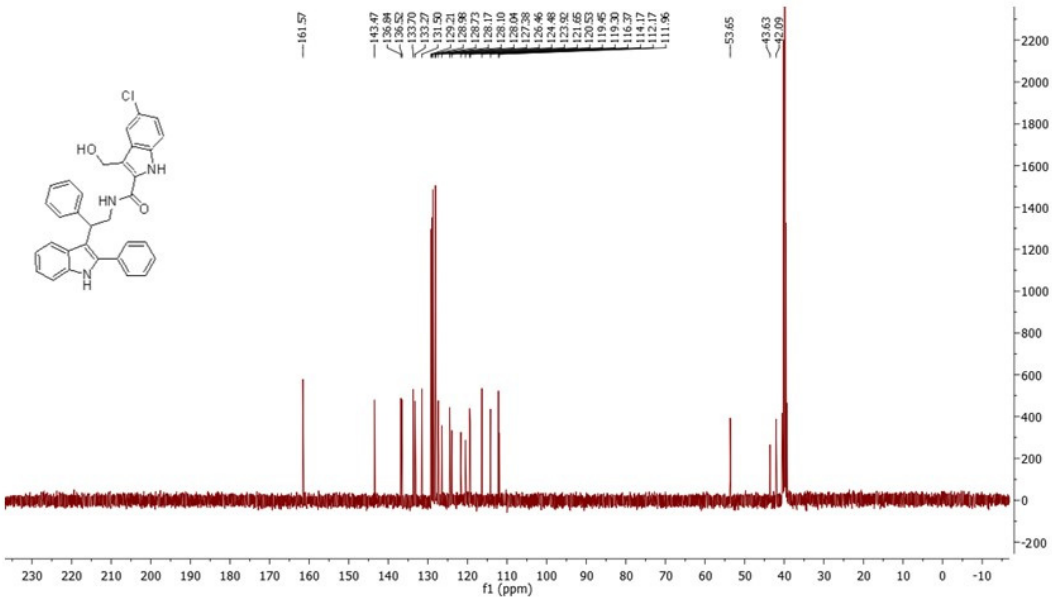

^{13}C NMR (101 MHz, dmso) δ 161.57, 143.47, 136.84, 136.52, 133.70, 133.27, 131.50, 129.21, 128.98, 128.73, 128.17, 128.10, 128.04, 127.38, 126.46, 124.48, 123.92, 121.65, 120.53, 119.45, 119.30, 116.37, 114.17, 112.17, 111.96, 53.65, 43.63, 42.09.

Expanded Spectrum of Ve

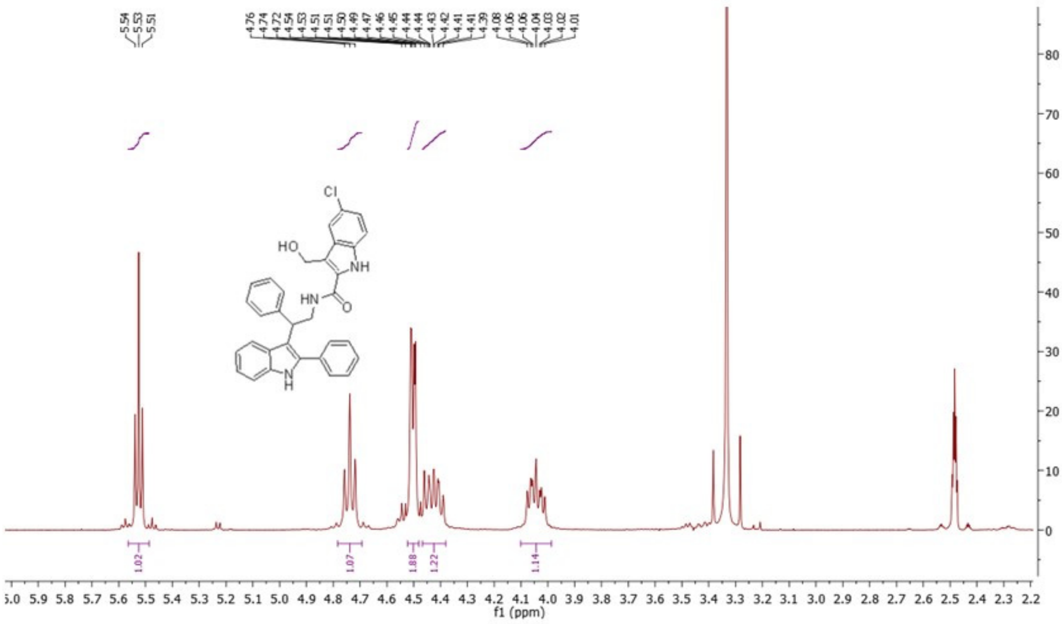

^{1}H NMR (400 MHz, DMSO-d_6) δ 11.67 (s, 1H), 11.28 (s, 1H), 8.90 (t, J = 6.3 Hz, 1H), 7.69 (d, J = 1.9 Hz, 1H), 7.62 d, J = 7.8 Hz, 1H), 7.59 – 7.52 (m, 2H), 7.48 – 7.31 (m, 7H), 7.25 (t, J = 8.4 Hz, 2H), 7.15 (dd, J = 8.7, 2.1 Hz, 2H), 7.07 (t, J = 8.1 Hz, 1H), 6.95 (t, J = 8.1 Hz, 1H), 5.53 (t, J = 5.4 Hz, 1H), 4.74 (t, J = 7.9 Hz, 1H), 4.50 (d, J = 5.5 Hz, 2H), 4.46 – 4.39 (m, 1H), 4.08 - 4.00 (m, 1H).

Expanded Spectrum of Ve

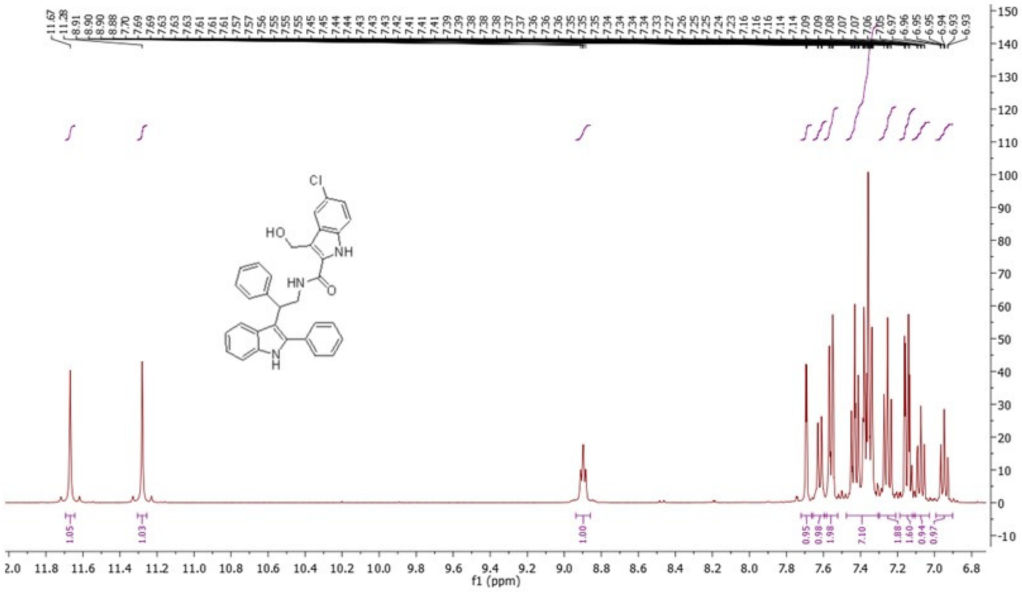

^{1}H NMR (400 MHz, DMSO-d_6) δ 11.67 (s, 1H), 11.28 (s, 1H), 8.90 (t, J = 6.3 Hz, 1H), 7.69 (d, J = 1.9 Hz, 1H), 7.62 d, J = 7.8 Hz, 1H), 7.59 – 7.52 (m, 2H), 7.48 – 7.31 (m, 7H), 7.25 (t, J = 8.4 Hz, 2H), 7.15 (dd, J = 8.7, 2.1 Hz, 2H), 7.07 (t, J = 8.1 Hz, 1H), 6.95 (t, J = 8.1 Hz, 1H), 5.53 (t, J = 5.4 Hz, 1H), 4.74 (t, J = 7.9 Hz, 1H), 4.50 (d, J = 5.5 Hz, 2H), 4.46 – 4.39 (m, 1H), 4.08 - 4.00 (m, 1H).

Ve

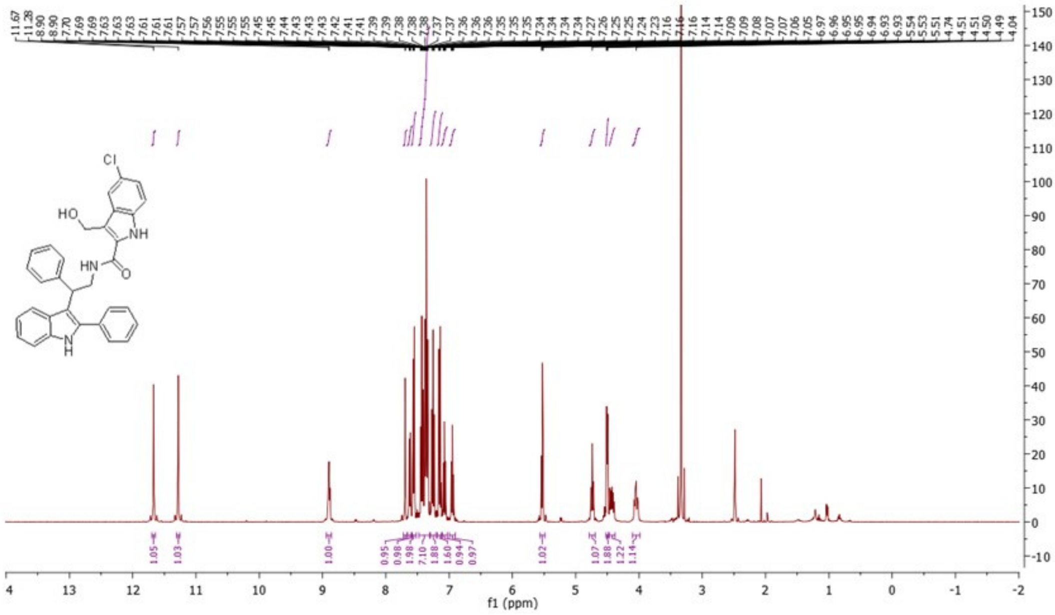

^{1}H NMR (400 MHz, DMSO-d_6) δ 11.67 (s, 1H), 11.28 (s, 1H), 8.90 (t, J = 6.3 Hz, 1H), 7.69 (d, J = 1.9 Hz, 1H), 7.62 d, J = 7.8 Hz, 1H), 7.59 – 7.52 (m, 2H), 7.48 – 7.31 (m, 7H), 7.25 (t, J = 8.4 Hz, 2H), 7.15 (dd, J = 8.7, 2.1 Hz, 2H), 7.07 (t, J = 8.1 Hz, 1H), 6.95 (t, J = 8.1 Hz, 1H), 5.53 (t, J = 5.4 Hz, 1H), 4.74 (t, J = 7.9 Hz, 1H), 4.50 (d, J = 5.5 Hz, 2H), 4.46 – 4.39 (m, 1H), 4.08 - 4.00 (m, 1H).

Expanded Spectrum of Vd

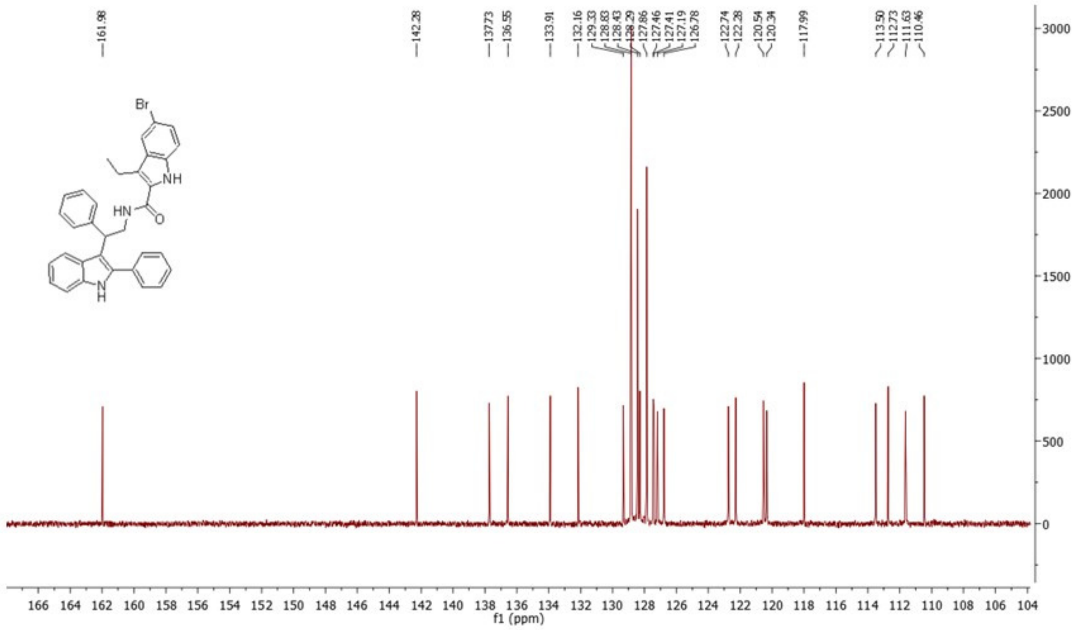

^{13}C NMR (101 MHz, cdcl$_3$) δ 161.98, 142.28, 137.73, 136.55, 133.91, 132.16, 129.33, 128.83, 128.43, 128.29, 127.86, 127.46, 127.41, 127.19, 126.78, 122.74, 122.28, 120.54, 120.34, 117.99, 113.50, 112.73, 111.63, 110.46, 43.47, 42.38, 17.78, 14.51.

Vd

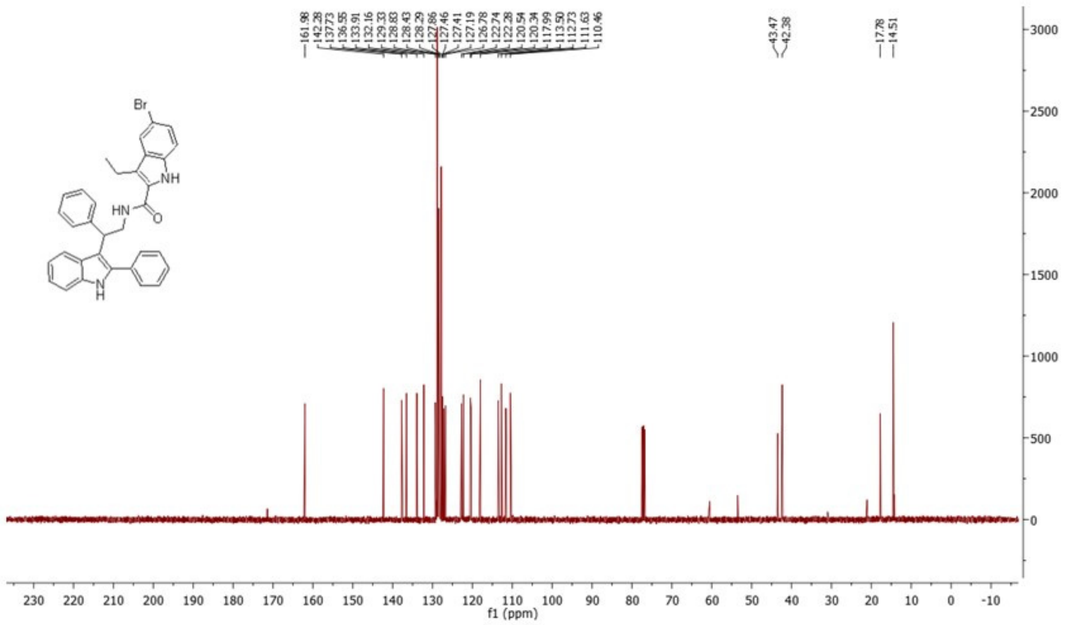

^{13}C NMR (101 MHz, cdcl3) δ 161.98, 142.28, 137.73, 136.55, 133.91, 132.16, 129.33, 128.83, 128.43, 128.29, 127.86, 127.46, 127.41, 127.19, 126.78, 122.74, 122.28, 120.54, 120.34, 117.99, 113.50, 112.73, 111.63, 110.46, 43.47, 42.38, 17.78, 14.51.

Expanded Spectrum of Vd

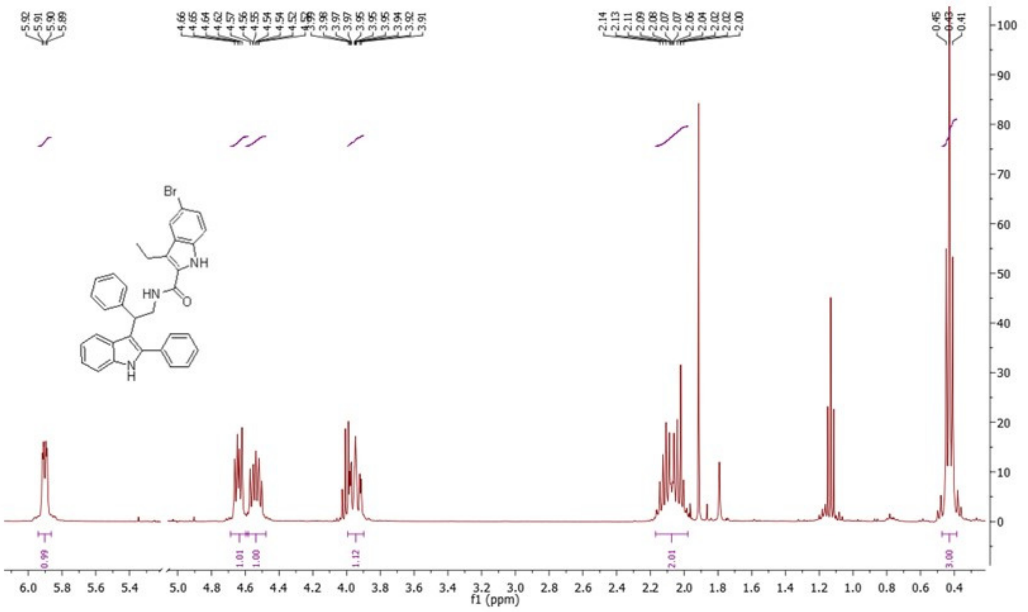

^{1}H NMR (400 MHz, Chloroform-*d*) δ 9.62 (s, 1H), 8.54 (s, 1H), 7.58 (d, J = 8.2 Hz, 1H), 7.45 (d, J = 1.8 Hz, 1H), 7.36 – 7.26 (m, 3H), 7.24 – 6.96 (m, 12H), 5.90 (t, J = 8.1 Hz, 1H), 4.64 (dd, J = 11.0, 6.4 Hz, 1H), 4.57 - 4.50 (m, 1H), 3.99 – 3.90 (m, 1H), 2.17 – 1.98 (m, 2H), 0.43 (t, J = 7.6 Hz, 3H).

Expanded Spectrum of Vd

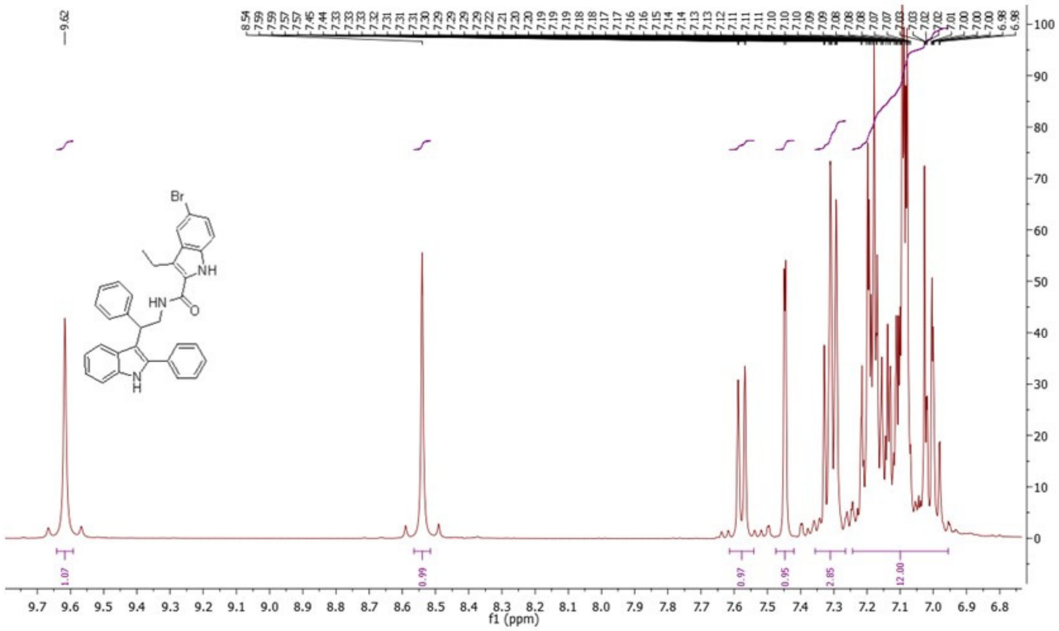

^{1}H NMR (400 MHz, Chloroform-*d*) δ 9.62 (s, 1H), 8.54 (s, 1H), 7.58 (d, *J* = 8.2 Hz, 1H), 7.45 (d, *J* = 1.8 Hz, 1H), 7.36 – 7.26 (m, 3H), 7.24 – 6.96 (m, 12H), 5.90 (t, *J* = 8.1 Hz, 1H), 4.64 (dd, *J* = 11.0, 6.4 Hz, 1H), 4.57 - 4.50 (m, 1H), 3.99 – 3.90 (m, 1H), 2.17 – 1.98 (m, 2H), 0.43 (t, *J* = 7.6 Hz, 3H).

Vd

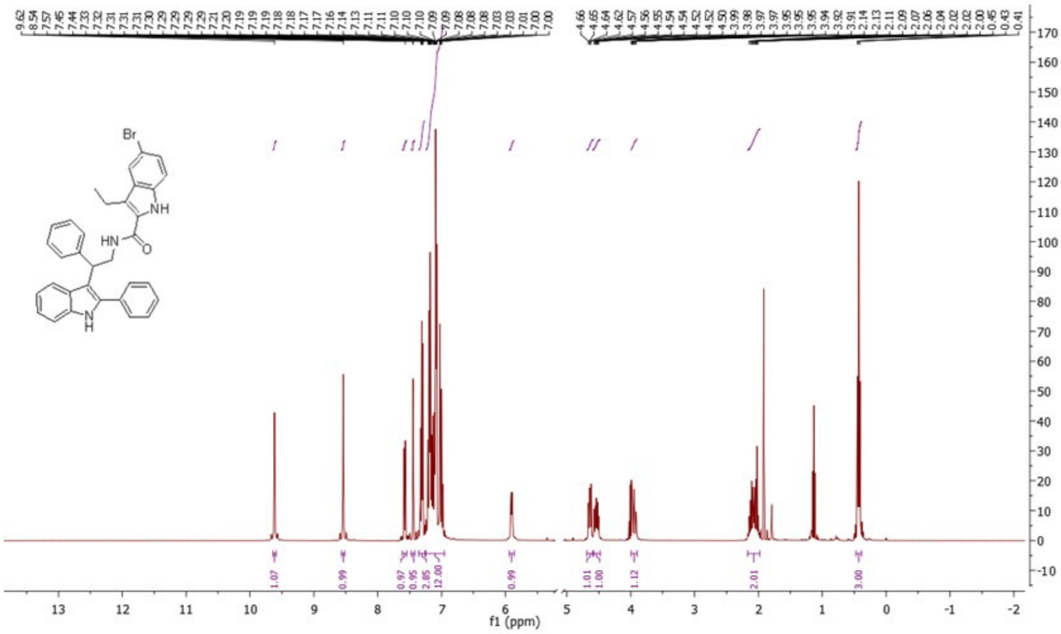

^{1}H NMR (400 MHz, Chloroform-*d*) δ 9.62 (s, 1H), 8.54 (s, 1H), 7.58 (d, *J* = 8.2 Hz, 1H), 7.45 (d, *J* = 1.8 Hz, 1H), 7.36 – 7.26 (m, 3H), 7.24 – 6.96 (m, 12H), 5.90 (t, *J* = 8.1 Hz, 1H), 4.64 (dd, *J* = 11.0, 6.4 Hz, 1H), 4.57 - 4.50 (m, 1H), 3.99 – 3.90 (m, 1H), 2.17 – 1.98 (m, 2H), 0.43 (t, *J* = 7.6 Hz, 3H).

Expanded Spectrum of Vc

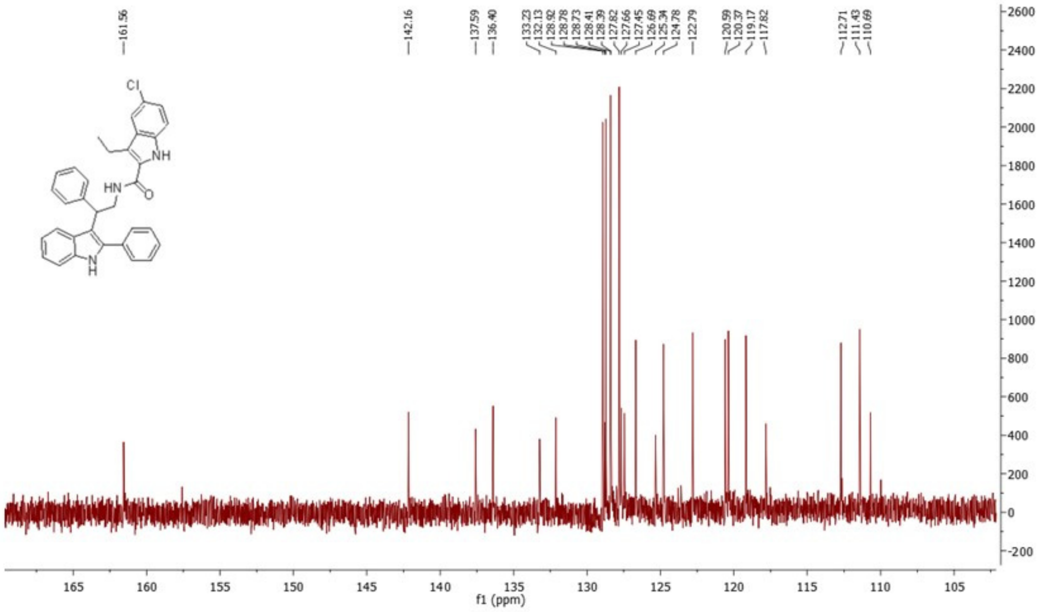

^{13}C NMR (101 MHz, cdcl$_3$) δ 161.56, 142.16, 137.59, 136.40, 133.23, 132.13, 128.92, 128.78, 128.73, 128.41, 128.39, 127.82, 127.66, 127.45, 126.69, 125.34, 124.78, 122.79, 120.59, 120.37, 119.17, 117.82, 112.71, 111.43, 110.69, 43.24, 42.28, 17.73, 14.42.

Vc

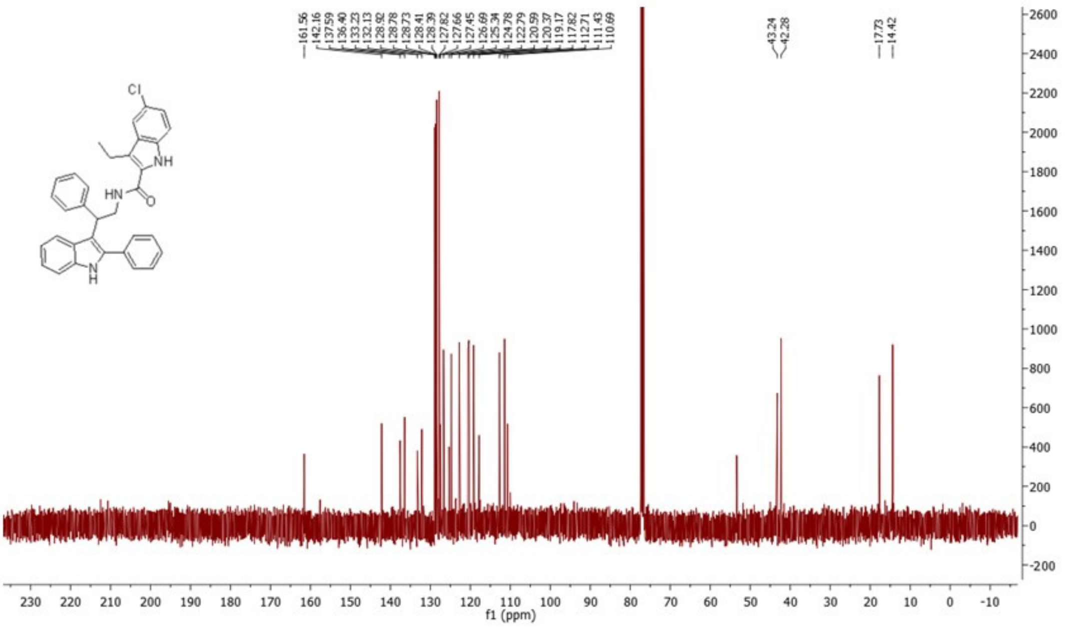

^{13}C NMR (101 MHz, cdcl₃) δ 161.56, 142.16, 137.59, 136.40, 133.23, 132.13, 128.92, 128.78, 128.73, 128.41, 128.39, 127.82, 127.66, 127.45, 126.69, 125.34, 124.78, 122.79, 120.59, 120.37, 119.17, 117.82, 112.71, 111.43, 110.69, 43.24, 42.28, 17.73, 14.42.

Expanded Spectrum of Vc

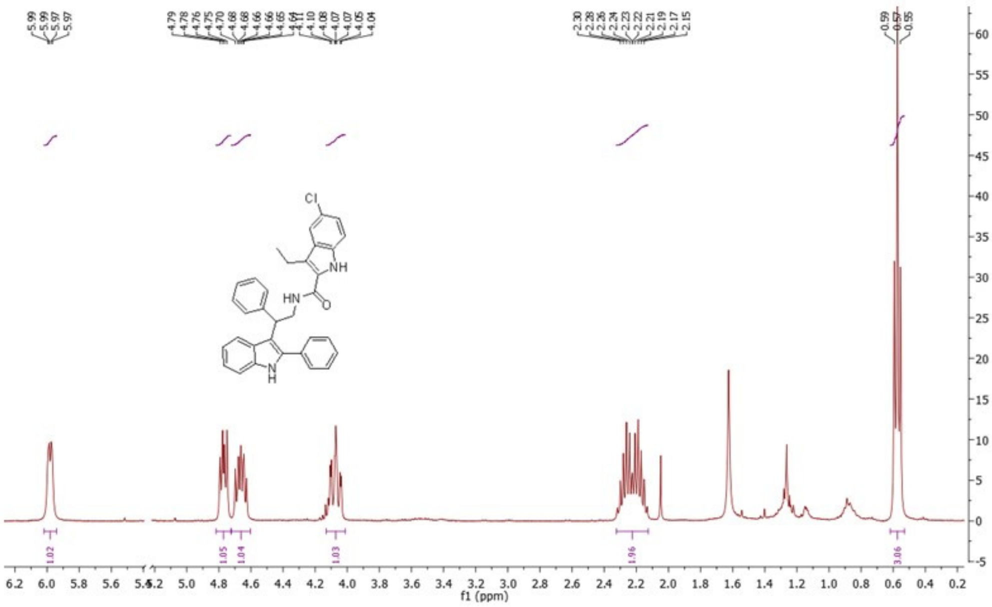

^{1}H NMR (400 MHz, Chloroform-*d*) δ 9.15 (s, 1H), 8.35 (s, 1H), 7.72 (d, *J* = 8.0 Hz, 1H), 7.51 – 7.40 (m, 4H), 7.39 – 7.19 (m, 10H), 7.18 – 7.11 (m, 2H), 5.98 (s, 1H), 4.79 - 4.75 (m, 1H), 4.72 – 4.60 (m, 1H), 4.12 - 4.03 (m, 1H), 2.30 - 2.15 (m, 2H), 0.57 (t, *J* = 7.6 Hz, 3H).

Expanded Spectrum of Vc

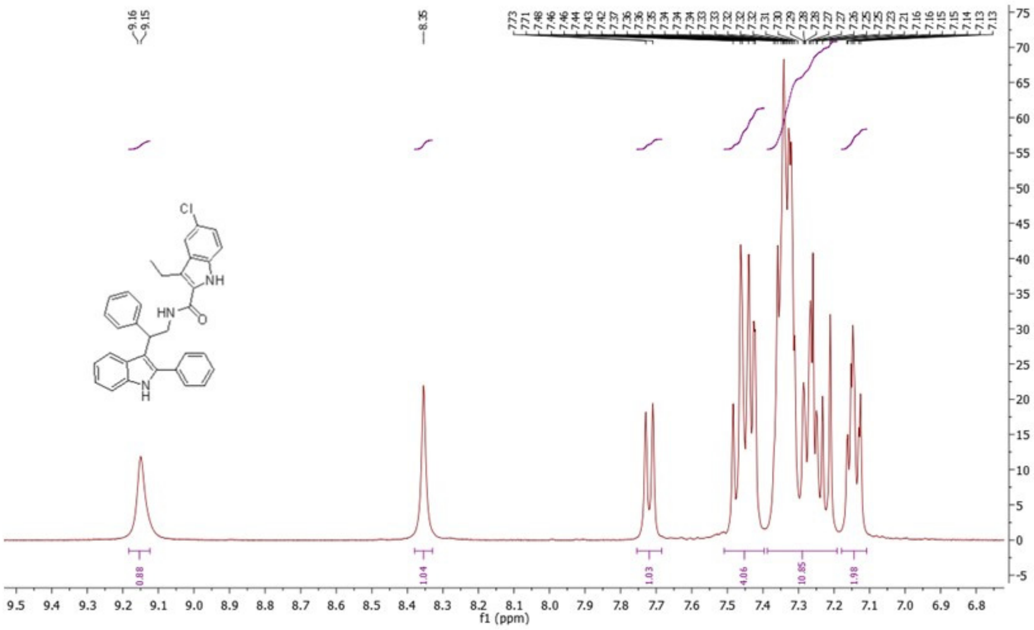

^{1}H NMR (400 MHz, Chloroform-*d*) δ 9.15 (s, 1H), 8.35 (s, 1H), 7.72 (d, J = 8.0 Hz, 1H), 7.51 – 7.40 (m, 4H), 7.39 – 7.19 (m, 10H), 7.18 – 7.11 (m, 2H), 5.98 (s, 1H), 4.79 - 4.75 (m, 1H), 4.72 – 4.60 (m, 1H), 4.12 - 4.03 (m, 1H), 2.30 - 2.15 (m, 2H), 0.57 (t, J = 7.6 Hz, 3H).

Vc

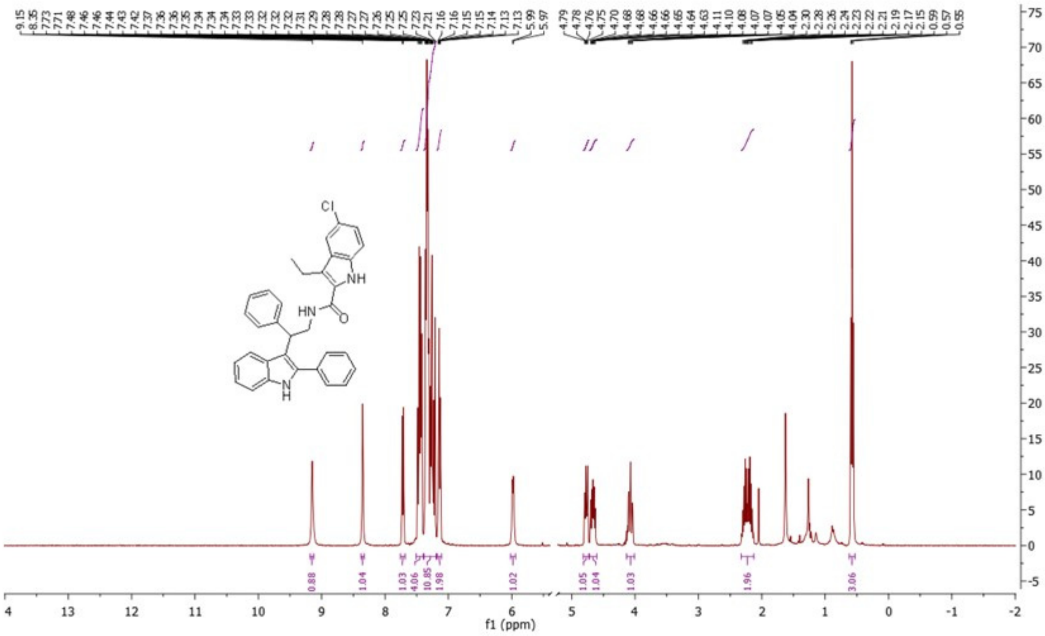

^{1}H NMR (400 MHz, Chloroform-*d*) δ 9.15 (s, 1H), 8.35 (s, 1H), 7.72 (d, *J* = 8.0 Hz, 1H), 7.51 – 7.40 (m, 4H), 7.39 – 7.19 (m, 10H), 7.18 – 7.11 (m, 2H), 5.98 (s, 1H), 4.79 - 4.75 (m, 1H), 4.72 – 4.60 (m, 1H), 4.12 - 4.03 (m, 1H), 2.30 - 2.15 (m, 2H), 0.57 (t, *J* = 7.6 Hz, 3H).

Expanded Spectrum of Vb

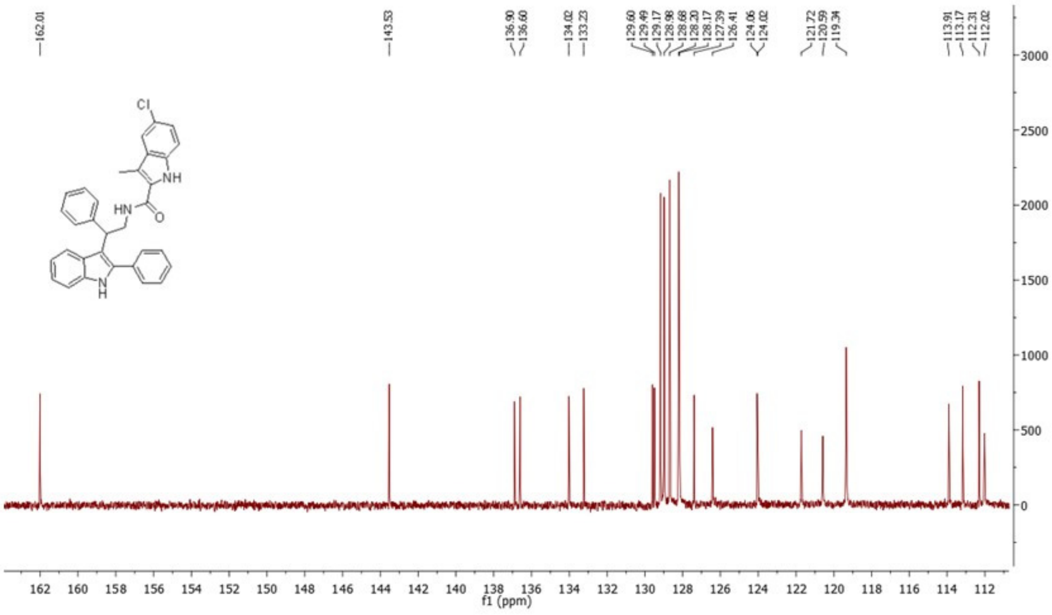

^{13}C NMR (101 MHz, dmso) δ 162.01, 143.53, 136.90, 136.60, 134.02, 133.23, 129.60, 129.49, 129.17, 128.98, 128.68, 128.20, 128.17, 127.39, 126.41, 124.06, 124.02, 121.72, 120.59, 119.34, 113.91, 113.17, 112.31, 112.02, 43.40, 41.74, 9.69.

Vb

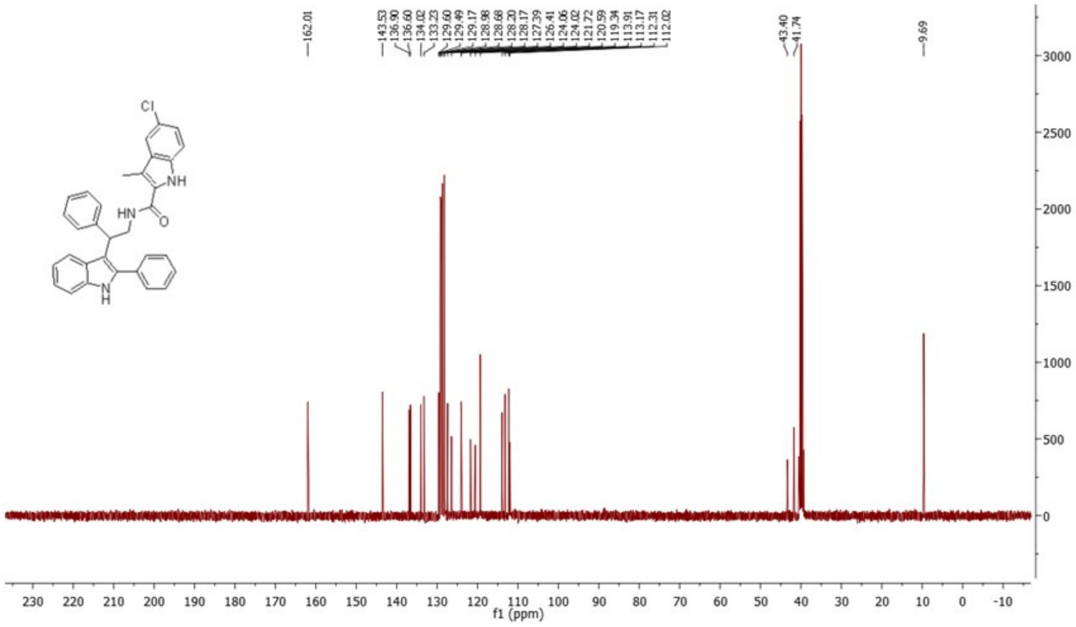

^{13}C NMR (101 MHz, dmso) δ 162.01, 143.53, 136.90, 136.60, 134.02, 133.23, 129.60, 129.49, 129.17, 128.98, 128.68, 128.20, 128.17, 127.39, 126.41, 124.06, 124.02, 121.72, 120.59, 119.34, 113.91, 113.17, 112.31, 112.02, 43.40, 41.74, 9.69.

Expanded Spectrum of Vb

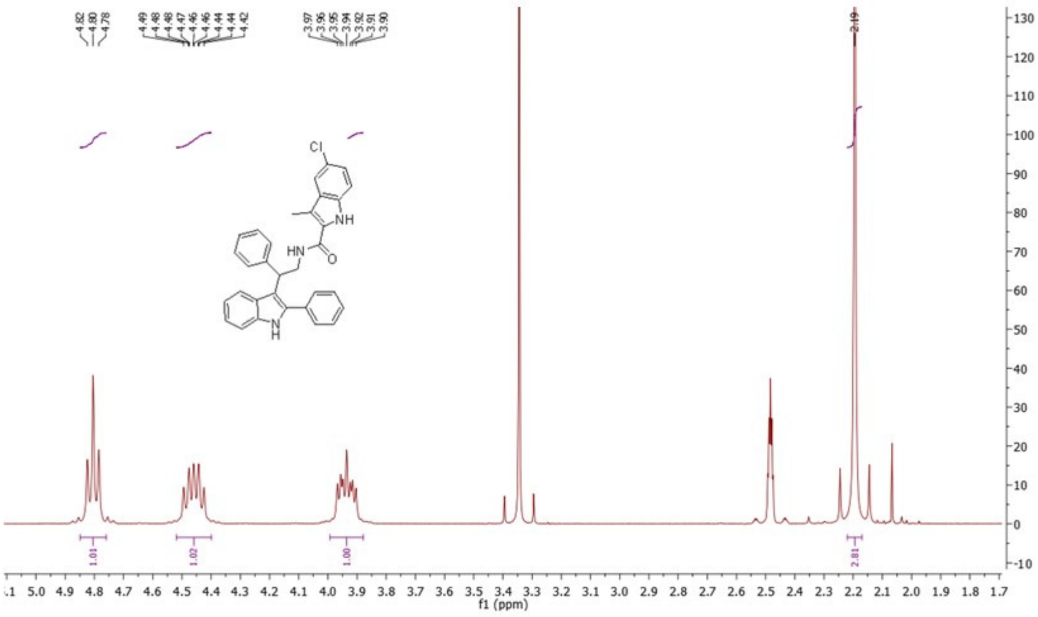

^{1}H NMR (400 MHz, DMSO-d_6) δ 11.32 - 11.30 (m, 2H), 7.80 (t, *J* = 5.8 Hz, 1H), 7.65 – 7.51 (m, 4H), 7.45 – 7.30 (m, 7H), 7.26 (t, *J* = 7.6 Hz, 2H), 7.19 – 7.05 (m, 3H), 6.97 (t, *J* = 8.1 Hz, 1H), 4.80 (t, *J* = 7.9 Hz, 1H), 4.46 (dd, *J* = 13.1, 8.1 Hz, 1H), 3.94 (dd, *J* = 12.7, 7.8 Hz, 1H), 2.19 (s, 3H).

Expanded Spectrum of Vb

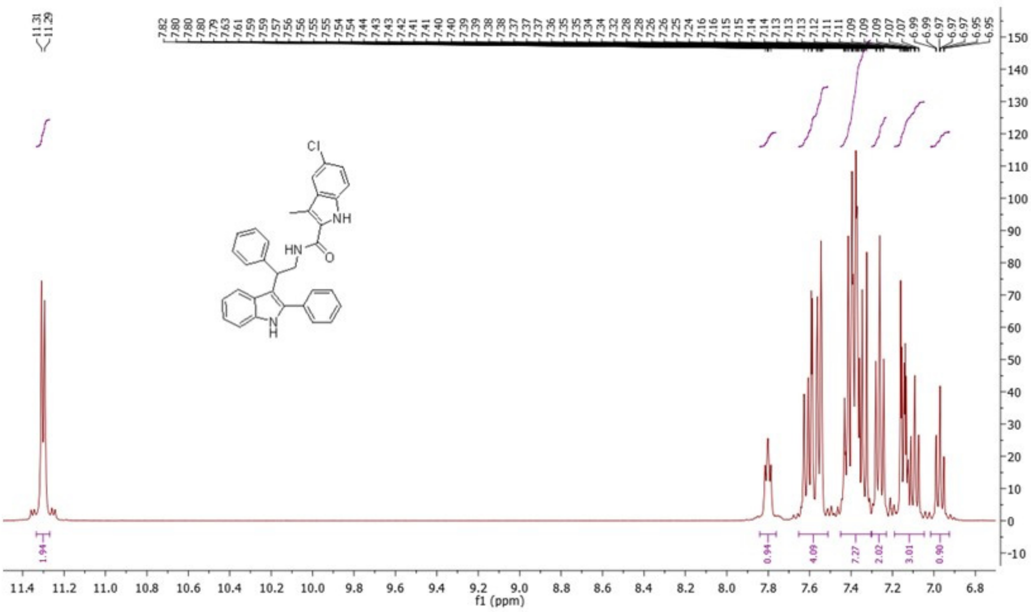

^{1}H NMR (400 MHz, DMSO-d_6) δ 11.32 - 11.30 (m, 2H), 7.80 (t, J = 5.8 Hz, 1H), 7.65 – 7.51 (m, 4H), 7.45 – 7.30 (m, 7H), 7.26 (t, J = 7.6 Hz, 2H), 7.19 – 7.05 (m, 3H), 6.97 (t, J = 8.1 Hz, 1H), 4.80 (t, J = 7.9 Hz, 1H), 4.46 (dd, J = 13.1, 8.1 Hz, 1H), 3.94 (dd, J = 12.7, 7.8 Hz, 1H), 2.19 (s, 3H).

Vb

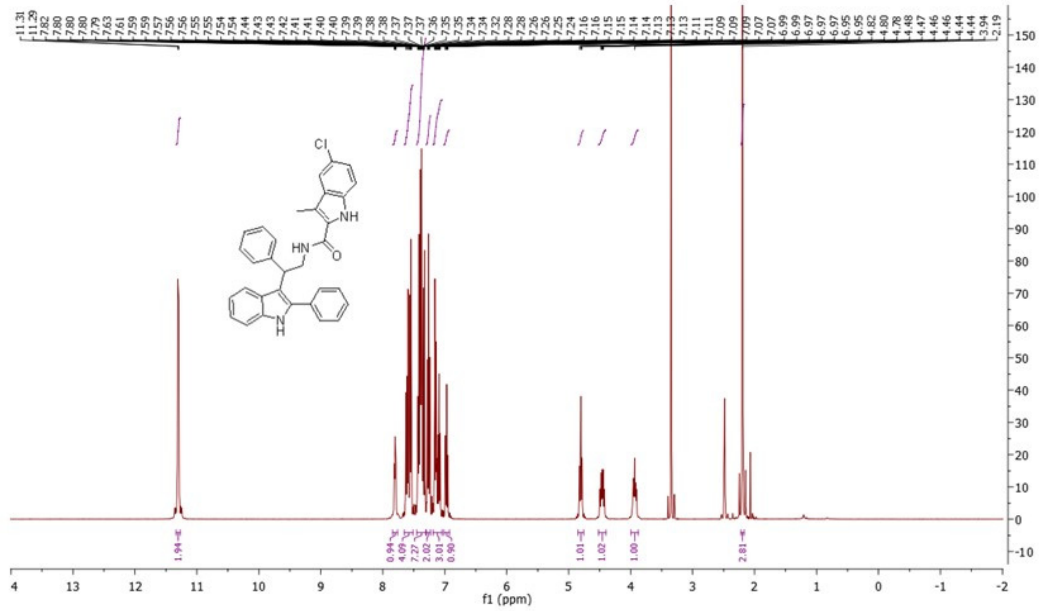

^{1}H NMR (400 MHz, DMSO-d_6) δ 11.32 - 11.30 (m, 2H), 7.80 (t, J = 5.8 Hz, 1H), 7.65 – 7.51 (m, 4H), 7.45 – 7.30 (m, 7H), 7.26 (t, J = 7.6 Hz, 2H), 7.19 – 7.05 (m, 3H), 6.97 (t, J = 8.1 Hz, 1H), 4.80 (t, J = 7.9 Hz, 1H), 4.46 (dd, J = 13.1, 8.1 Hz, 1H), 3.94 (dd, J = 12.7, 7.8 Hz, 1H), 2.19 (s, 3H).

Expanded Spectrum of Va

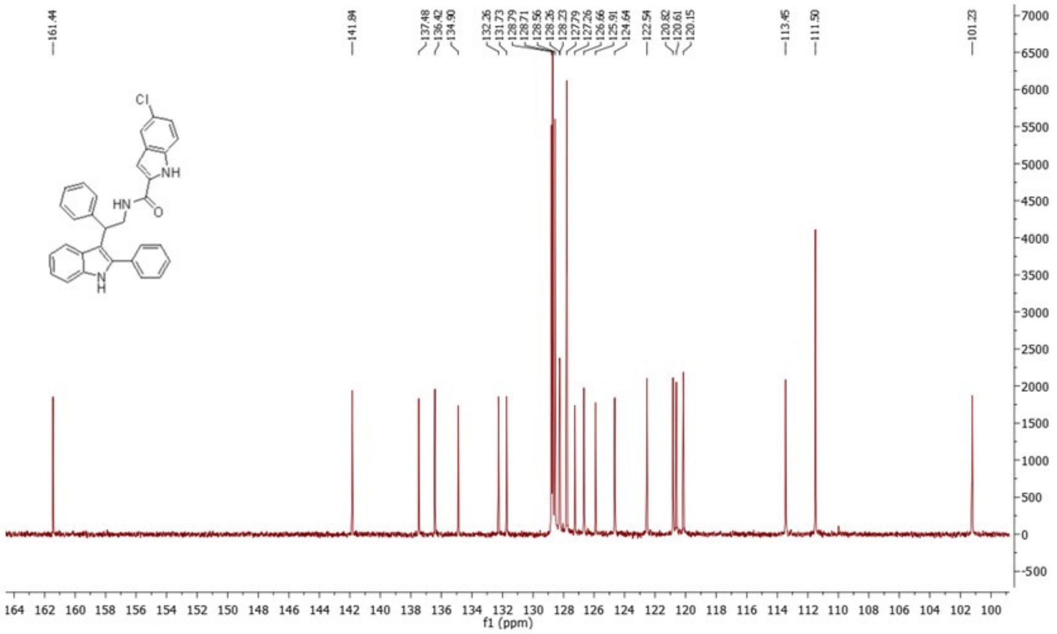

^{13}C NMR (101 MHz, cdcl$_3$) δ 161.44, 141.84, 137.48, 136.42, 134.90, 132.26, 131.73, 128.79, 128.71, 128.56, 128.26, 128.23, 127.79, 127.26, 126.66, 125.91, 124.64, 122.54, 120.82, 120.61, 120.15, 113.45, 111.50, 101.23, 43.35, 41.54.

Va

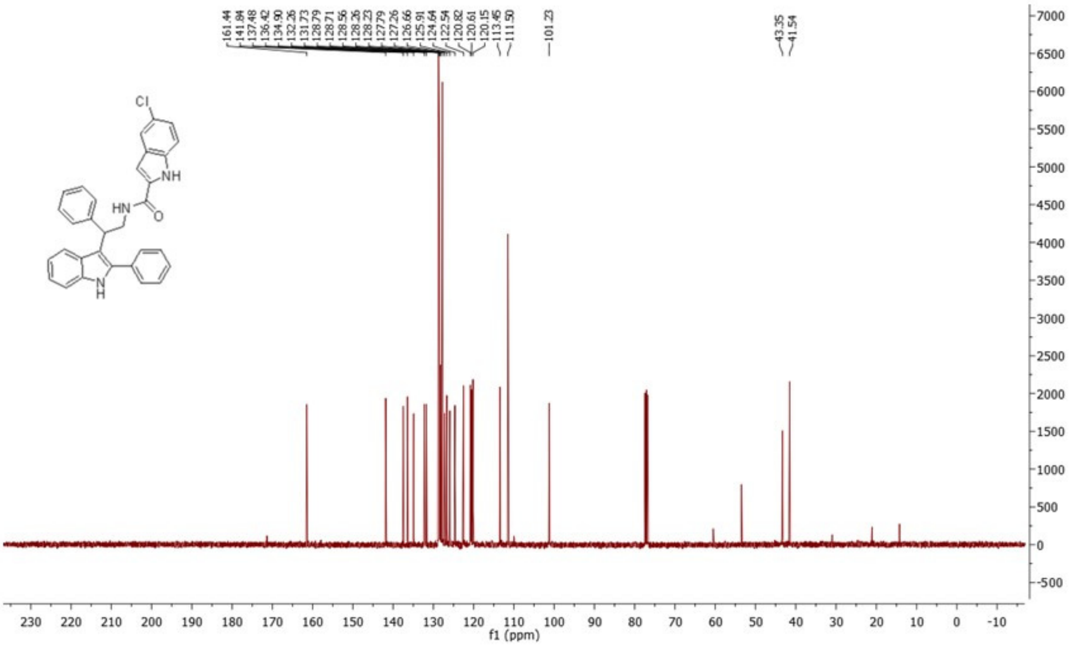

^{13}C NMR (101 MHz, cdcl$_3$) δ 161.44, 141.84, 137.48, 136.42, 134.90, 132.26, 131.73, 128.79, 128.71, 128.56, 128.26, 128.23, 127.79, 127.26, 126.66, 125.91, 124.64, 122.54, 120.82, 120.61, 120.15, 113.45, 111.50, 101.23, 43.35, 41.54.

Expanded Spectrum of Va

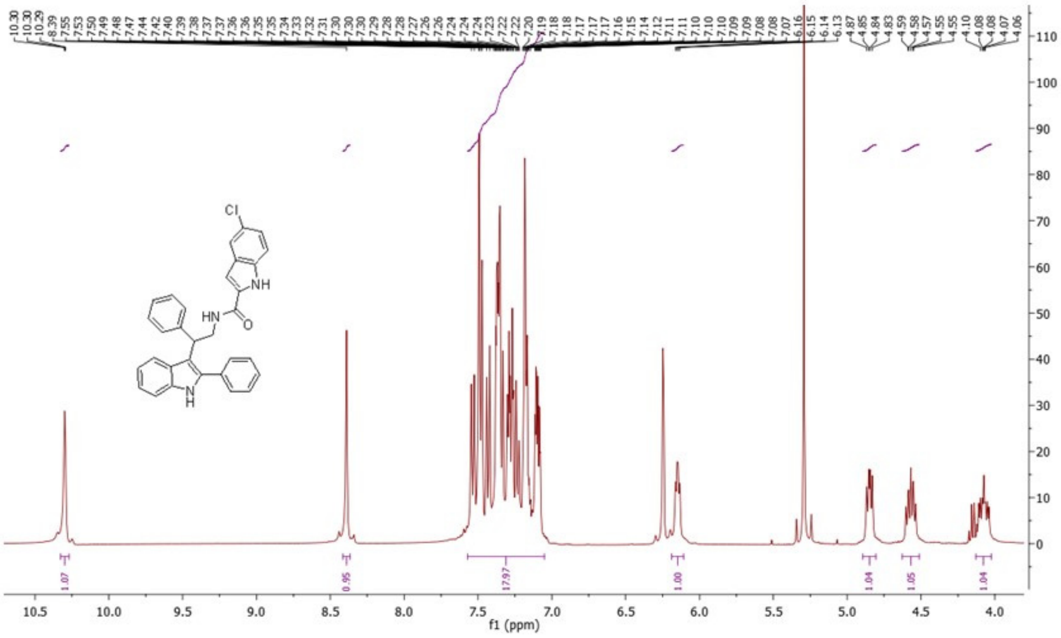

^{1}H NMR (400 MHz, Chloroform-*d*) δ 10.30 (s, 1H), 8.39 (s, 1H), 7.57 – 7.05 (m, 18H), 6.15 (t, *J* = 7.2 Hz, 1H), 4.85 (dd, *J* = 9.8, 5.8 Hz, 1H), 4.63 – 4.51 (m, 1H), 4.18 – 4.01 (m, 1H).

Va

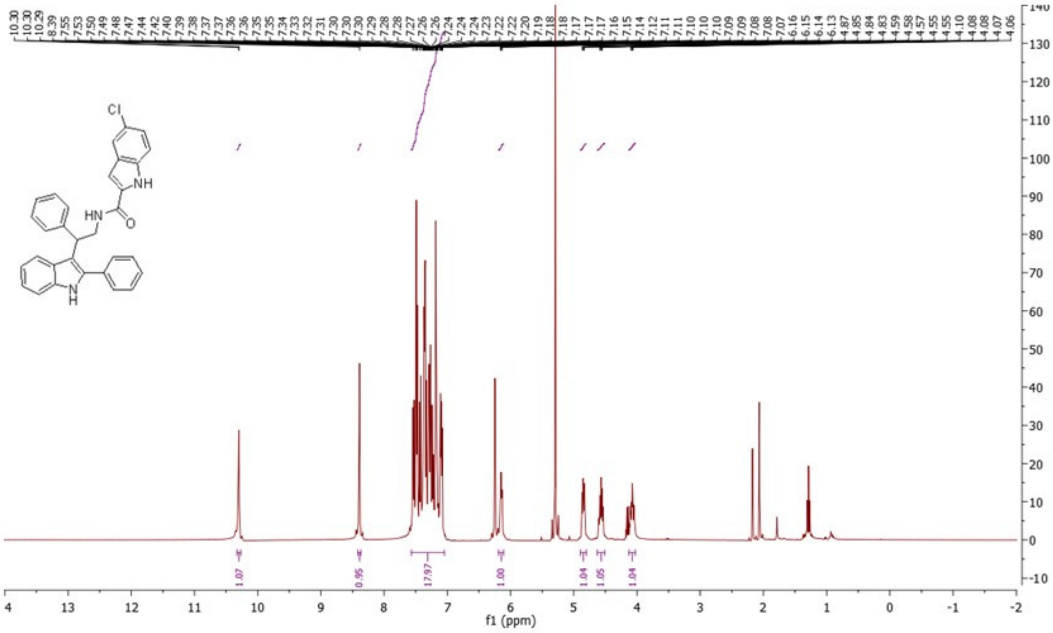

^{1}H NMR (400 MHz, Chloroform-*d*) δ 10.30 (s, 1H), 8.39 (s, 1H), 7.57 – 7.05 (m, 18H), 6.15 (t, *J* = 7.2 Hz, 1H), 4.85 (dd, *J* = 9.8, 5.8 Hz, 1H), 4.63 – 4.51 (m, 1H), 4.18 – 4.01 (m, 1H).

Expanded Spectrum of IV

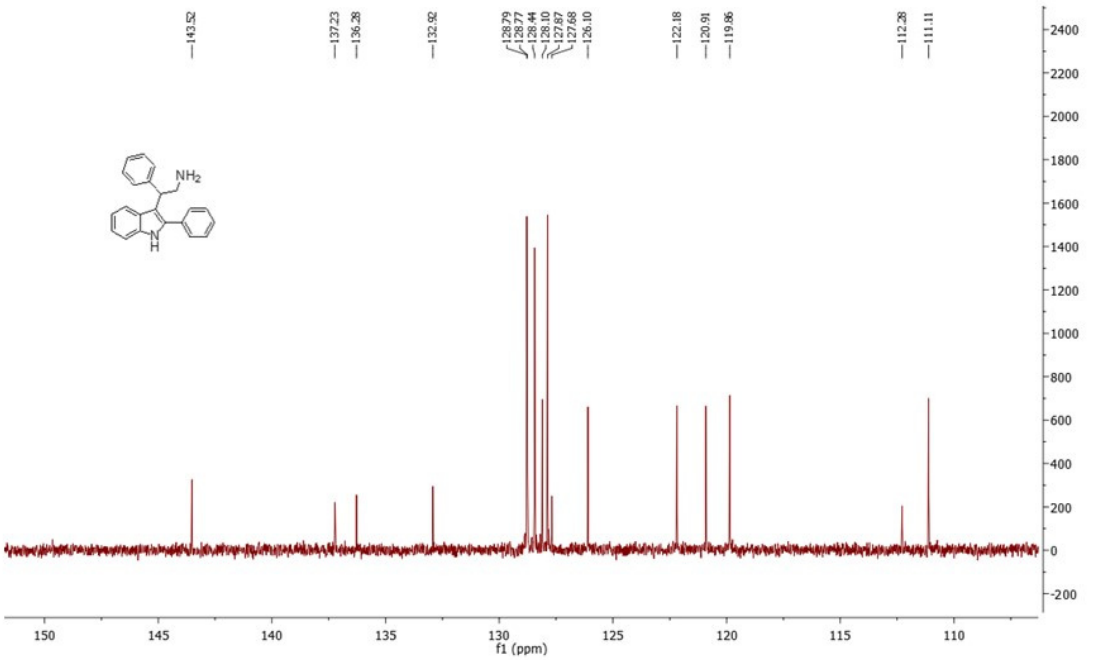

^{13}C NMR (101 MHz, cdcl$_3$) δ 143.52, 137.23, 136.28, 132.92, 128.79, 128.77, 128.44, 128.10, 127.87, 127.68, 126.10, 122.18, 120.91, 119.86, 112.28, 111.11, 46.58, 46.55.

IV

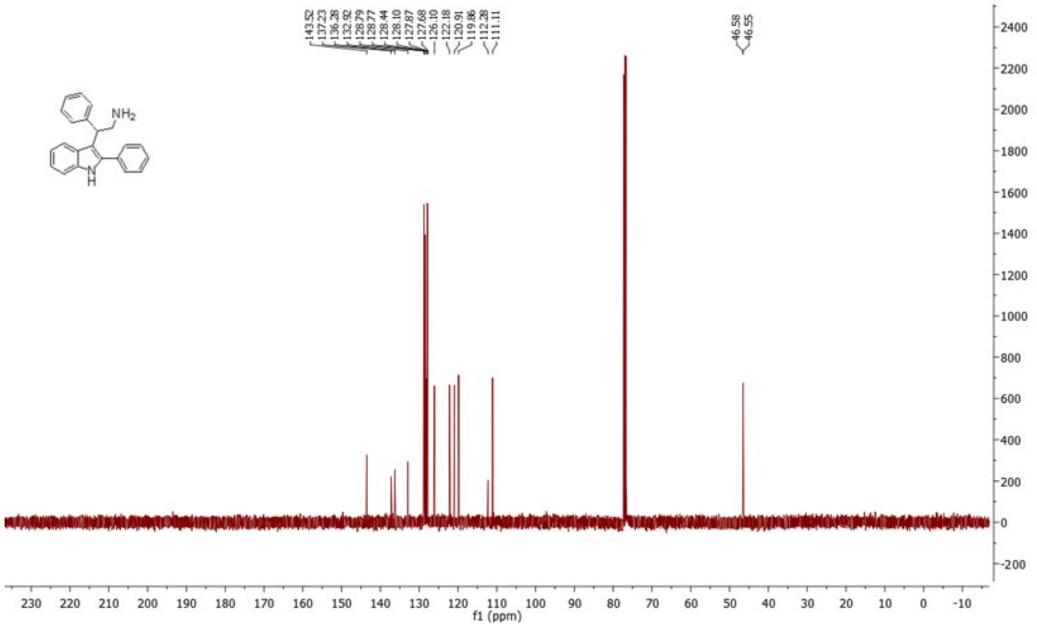

^{13}C NMR (101 MHz, cdcl$_3$) δ 143.52, 137.23, 136.28, 132.92, 128.79, 128.77, 128.44, 128.10, 127.87, 127.68, 126.10, 122.18, 120.91, 119.86, 112.28, 111.11, 46.58, 46.55.

Expanded Spectrum of IV

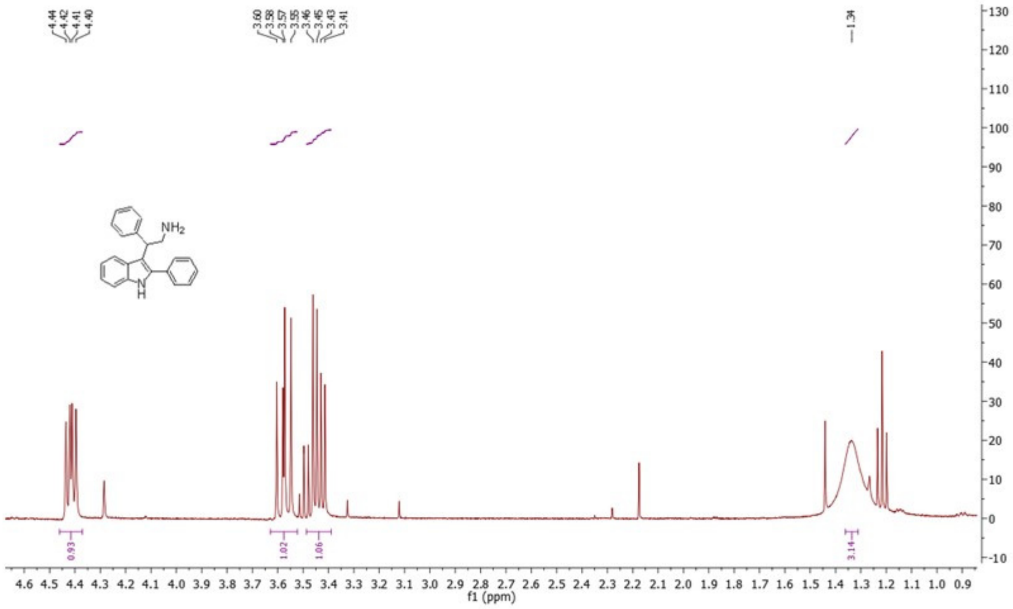

^{1}H NMR (400 MHz, Chloroform-*d*) δ 8.28 (s, 1H), 7.65 (d, *J* = 8.1 Hz, 1H), 7.53 – 7.33 (m, 8H), 7.35 – 7.16 (m, 4H), 7.09 (t, *J* = 8.1 Hz, 1H), 4.42 (dd, *J* = 9.7, 6.2 Hz, 1H), 3.58 (dd, *J* = 12.6, 9.8 Hz, 1H), 3.44 (dd, *J* = 12.7, 6.2 Hz, 1H), 1.34 (s, 2H).

Expanded Spectrum of IV

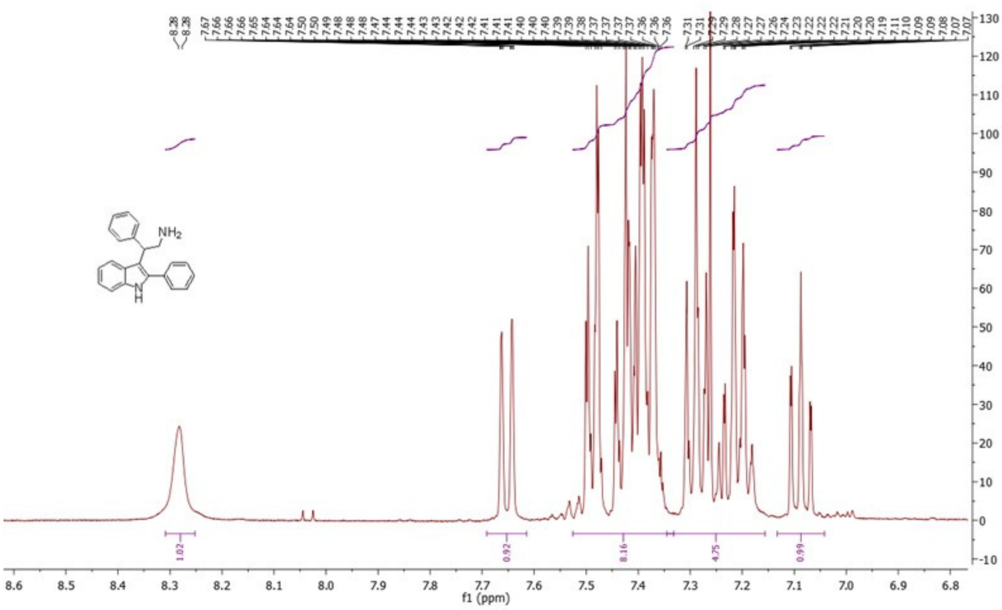

^{1}H NMR (400 MHz, Chloroform-*d*) δ 8.28 (s, 1H), 7.65 (d, *J* = 8.1 Hz, 1H), 7.53 – 7.33 (m, 8H), 7.35 – 7.16 (m, 4H), 7.09 (t, *J* = 8.1 Hz, 1H), 4.42 (dd, *J* = 9.7, 6.2 Hz, 1H), 3.58 (dd, *J* = 12.6, 9.8 Hz, 1H), 3.44 (dd, *J* = 12.7, 6.2 Hz, 1H), 1.34 (s, 2H).

IV

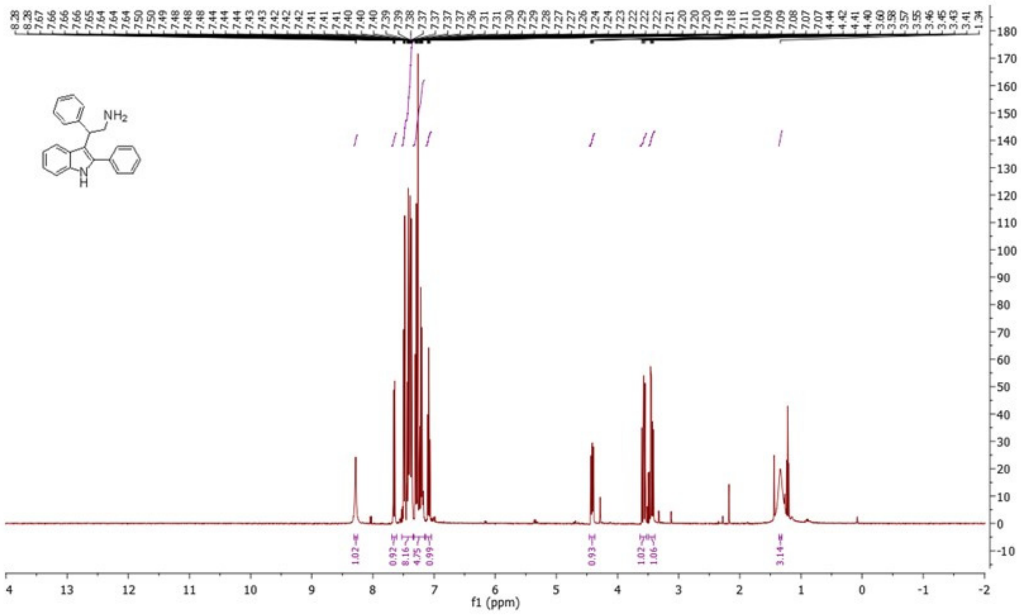

^{1}H NMR (400 MHz, Chloroform-*d*) δ 8.28 (s, 1H), 7.65 (d, *J* = 8.1 Hz, 1H), 7.53 – 7.33 (m, 8H), 7.35 – 7.16 (m, 4H), 7.09 (t, *J* = 8.1 Hz, 1H), 4.42 (dd, *J* = 9.7, 6.2 Hz, 1H), 3.58 (dd, *J* = 12.6, 9.8 Hz, 1H), 3.44 (dd, *J* = 12.7, 6.2 Hz, 1H), 1.34 (s, 2H).

4. EXPERIMENTAL

4.1. Chemistry

Materials and methods

Purification was carried out by flash column chromatography. The silica gel used was Merck 60 (230-400 mesh). Thin layer chromatographic analysis was carried out using Merck aluminium-backed plates coated with silica gel 60 F_{254}. Melting points were determined using a Gallen Kamp melting point apparatus and are uncorrected. Infrared spectra were recorded with a Nicolet Avatar 320 FTIR spectrometer. Components were visualised using potassium permanganate solution and UV light NMR Spectra were recorded on a Varian Inova 400 MHz and a Bruker AC250 250 MHz for proton and carbon. All numbers referring to NMR data obtained are in parts per million (ppm). Mass analyses were carried out by the EPSRC mass spectrometry centre in Swansea and Thermo Instruments MS system (LTQ XL/LTQ Orbitrap Discovery) coupled to a Thermo Instruments HPLC system (Accela PDA detector, Accela PDA autosampler and Pump) in University of Aberdeen.

4.2. Biological evaluation

4.2.1. Cytotoxic activity using MTT Assay and evaluation of IC_{50}

4.2.1.1. MTT assay

MTT assay was performed to investigate the effect of the synthesized compounds on mammary epithelial cells (MCF-10A). The cells were propagated in medium consisting of Ham's F-12 medium/ Dulbecco's modified Eagle's medium (DMEM) (1:1) supplemented with 10% foetal calf serum, 2 mM glutamine, insulin (10 μg/mL), hydrocortisone (500 ng/mL) and epidermal growth factor (20 ng/mL). Trypsin ethylenediamine tetra acetic acid (EDTA) was used to passage the cells after every 2-3 days. 96-well flat-bottomed cell culture plates were used to seed the cells at a density of 104 cells mL^{-1}. The medium was aspirated from all the wells of culture plates after 24 h followed by the addition of synthesized compounds (in 200 μL medium to yield a final concentration of 0.1% (v/v) dimethyl sulfoxide) into individual wells of the plates. Four wells were designated to a single compound. The plates were allowed to incubate at 37°C for 96 h. Afterwards, the medium was aspirated and 3-[4,5-dimethylthiazol-2-yl]-2,5-diphenyltetrazolium bromide (MTT) (0.4 mg/mL) in medium was added to each well and subsequently incubated for 3 h. The medium was aspirated and 150 μL dimethyl sulfoxide (DMSO) was added to each well. The plates were vortexed followed by the measurement of absorbance at 540 nm on a microplate reader. The results were presented as inhibition (%) of proliferation in contrast to controls comprising 0.1% DMSO.

4.2.1.2. Assay for antiproliferative effect

To explore the antiproliferative potential of compounds propidium iodide fluorescence assay was performed using different cell lines such as Panc-1 (pancreas cancer cell line), MCF-7 (breast cancer cell line), HT-29 (colon cancer cell line) and A-549 (epithelial cancer cell line), respectively. To calculate the total nuclear DNA, a fluorescent dye (propidium iodide, PI) is used which can attach to the DNA, thus offering a quick and precise technique. PI cannot pass through the cell membrane and its signal intensity can be considered as directly proportional to quantity of cellular DNA. Cells whose cell membranes are damaged or have changed permeability are counted as dead ones. The assay was performed by seeding the cells of different cell lines at a density of 3000-7500 cells/well (in200µl medium) in culture plates followed by incubation for 24h at 37 °C in humidified 5%CO2/95% air atmospheric conditions. The medium was removed; the compounds were added to the plates at 10 µM concentrations (in 0.1% DMSO) in triplicates, followed by incubation for 48h. DMSO (0.1%) was used as control. After incubation, medium was removed followed by the addition of PI (25 µl, 50µg/mL in water/medium) to each well of the plates. At -80 °C, the plates were allowed to freeze for 24 h, followed by thawing at 25oC. A fluorometer (Polar-Star BMG Tech) was used to record the readings at excitation and emission wavelengths of 530 and 620 nm for each well. The percentage cytotoxicity of compounds was calculated using the following formula:

$$\% \text{ Cytotoxicity} = \frac{A_C - A_{TC}}{A_C} \times 100$$

Where A*TC*= Absorbance of treated cells and AC= Absorbance of control. Erlotinib was used as positive control in the assay.

4.2.2. EGFR inhibitory assay

Baculoviral expression vectors including pBlueBacHis2B and pFASTBacHTc were used separately to clone 1.6 kb cDNA coding for EGFR cytoplasmic domain (EGFR-CD, amino acids 645–1186). 5′ upstream to the EGFR sequence comprised a sequence that encoded (His)6. Sf-9 cells were infected for 72h for protein expression. The pellets of Sf-9 cells were solubilized in a buffer containing sodium vanadate (100 μM), aprotinin (10 μg/mL), triton (1%), HEPES buffer(50mM), ammonium molybdate (10 μM), benzamidine HCl (16 μg/mL), NaCl (10 mM),leupeptin (10 μg/mL) and pepstatin (10 μg/mL) at 0°C for 20 min at pH 7.4, followed by centrifugation for 20 min. To eliminate the nonspecifically bound material, a Ni-NTA super flow packed column was used to pass through and wash the crude extract supernatant first with 10mM and then with 100 mM imidazole. Histidine-linked proteins were first eluted with 250 and then with 500 mM imidazole subsequent to dialysis against NaCl (50 mM), HEPES (20 mM), glycerol (10%) and 1 μg/mL each of aprotinin, leupeptin and pepstatin for 120 min. The purification was performed either at 4 °C or on ice. To record autophosphorylation level, EGFR kinase assay was carried out on the basis of DELFIA/Time-Resolved Fluorometry. The compounds were first dissolved in DMSO absolute, subsequent to dilution to appropriate concentration using HEPES (25 mM) at pH 7.4. Each compound (10 μL) was incubated with recombinant enzyme (10 μL, 5 ng for EGFR, 1:80 dilution in 100 mM HEPES) for 10 min at 25°C, subsequent to the addition of 5X buffer (10 μL, containing 2 mM MnCl2, 100 μM Na3VO4, 20 mM HEPES and 1 mM DTT) and ATP-MgCl2 (20 μL, containing 0.1 mM ATP and 50 mM MgCl2) and incubation for 1h. The negative and positive controls were included in each plate by the incubation of enzyme either with or without ATP-MgCl2. The liquid was removed after incubation and the plates were washed thrice using wash buffer. Europium-tagged antiphosphotyrosine antibody (75 μL, 400 ng) was

added to each well followed by incubation of 1h and then washing of the plates using buffer. The enhancement solution was added to each well and the signal was recorded at excitation and emission wavelengths of 340 at 615 nm. The autophosphorylation percentage inhibition by compounds was calculated using the following equation:

$$100\% - [(negative\ control)/(positive\ control) - (negative\ control)]$$

Using the curves of percentage inhibition of eight concentrations of each compound, IC50 was calculated. Majority of signals detected by antiphosphotyrosine antibody were from EGFR because the enzyme preparation contained low impurities.

4.2.3. BRAF^{V600E} inhibitory assay

V^{600E} mutant BRAF kinase assay was performed to investigate the activity of tested compounds against BRAF. Mouse full-length GST-tagged BRAF^{V600E} (7.5 ng, Invitrogen, PV3849) was pre-incubated with drug (1 µL) and assay dilution buffer (4 µL) for 60 min at 25ºC. In assay dilution buffer, a solution (5 µL) containing $MgCl_2$ (30 mM), ATP (200 µM), recombinant human full length (200 ng) and *N*-terminal His-tagged MEK1 (Invitrogen) was added to start the assay, subsequent to incubation for 25 min at 25ºC. The assay was stopped using 5X protein denaturing buffer (LDS) solution (5 µL). To further denature the protein, heat (70° C) was applied for 5 min. 4-12% precast NuPage gel plates (Invitrogen) were used to carry out electrophoresis (at 200 V). 10 µL of each reaction was loaded into the precast plates and electrophoresis was allowed to proceed. After completion of electrophoresis, the front part of the precast gel plate (holding hot ATP) was cut and afterwards cast-off. The dried gel was developed using a phosphor screen. A reaction without active enzyme was used as negative control while that containing no inhibitor served as positive control. To study the effect of compounds on cell-based pERK1/2 activity in

cancer cells, commercially available ELISA kits (Invitrogen) were used according to manufacturer's instructions.

4.2.4. VEGFR-2 inhibitory assay

Prepare all the solutions using autoclaved, deionized water and analytical grade reagents. Prepare and store all the reagents at room temperature (unless indicated otherwise). EDTA solution (0.5 M, pH 8.0): add 95 mL ultra-pure water to 14.612 g EDTA, adjust the pH to 8.0 using NaOH solution, add 5 mL ultra-pure water; 1×Kinase Assay Buffer: add 25 mL HEPES solution (1 M), 190.175 mg EGTA, 5 mL MgCl2 solution (1 M), 1 mL DTT, 50 μL tween-20, 450 mL ultrapure water, adjust pH to 7.5 and constant volume to 500 mL using ultrapure water; 4×Stop solution (40 mM): Mix 0.8 mL of the foregoing EDTA solution, 1 mL 10×Detection Buffer and 8.2 mL ultra-pure water; 1×Detection Buffer: Mix 1 mL 10×Detection Buffer with 9 mL water; 4×VEGFR Kinase solution (1.74 nM, 521 times diluted, stored on ice): 1.2 μL VEGFR mother liquor (0.909 μM) was added to 624 μL 1×Kinase Assay Buffer and mixed; 4×ULight™-labeled JAK1 (substrate) (200 nM, 25 times diluted): 24 μL ULight™-labeled JAK1 (mother liquor concentration 5 μM) was added to 576 μL 1×Kinase Assay Buffer and mixed; 4×ATP Solution (40 μM, 250 times diluted): add 3 μL ATP solution (10 mM) to 747 μL 1×Kinase Assay Buffer and mixed; 4×Detection Mix (8 nM, 390.6 times diluted): 3 μL Europium- antiphospho-tyrosine antibody (PT66) (3.125 μM) was added to 1169 μL1×Detection Buffer and mixed; 2×substrate/ATP Mix: 560 μL foregoing 4×ULight™-labeled JAK1 and 560 μL 4×ATP solution and mixed (prepared before use). The assays used an ULight-labeled peptide substrate and a Europium-W1024-labeled antiphosphotyrosine antibody. The VEGFR-2 was purchased from Carna Biosciences, Inc. (New York, USA). The 384-well plates were obtained from PerkinElmer. Compounds were dissolved in DMSO and diluted to 11 concentrations at a tripling rate from 2.500

μM to 0.042 nM and added 2.5 μL to 384-well plates. 5 μL 2×VEGFR-2 kinase solution (0.5 nM) was added to 384-well plates homogeneous mixing and pre-reaction at room temperature

for 30 min. Next, 2.5 μL 4×Ultra ULightTM-JAK-1(Tyr1023) Peptide (200 nM)/ATP (40 μM) was added to the corresponding wells of a 384-well plate. Negative control: 2.5 μL/well 4×substrate/ATP mixture and 7.5 μL 1×kinase assay buffer in 384-well plate well. Positive control: 2.5 μL/well 4×substrate/ATP mixture, 2.5 μL/well 1×kinase assay buffer with 16% DMSO, 5 μL/well 2×VEGFR-2 kinase solution was added to the 384-well plate, The final concentration of DMSO in the mixing system was 4%. After incubation at room temperature and dark for 60 min, 5 μL 4×stop solution was added to corresponding wells to react for 5 min and then 5 μL 4×detection mix was added to the corresponding wells of a 384-well plate. The mixture was centrifugally mixed and stayed for 60 min at room temperature for color development. The plate was read using a Envision plate reader. The inhibition rate (%) = (positive well reading-compound well reading)/(positive well reading-negative well reading)×100. The corresponding IC_{50} values were calculated using GraphPad Prism 5.0.

4.2.5. Caspase-3 activation assay

Allow all reagents to reach room temperature before use. Gently mix all liquid reagents prior to use. Determine the number of 8-well strips needed for the assay. Insert these in the frame(s) for current use. Add 100 μl of the *Standard Diluent Buffer* to the zero standard wells. Well(s) reserved for chromogen blank should be left empty. Add 100 μl of standards and controls or diluted samples to the appropriate microtiter wells. The sample dilution chosen should be optimized for each experimental system. Tap gently on side of plate to mix. Cover wells with *plate cover* and incubate for 2 hours at room temperature. Thoroughly aspirate or decant solution from wells and discard the liquid, Wash wells 4 times. Pipette 100 μl of *Caspase-3 (Active) Detection Antibod*y solution

into each well except the chromogen blank(s). Tap gently on the side of the plate to mix. Cover plate with *plate cover* and incubate for 1 hour at room temperature. Thoroughly aspirate or decant solution from wells and discard the liquid, Wash wells 4 times. Add 100 µl Anti-Rabbit IgG HRP Working Solution to each well except the chromogen blank(s). Prepare the working dilution as described in Preparing IgG HRP. Cover wells with the *plate cover* and incubate for 30 minutes at room temperature. Thoroughly aspirate or decant solution from wells and discard the liquid. Wash wells 4 times. Add 100 µl of *Stabilized Chromogen* to each well. The liquid in the wells will begin to turn blue. Incubate for 30 minutes at room temperature and in the dark. The incubation time for chromogen substrate is often determined by the microtiter plate reader used. Many plate readers have the capacity to record a maximum optical density (O.D.) of 2.0. The O.D. values should be monitored, and the substrate reaction stopped before the O.D. of the positive wells exceeds the limits of the instrument. The O.D. values at 450 nm can only be read after the *Stop Solution* has been added to each well. If using a reader that records only to 2.0 O.D., stopping the assay after 20 to 25 minutes is suggested. Add 100 µl of *Stop Solution* to each well. Tap side of plate gently to mix. The solution in the wells should change from blue to yellow. Read the absorbance of each well at 450 nm having blanked the plate reader against a chromogen blank composed of 100 µl each of *Stabilized Chromogen* and *Stop Solution*. Read the plate within 2 hours after adding the *Stop Solution*. Use a curve fitting software to generate the standard curve. A four-parameter algorithm provides the best standard curve fit. Read the concentrations for unknown samples and controls from the standard curve. Multiply value(s) obtained for sample(s) by the appropriate dilution factor to correct for the dilution in step 3. Samples producing signals greater than that of the highest standard should be diluted in *Standard Diluent Buffer* and reanalyzed.

4.2.6. Caspase-8 activation assay

Cells were obtained from American Type Culture Collection, cells were grown in RPMI 1640 containing 10% fetal bovine serum at 37°C, stimulated with the compounds to be tested for caspase8, and lysed with Cell Extraction Buffer. This lysate was diluted in Standard Diluent Buffer over the range of the assay and measured for human active caspase-8 content. (*Cells are Plated in a density of 1.2 – 1.8 × 10,000 cells/well in a volume of 100µl complete growth medium + 100 ul of the tested compound per well in a 96-well plate for 24 hours before the enzyme assay for Tubulin.*). The absorbance of each microwell was read on a spectro-photometer at 450 nm. A standard curve is prepared from 7human Caspase-8 standard dilutions and human Caspase-8 concentration determined.

4.2.7. Bax activation assay

Bring all reagents, except the human Bax-α Standard, to room temperature for at least 30 minutes prior to opening. The human Bax-α Standard solution should not be left at room temperature for more than 10 minutes. All standards, controls and samples should be run in duplicate. Refer to the Assay Layout Sheet to determine the number of wells to be used and put any remaining wells with the desiccant back into the pouch and seal the ziploc. Store unused wells at 4 °C. Pipet 100 µL of Assay Buffer into the S0 (0 pg/mL standard) wells. Pipet 100 µL of Standards #1 through #6 into the appropriate wells. Pipet 100 µL of the Samples into the appropriate wells. Tap the plate gently to mix the contents. Seal the plate and incubate at room temperature on a plate shaker for 1 hour at ~500 rpm. Empty the contents of the wells and wash by adding 400 µL of wash solution to every well. Repeat the wash 4 more times for a total of 5 washes. After the final wash, empty or aspirate the wells and firmly tap the plate on a lint free paper towel to remove any remaining wash buffer. Pipet 100 µL of yellow Antibody into each well, except the Blank. Seal the plate and incubate at

room temperature on a plate shaker for 1 hour at ~500 rpm. Empty the contents of the wells and wash by adding 400 µL of wash solution to every well. Repeat the wash 4 more times for a total of **5** washes. After the final wash, empty or aspirate the wells and firmly tap the plate on a lint free paper towel to remove any remaining wash buffer. Add 100 µL of blue Conjugate to each well, except the Blank. Seal the plate and incubate at room temperature on a plate shaker for 30 minutes at ~500 rpm. Empty the contents of the wells and wash by adding 400 µL of wash solution to every well. Repeat the wash 4 more times for a total of **5 washes**. After the final wash, empty or aspirate the wells and firmly tap the plate on a lint free paper towel to remove any remaining wash buffer. Pipet 100 µL of Substrate Solution into each well. Incubate for 30 minutes at room temperature on a plate shaker at ~500 rpm. Pipet 100 µL Stop Solution to each well. Blank the plate reader against the Blank wells, read the optical density at 450 nm. Calculate the average net Optical Density (OD) bound for each standard and sample by subtracting the average Blank OD from the average OD for each standard and sample. Using linear graph paper, plot the Average Net OD for each standard versus Bax concentration in each standard. Approximate a straight line through the points. The concentration of Bax in the unknowns can be determined by interpolation.

4.2.8. Bcl-2inhibition assay

Mix all reagents thoroughly without foaming before use. Wash the microwells twice with approximately 300 µL Wash Buffer per well with thorough aspiration of microwell contents between washes. Take caution not to scratch the surface of the microwells. After the last wash, empty the wells and tap microwell strips on absorbent pad or paper towel to remove excess Wash Buffer. Use the microwell strips immediately after washing or place upside down on a wet absorbent paper for not longer than 15 minutes. Do not allow wells to dry. Add 100 µL of Sample Diluent in duplicate to all standard wells and to the blank wells. Prepare standard (1:2 dilution) in

duplicate ranging from 32 ng/mL to 0.5 ng/mL. Add 100 µL of Sample Diluent, in duplicate, to the blank wells. Add 80 µL of Sample Diluent, in duplicate, to the sample wells. Add 20 µL of each Sample, in duplicate, to the designated wells. Add 50 µL of diluted biotin-conjugate to all wells, including the blank wells. Cover with a plate cover and incubate at room temperature, on a microplate shaker at 100 rpm if available, for 2 hours. Remove plate cover and empty the wells. Wash microwell strips 3 times as described in step 2. Add 100 µL of diluted Streptavidin-HRP to all wells, including the blank wells. Cover with a plate cover and incubate at room temperature, on a microplate shaker at 100 rpm if available, for 1 hour. Remove plate cover and empty the wells. Wash microwell strips 3 times as described in step 2. Proceed to the next step. Pipette 100 µl of mixed TMB Substrate Solution to all wells, including the blanks. Incubate the microwell strips at room temperature (18° to 25°C) for about 15 minutes, if available on a rotator set at 100 rpm. Avoid direct exposure to intense light. The point, at which the substrate reaction is stopped, is often determined by the ELISA reader. Many ELISA readers record absorbance only up to 2.0 O.D. Therefore, the color development within individual microwells must be watched by the person running the assay and the substrate reaction stopped before positive wells are no longer properly detectable. Stop the enzyme reaction by quickly pipetting 100 µL of Stop Solution into each well, including the blank wells. It is important that the Stop Solution is spread quickly and uniformly throughout the microwells to completely inactivate the enzyme. Results must be read immediately after the Stop Solution is added or within one hour if the microwell strips are stored at 2 - 8°C in the dark. Read absorbance of each microwell on a spectrophotometer using 450 nm as the primary wavelength.

4.3. Statistical analysis

Computerized Prism 5 program was used to statistically analyzed data using one-way ANOVA test followed by Tukey's as post ANOVA for multiple comparison at $P \leq .05$. Data were presented as mean ± SEM.

4.4. Docking Study

All the molecular modeling calculations and docking simulation studies were performed on a Processor Intel(R) Pentium(R) CPU N3510@ 1.99GHz and 4 GB Memory with Microsoft Windows 8.1 pro (64 Bit) operating system using Molecular Operating Environment (MOE 2019.0102, 2020; Chemical Computing Group, Canada) as the computational software. All MOE minimizations were performed until a RMSD gradient of 0.01 kcal/mol/Å with the force field (OPLS-AA) to calculate the partial charges automatically using Born solvation. Before simulations, the protein was corrected, hydrogens are added and ionization states assigned, the system was optimized via protonation, distant water molecules were deleted, and receptor was minimized using QuickPrep function. Then, both ligand and pocket were isolated in 3D and molecular surface was drawn around the binding site to visualize the space available for docked ligands. The compounds' database was created after the compounds' structures were prepared, partially charged, and minimized. Initially, Self-docking was performed, and ligand conformations are generated with the bond rotation method. These are then placed on the site with the Triangle Matcher method and ranked with the London dG scoring function. The Retain option specifies the number of poses (30) to pass to the Refinement, for energy minimization in the pocket, before restoring with the GBVI/WSA dG scoring function. Then, co-crystallized ligand was deleted, dummy atoms were created at the site using the Site Finder application. Docking simulation was performed using compound database mdb file. A triangle matching with London dG scoring was chosen for initial

placement, and then the top 30 poses were refined using force field (OPLS-AA) and GBVI/WSA dG scoring. The output database dock file was created with different poses for each ligand and arranged according to the final score function (S), which is the score of the last stage that was not set to zero.

PDB: 1M17 was chosen owing to being a X-ray crystal structure of homo sapiens EGFR in complex with an inhibitor (erlotinib) of better Resolution (2.60 Å) with a little higher R-Value free of 0.295. Also, PDB: 4ASD was chosen owing to being a X-ray crystal structure of homo sapiens VEGFR-2 in complex with an inhibitor (sorafenib) of better Resolution (2.03 Å) with good R-Value free of 0.230. Moreover, erlotinib as well as sorafenib were used as reference drugs during the experimental enzyme inhibition assays and it was a good comparison to perform *in silico* docking studies using erlotinib and sorafenib as co-crystalized ligands.

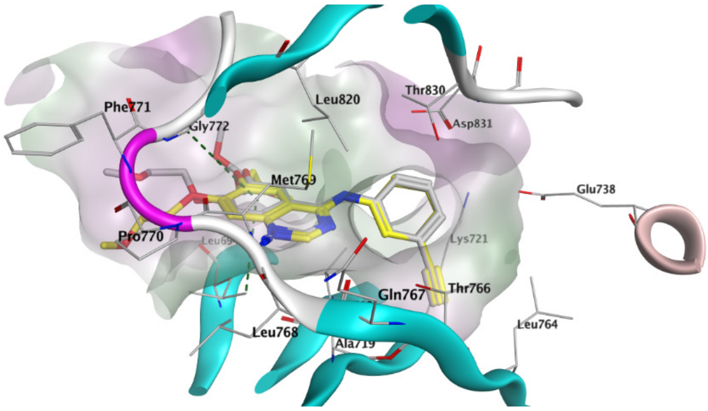

Figure S1: 3D binding mode of the redocked ligand (erlotinib) (white) into the active site of $EGFR^{WT}$ (PDB: 1M17) overlaid with the co-crystallized ligand (yellow), *RMSD* = 1.47 Å.

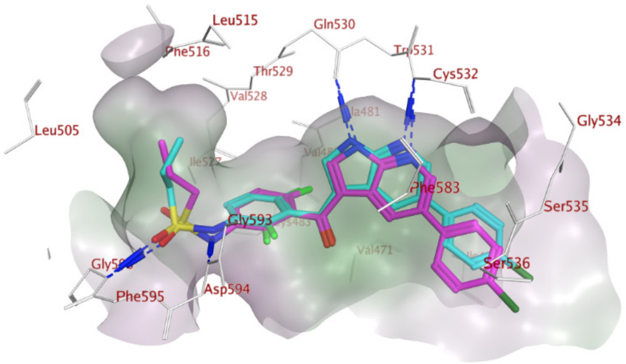

Figure S2: 3D binding mode of the redocked ligand (Vemurafenib) (cyan) into the active site of BRAF^{V600E} (PDB: 3OG7) overlaid with the co-crystallized ligand (purple), *RMSD* = 0.96 Å.

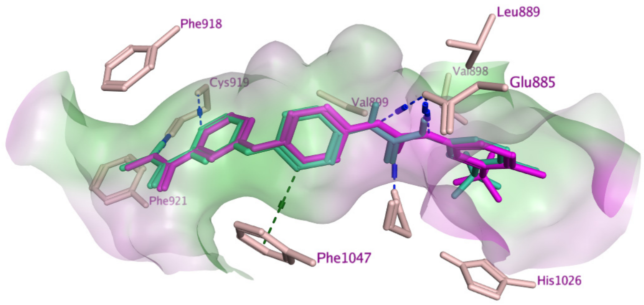

Figure S3: 3D binding mode of the redocked ligand (sorafenib) (cyan) into the active site of VEGFR (PDB: 4ASD) overlaid with the co-crystallized ligand (purple), *RMSD* = 0.46 Å.
